# Tripartite chimeric pseudogene from the genome of rice blast fungus *Magnaporthe grisea *suggests double template jumps during long interspersed nuclear element (LINE) reverse transcription

**DOI:** 10.1186/1471-2164-8-360

**Published:** 2007-10-08

**Authors:** Elena Gogvadze, Crystel Barbisan, Marc-Henri Lebrun, Anton Buzdin

**Affiliations:** 1 Shemyakin-Ovchinnikov Institute of Bioorganic Chemistry, Moscow 117871, Russia; 2 UMR 5240 CNRS, Université Claude Bernard Lyon1, INSA, Bayer CropScience, 14-20 rue Pierre Baizet, 69263 Lyon Cedex 09, France

## Abstract

**Background:**

A systematic survey of loci carrying retrotransposons in the genome of the rice blast fungus *Magnaporthe grisea *allowed the identification of novel non-canonical retropseudogenes. These elements are chimeric retrogenes composed of DNA copies from different cellular transcripts directly fused to each other. Their components are copies of a non protein-coding highly expressed RNA of unknown function termed WEIRD and of two fungal retrotransposons: MGL and Mg-SINE. Many of these chimeras are transcribed in various *M. grisea *tissues and during plant infection. Chimeric retroelements with a similar structure were recently found in three mammalian genomes. All these chimeras are likely formed by RNA template switches during the reverse transcription of diverse LINE elements.

**Results:**

We have shown that in *M. grisea *template switching occurs at specific sites within the initial template RNA which contains a characteristic consensus sequence. We also provide evidence that both single and double template switches may occur during LINE retrotransposition, resulting in the fusion of three different transcript copies. In addition to the 33 bipartite elements, one tripartite chimera corresponding to the fusion of three retrotranscripts (WEIRD, Mg-SINE, MGL-LINE) was identified in the *M. grisea *genome. Unlike the previously reported two human tripartite elements, this fungal retroelement is flanked by identical 14 bp-long direct repeats. The presence of these short terminal direct repeats demonstrates that the LINE enzymatic machinery was involved in the formation of this chimera and its integration in the *M. grisea *genome.

**Conclusion:**

A survey of mammalian genomic databases also revealed two novel tripartite chimeric retroelements, suggesting that double template switches occur during reverse transcription of LINE retrotransposons in different eukaryotic organisms.

## Background

Reverse transcription is one of the key processes that shape eukaryotic genomes. At least 40% of mammalian DNA was formed through reverse transcription [1-3]. This phenomenon was discovered when Temin and Baltimore purified and characterised retroviral RNA-dependant DNA polymerase (reverse transcriptase, RT), which catalyzes the synthesis of complementary DNA on RNA template [4]. Afterwards, RT sequences were found in very diverse genetic elements, termed retroelements (REs). All REs are transposable elements that proliferate through their RNA intermediates by using self-encoded or exogenous RT to synthesise the DNA copy of the element to be inserted into the host genome.

Retroelements carrying their own RT genes are autonomous REs that are classified into two major groups: long terminal repeat (LTR) containing elements, and non LTR retrotransposons [5]. Autonomous non LTR REs are generally assigned to LINEs, long interspersed nuclear elements. Among the REs, only LINEs are thought to be able to provide their RT enzyme for the proliferation of non autonomous REs [6]. LINEs have been found in essentially all eukaryotic DNAs [3,7]. LINE insertions are flanked by 10–20 bp long duplications called target site duplications (TSD). LINEs also contain an oligo (A) or microsatellite A-rich sequences at their 3' termini. Another LINE distinguishing feature is their frequent 5'-truncation. These truncations likely result from LINE RNA abortive reverse transcription, when RT dissociates from its RNA template before having completed full cDNA copy synthesis [8].

The full-sized LINE (+) RNA is both a transpositional RNA intermediate and the template for protein synthesis [9]. LINE transposition is known to proceed in several steps including Pol II transcription of an active element, reverse transcription of the RNA formed with the self-encoded RT, and integration of the cDNA into a new position within the genome [10]. Due to the so-called 'cis-preference', the enzymatic machinery of a retrotransposition-competent LINE predominantly transposes its own copies [11]. However, LINEs are capable of transposing other sequences, mostly non autonomous REs termed short interspersed nuclear elements (SINEs), but also cDNAs from different types of cellular RNAs, thus forming processed pseudogenes [12]. Recently we have shown a new property of the LINE reverse transcriptional machinery that is able to form bipartite chimeric elements during reverse transcription in mammalian and fungal genomes [13-17]. These elements are composed of DNA copies from cellular transcripts either directly fused to each other or more frequently fused to the 3' part of a LINE retroposon.

**Figure 1:**
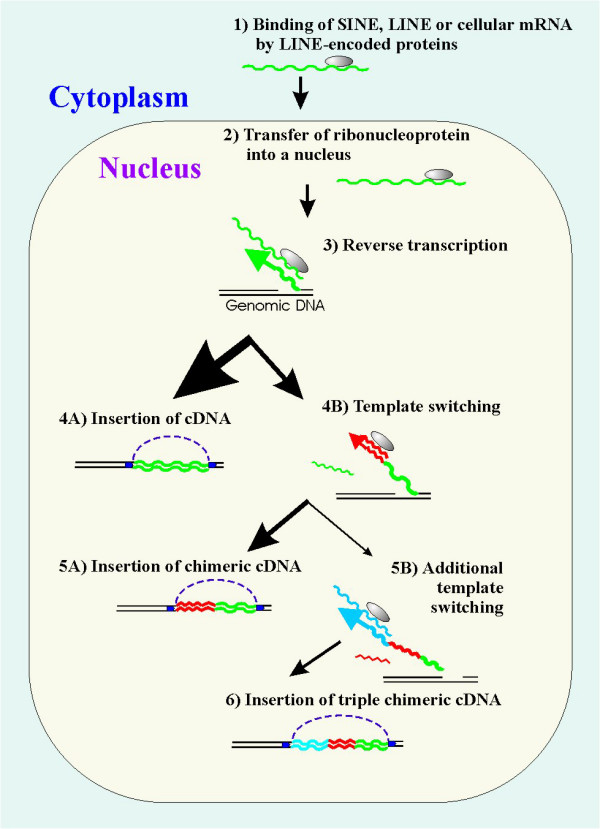
Mechanism for the chimeras' formation by LINE enzymatic machinery. (Step 1) LINE pre-integration complex binds LINE, SINE or RNA in the cytoplasm. (Step 2) The resulting ribonucleoprotein is transferred to the nucleus. (Step 3) Reverse transcription of the bound RNA primed by a genomic DNA single-stranded break (target site primed reverse transcription). (Step 4A) Successful integration of the reverse transcribed cDNA copy into the genomic DNA. (Step 4B) Switch of templates on another RNA during the reverse transcription. (Step 5A) Integration of the chimera formed into genomic DNA. (Step 5B) The second template switch to another RNA with subsequent DNA reparation mediates formation of a tripartite chimeric retrogene insertion flanked by short direct repeats. The normal LINE integration pathway is: steps (1) – (2) – (3) – (4A).

The various cellular transcripts found in these chimeras correspond to messenger RNAs, ribosomal RNAs, small nuclear RNAs, transposable elements and 7SL RNA. These chimeras have the following common features: (*i*) 5'-parts are full-length copies of cellular RNAs; (*ii*) 3'-parts are 5'-truncated copies of the corresponding RNAs (mostly LINEs); (*iii*) sites of these truncations occur at random in the corresponding RNA; (*iv*) both parts are directly joined with the same transcriptional orientation; (*v*) chimeras are flanked by short direct repeats. Many of the chimeras can be considered as new genes, as they were shown to be transcribed, some of them in a tissue-specific manner [13,16,18]. The most probable mechanism for the chimera formation involves RNA recombination during the reverse transcription of cellular RNAs (Figure 1). This model includes a switch of the RT complex with nascent cDNA from RNA serving as the initial template for the reverse transcription to another RNA corresponding to the 5' part of the chimera, followed by the chimera integration into the host genome [3].

This model of chimera formation was further supported by results obtained using experimentally controlled retrotransposition of human *L1 *LINE element *in vitro *[19] and *in vivo *[20]. Interestingly, it has been recently postulated that RT templates jump from LINE RNA to host genomic DNA facilitating integration, thus, being normally required for successful LINE retrotransposition [20,21]. In addition to the generation of chimeric retrogenes, template switching events during LINE reverse transcription could give rise to chimeric SINE elements [22] and to mosaic LINE structures. These events likely result from RNA recombination between different LINE templates [8,21,23,24].

More recently, two tripartite chimeric retroelements, each consisting of fused copies of three human RNAs, have been found in the human genome sequence. Formation of such tripartite retrogenes might result from double RNA template switching events during LINE retrotransposition [16]. However, no proof was provided for this concept, as both triple elements were inserted into A or AT-rich genomic sequences, making it impossible to identify direct repeats flanking the integrated element. In this report, we provide direct evidence for *in vivo *double template-switching in the genome of the rice blast fungus *Magnaporthe grisea*. We identified one tripartite chimeric retroelement in this fungal genome and showed that it is flanked by identical non-satellite 14 bp-long direct repeats. We also identified two similar tripartite chimeric retroelements in the mouse genome and found that WEIRD, the major component of fungal bipartite chimeric retroelements, is a non-coding sequence highly expressed in various *M. grisea *tissues and during plant infection. We have shown that template switching does not occur in *M. grisea *at random sites of the template RNA as thought for mammalian chimera formation [25]), but occurs at hot spots located downstream of specific sequence motifs. Lastly, this study allowed the identification of novel bipartite chimeric retroelements in *M. grisea*.

## Results and discussion

### Characterization of fungal chimeric retroelements

**Figure 2:**
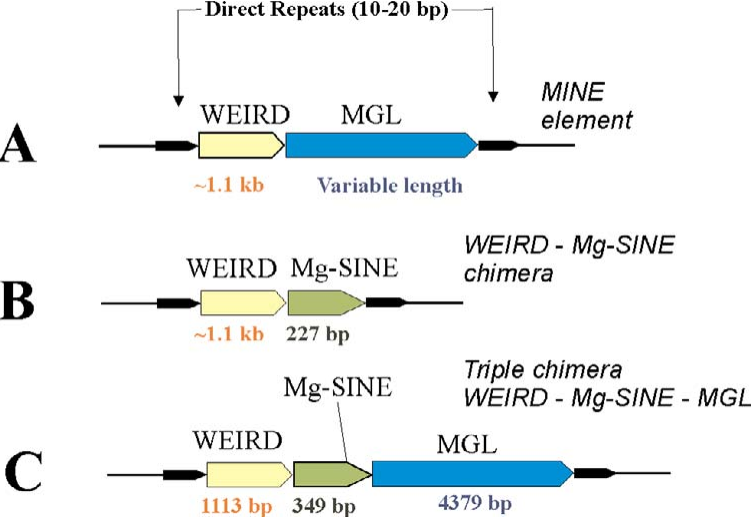
Schematic representation of the bipartite chimeric retrogenes identified in *M. grisea *genome. Inserts are flanked by 10–20 bp long genomic direct repeats.

Recently, a new family of bipartite chimeric retroelements termed MINE (Mixed Interspersed Nuclear Elements) has been identified in the genome of the rice blast fungus *Magnaporthe grisea *[17]. The 5' parts of MINE correspond to a full length copy of a 1.1 kb long non-coding transcript of unknown function called WEIRD (Figure. 2A). The 3' parts of MINE correspond to 5' truncated copies of the MGL LINE element [17]. The organization and diversity of MINE elements suggest that they are likely formed as a result of a template RNA switch during LINE reverse transcription and integrated into the genome using LINE machinery as proposed for similar mammalian LINE chimeras [3,13-15,18]. A copy of MINE was found as a recent insertion in *ACE1 *avirulence gene [17,26], showing that this mechanism is still active in fungal cells and could inactivate genes. In order to investigate the distribution of bipartite retrogenes and to find out if other types of chimeras than MINE were generated in *M. grisea*, we performed a systematic bioinformatic analysis of *M. grisea *genome sequence for all possible WEIRD and MGL sequences that are not directly flanked by short direct repeats (target site duplications). Detailed analysis of genomic sequences flanking these elements allowed the identification of 33 chimeric retroelements flanked by 10–20 bp long direct repeats (Figure 2). As expected, the majority (31 elements) of these chimeras belong to the MINE family (WEIRD – MGL chimeras). 19 MINEs were flanked by target site duplications while the direct repeats could not be found for the 12 other MINEs as one of their ends correspond to a gap in *M. grisea *genomic sequence. Compared to a survey performed on a previous version of *M. grisea *genome [17], we only identified one novel MINE copy.

In contrast to mammalian bipartite chimeric retroelements that have a 5' cellular component corresponding to known nucleolar RNAs (mostly U6 snRNA), the 5' cellular component of *M. grisea *bipartite chimeric retroelements corresponds mainly to a non-coding RNA (WEIRD) that has no homology to *M. grisea *U6 snRNA [Gogvadze et al., unpublished data]. The molecular function of WEIRD RNA remains unknown. In a series of *in situ *hybridization experiments with WEIRD specific oligonucleotide probes, we were not able to define WEIRD intracellular localization due to extremely poor permeability of the *M. grisea *cell wall [Gogvadze et al., unpublished data]. Expression of WEIRD was assessed in different *M. grisea *tissues (mycelium, spore) during a barley leaf infection using real-time quantitative reverse transcription-PCR, where expression was relative to two reference fungal housekeeping genes. Levels of WEIRD RNA are comparable to those of the housekeeping gene *Ilv5 *(35, 20 and 61% of *Ilv5 *transcript level in different tissues). WEIRD was differentially expressed, being mostly up-regulated during the infection process (2-fold versus mycelium, Table 1), whereas MGL was up-regulated in spores. To address whether WEIRD is a unique genomic sequence or a new transposable element, we performed a bioinformatic analysis of *M. grisea *genome for WEIRD-like sequences. Surprisingly, no solo WEIRD copies were found, as all of the WEIRD sequences we identified were directly fused with MGL LINE, forming MINE retrogenes. The absence of the initial WEIRD gene in the databases is probably due to the still incomplete state of *M. grisea *genome sequencing [27]. No sequences with significant similarities with WEIRD were found in other available genomes. Therefore, WEIRD (i) is not a transposable element, (ii) is expressed in mycelium/spores and during infection, and (iii) does not encode for a protein. These properties suggest that WEIRD might be a functional housekeeping non-coding RNA.

**Table 1:** Expression of WEIRD and MGL relatively to housekeeping genes *Ilv5 *and *Ef1*

	**Housekeeping gene**	**Mycelium**	**Spores**	**0 hours infection**	**8 hours infection**	**24 hours infection**
**WEIRD**^a^	*Ef1 alpha*	1.6 ± 0.5	1.7 ± 0.8	-	1.0 ± 0.4	2.5 ± 0.8
	*Ilv5*	35. 2 ± 7.8	20.6 ± 5.2	-	35.0 ± 6.5	61.2 ± 12.2
**MGL**^a^	*Ef1 alpha*	2.8 ± 0.9	8.9 ± 2.0	-	1.3 ± 0.7	2.4 ± 0.4
	*Ilv5*	60.2 ± 14.5	108.2 ± 14.3	-	44.0 ± 6.6	58.2 ± 10.9

^a^Relative transcription, % of the corresponding housekeeping gene transcript level. All quantitative RT-PCR experiments were performed in quadruplicate. Relative transcription was calculated according to the formula: 100% × 2^ΔC(t)^, where 2−ΔCt=2−(Ctsample−Ctref)
 MathType@MTEF@5@5@+=feaafiart1ev1aaatCvAUfKttLearuWrP9MDH5MBPbIqV92AaeXatLxBI9gBaebbnrfifHhDYfgasaacH8akY=wiFfYdH8Gipec8Eeeu0xXdbba9frFj0=OqFfea0dXdd9vqai=hGuQ8kuc9pgc9s8qqaq=dirpe0xb9q8qiLsFr0=vr0=vr0dc8meaabaqaciaacaGaaeqabaqabeGadaaakeaacqaIYaGmdaahaaWcbeqaaiabgkHiTiabfs5aejabboeadjabbsha0baakiabg2da9iabikdaYmaaCaaaleqabaGaeyOeI0IaeiikaGIaee4qamKaeeiDaq3aaSbaaWqaaiabdohaZjabdggaHjabd2gaTjabdchaWjabdYgaSjabdwgaLbqabaWccqGHsislcqqGdbWqcqqG0baDdaWgaaadbaGaemOCaiNaemyzauMaemOzaygabeaaliabcMcaPaaaaaa@4A0C@.

### Novel non-canonical retropseudogenes from the genome of *M. grisea*

Apart from the previous 31 MINE*s*, we identified one novel bipartite chimeric retroelement with a WEIRD sequence fused to 5'-truncated Mg-SINE. This WEIRD-MgSINE retroelement was flanked by 12 bp-long direct repeats (figure 2B, Additional file 1). Mg-SINE is a non-autonomous SINE-family retroelement [28]. It likely utilizes a "molecular mimicry" to MGL for its proliferation. Indeed, the 3' end of Mg-SINE is similar to the 3' end of MGL retrotransposon used for priming reverse transcription [29,30]. This property of Mg-SINE sequence likely results in the capture of Mg-SINE transcripts by the MGL retrotranspositional apparatus.

We have also identified a novel chimera composed of three elements (figure 2C, Additional file 1). Its 5' component is a full length WEIRD fused to a 5'-truncated Mg-SINE that is itself fused to a 5'-truncated MGL. The tripartite chimeric retroelement is flanked by 14 bp-long direct repeats showing that it was integrated into the *M. grisea *genome as a single chimeric retrogene likely formed by two successive template RNA switches during the reverse transcription of MGL.

Overall, this survey of *M. grisea *genome showed that most chimeric retrogenes containing MGL sequences are represented by MINEs(WEIRD-MGL), although we also identified another type of bipartite chimeras (WEIRD-MgSINE) and a triple chimera (WEIRD-MgSINE-MGL).

### Several fungal chimeric retrogenes are transcribed

**Figure 3:**
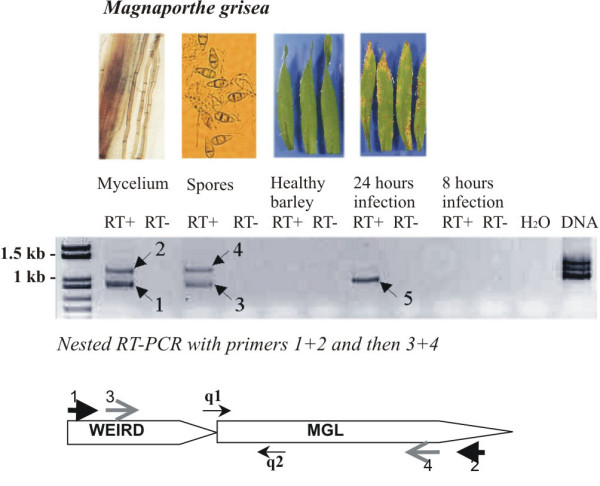
MINE transcripts observed at different stages of *M. grisea *life cycle. Products from nested non-quantitative RT-PCR were separated by agarose gel electrophoresis and visualized by ethidium bromide staining (negative image). Nested PCR was performed to increase the amplification specificity. Arrows on the bottom of the figure indicate primer binding sites within the MINE element. To identify transcribed chimeras, one round of nested non-quantitative RT-PCR with primer pairs 1+2 and then 3+4 was used, followed by isolation and sequencing of the resulting RT-PCR products (bands 1–5, on the middle). To further specifically amplify each individual chimeric element in quantitative reverse transcription-PCR experiments, a combination of primers q1+q2 was used. Primer q1 is specifically designed to the WEIRD-MGL junction site of the desired MINE element.

To assess if these fungal bipartite chimeric retroelements were transcribed, we used reverse transcription-PCR (RT-PCR) with MINE specific primers designed to the 5' terminus of WEIRD (in the sense orientation), and to the 3' terminus of MGL (in the antisense orientation; Figure 3). Some MINE chimeras were expressed in mycelium, spores and 24 hours old infected leaves. Sequencing of these RT-PCR products revealed that bands 1 (mycelium) and 5 (24 hours infection) correspond to transcripts from the same MINE chimera as well as bands 2 (mycelium) and 4 (spores) that correspond to another MINE chimera. Overall, we identified three transcriptionally active MINEs in *M. grisea*: MINE-A (bands 1 and 5), MINE-B (bands 2 and 4) and MINE-C (band 3) that correspond, respectively, to the elements 4, 10 and 3 we identified in the *M. grisea *genome (Additional file 1; GenBank accession numbers EF585235–EF585237). These three transcribed chimeras are likely an underestimation of the possible MINE transcripts, as many of these elements have a long MGL part (up to 5 kb) that is likely too long to be efficiently amplified by RT-PCR. To further quantify the mRNA levels of these MINE*s*, we performed quantitative real-time PCR experiments with primers specific for each WEIRD-MGL junctions (primers q1 and q2, Figure 3) to specifically amplify each chimera (Table 2). MINE-A, MINE-B and MINE-C are transcribed in all analyzed tissues, but at different levels. MINE-B is constitutively expressed in all tissues tested including infection, while MINE-A and MINE-C are up-regulated during infection as observed for WEIRD (× 2 vs mycelium) and MINE-A is down-regulated in spores (× 0.5 vs mycelium). These experiments demonstrate that some WEIRD-MGL chimeric elements are transcribed and that the expression pattern differs depending on *M. grisea *tissues. Consequently, these expressed chimeras may be involved in a particular cell function.

**Table 2:** MINE-A, -B and -C expression relatively to housekeeping genes *Ilv5 *and *Ef1*

**Tissue**	**MINE-A**		**MINE-B**		**MINE-C**	
	
	R.t.^a ^(*Ef1alpha*)	R.t.^a ^(*Ilv5*)	R.t.^a ^(*Ef1alpha*)	R.t.^a ^(*Ilv5*)	R.t.^a ^(*Ef1alpha*)	R.t.^a ^(*Ilv5*)
**mycelium**	4.4 ± 1.6	70.1 ± 14.5	4.4 ± 0.2	71.2 ± 5.1	1.4 ± 0.1	24.1 ± 8.5
**spore**	2.1 ± 0.4	20.0 ± 0.7	4.7 ± 0.5	44.6 ± 1.7	2.8 ± 0.3	26.4 ± 1.0
**infection 24 hours**	10.4 ± 1.0	154.3 ± 17.8	4.3 ± 1.24	64.4 ± 10.2	3.1 ± 0.6	42.7 ± 7.4

^a^Relative transcription, % of the corresponding housekeeping gene transcript level. To specifically amplify each individual chimeric element, a combination of primers q1+q2 was used (See Figure 3). Primer q1 is specifically designed to the WEIRD-MGL junction site of the desired MINE element. All quantitative RT-PCR experiments were performed in quadruplicate. Relative transcription values were calculated as described on the legend to the table 1.

### Tripartite chimeric retroelement: evidence for double template switching model

**Figure 4:**
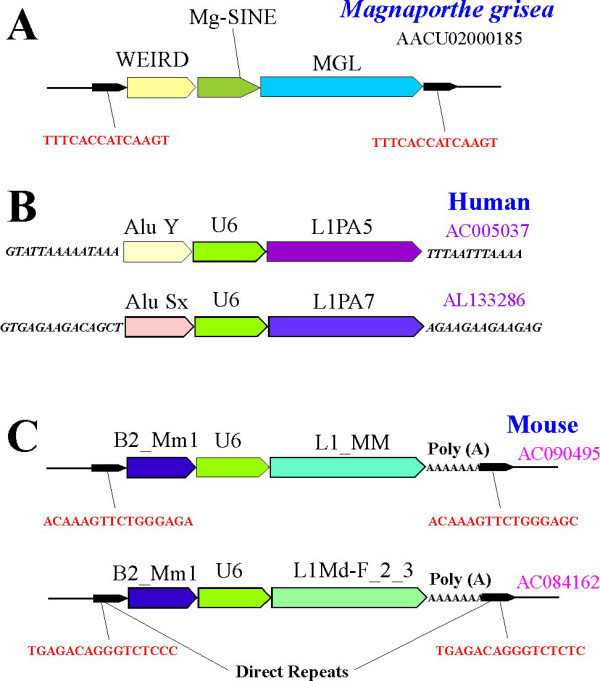
Schematic representation of the tripartite elements found in *M. grisea *(A), human (B) and mouse (C) genomes. Only fungal and mouse tripartite retrogenes are flanked by the direct repeats and, therefore, can be regarded as the tripartite chimeric retroelements.

The fungal tripartite chimeric retroelement discovered in this study (Figure 4A) suggests that the suspected mechanism of LINE-mediated *in vivo *RNA recombination is not limited to a single template switch. The most probable explanation for the formation of a tripartite chimeric retroelement is a double template switch during LINE retrotransposition (Figure 1). Having reverse-transcribed 3'-terminal 4369 nucleotides of MGL LINE (the total length of MGL is ~6 kb), the retrotranspositional complex switched to another RNA (located nearby or captured by the RT/integrase assembly) and added a ~350 nt-long 3' fragment of the Mg-SINE element to the nascent cDNA. RT dissociation from its initial template can be explained, for example, by reverse-transcriptional pausing events [31], or by a damage to the initial RNA strand [21]. However, before the completion of Mg-SINE reverse transcription, RT changed template for a second time, and synthesized a full copy of WEIRD RNA (1113 nt-long) on the terminus of the nascent first-strand-cDNA. After ligation, synthesis of the second cDNA strand and genomic DNA repair, the newly formed tripartite retrogene became flanked by 14 bp-long direct repeats resulting from the duplication of the genomic target site.

Previously, two tripartite chimera-like elements were identified in human DNA [16,18] (Fig. 4B). One of them was inserted in an expanded microsatellite locus, while the other was flanked by AT-rich sequences. As a result, the identification of direct repeats that should normally flank the chimera was not possible for these elements (Additional file 2). However, a survey of mammalian genomic databases allowed us to identify two new tripartite chimeric retrogenes flanked by perfect 15- and 16 bp-long direct repeats in the mouse genome (Fig. 4C, Additional file 2). These chimeras were composed of (5' to 3') *B2 *SINE, U6 snRNA and *L1 *LINE elements. Finding tripartite chimeric retroelements displaying considerable structural similarities in such evolutionary distinct organisms as mammals and fungi suggests that double template switches during LINE reverse transcription is probably not the exceptional case, but might be a conserved mechanism of eukaryotic DNA rearrangement.

### Template switching occurs at specific positions within template RNAs in *M. grisea*

The 3' terminal parts of fungal chimeric retroelements are usually composed of 3' fragments from MGL LINE retrotransposon. These fragments vary in length from 230 to 5461 3'-terminal MGL nucleotides. Analysis of the sequence of 33 chimeric retroelements allowed us to retrieve 28 different chimerization sites within the MGL sequence. In six cases, the same chimerization site was shared by two distinct chimeric retroelements (Additional file 1, pairs of elements: 5 & 6; 8 & 9; 10 & 11; 12 & 13; 4 & 15; 17 & 18). This observation showed that template switching occurred exactly at the same nucleotide position of the template RNA during the formation of six different pairs of chimeric retroelements. Additionally, analysis of the 28 chimerization sites revealed that template switching did not occur at random within template RNAs. Furthermore, highlighted hot spots within MGL corresponded to a conserved sequence (Table 3). Importantly, this motif was also found at the chimerization sites of MgSINE chimeric retroelements. The consensus sequence located at the chimerization sites on these template RNAs is GCC*A/U, where * indicates the site of the possible template switching. This observation suggests that MGL RT reverse-transcribes the template RNA until the A/U nucleotide of the motif, where it can jump to another RNA before the GCC triplet. It should be noted that the full-length MGL RNA has 87 GCC(A/U) sites and far more derivatives with single nucleotide substitutions, whereas only a small portion of these motifs were used as chimerization sites *in vivo*. Taking into account that six of these sites are hot spots for "RNA recombination" we conclude that this motif is necessary but not sufficient for the template switching. It seems reasonable to postulate that the chimerization sites are located in a region of the template RNA with a secondary structure that could lead to a pause in reverse transcription. This situation has already been observed for template switching sites of mammalian chimeric retrogenes that coincide with reverse-transcription pausing sites [25,31]. *In silico *prediction of MGL RNA secondary structures around chimerization sites of fungal chimeric retrogenes predicts strong hairpin structures immediately upstream of known template switching sites (Additional file 3) that theoretically could slow down reverse transcription.

**Table 3:** Representative chimerization sites found in *M. grisea*

**Element ID**^a^	**RNA template**^b^	**Upstream sequence**^c^	**Downstream sequence**^d^
34	MGL	CGCAGC	ATTCG
24	MGL	TATGCT	ATATT
23	MGL	AGCGCC	TCCGG
4	MGL	GGGGTC	TTAGA
15	MGL	GGGGTC	TTAGA
14	MGL	TGGGCC	TTTCT
13	MGL	GGAGCC	CGAGG
12	MGL	GGAGCC	CGAGG
9	MGL	TTGGCC	ATGAG
8	MGL	TTGGCC	ATGAG
6	MGL	ATAGCC	ACCAA
5	MGL	ATAGCC	ACCAA
2	MGL	ACTGCC	TTTTC
1	MGL	GCCGTC	AGACG
7	MGL	CAAGCT	TCGGG
16	MGL	TGGGCC	GCTTT
32	MgSINE	GCCGTC	AGACG
33	MgSINE	CCCGCC	TGTGC
Consensus sequence	All types	---GCC	A/T----

^a^See Additional file 1 for the detailed description of each chimeric element.

^b^Type of the template RNA molecule.

^c^Upstream nucleotide sequence, relatively to the template switching site.

^d^Downstream nucleotide sequence, relatively to the template switching site.

It remains unclear why the RT dissociates from the template RNA exactly before the GCC triplet. It is known for many RTs that the enzyme processivity depends greatly on the nucleotide composition of the template RNA. It can be hypothesized that this GCC motif, in particular when located in a hairpin, is a "difficult place" for the MGL RT, inducing a reverse-transcriptional pause or even the RT to dissociate or to jump on another RNA template.

Finally, it should be mentioned that the above chimerization mechanism does not include any kind of specific nucleotide basepairing between the nascent cDNA and the second RNA template. Indeed, we were not able to find extended- or micro-homologies between the templates around the chimerization sites.

## Conclusion

In this paper, we demonstrate that several forms of chimeric retroelements are present in the *Magnaporthe grisea *genome. For the first time, we provide evidence that a triple chimera was generated *in vivo *and integrated into the *M. grisea *genome. The most probable mechanism for the formation of this chimera is template switching during LINE-mediated reverse transcription. Therefore, bipartite and tripartite chimeric retroelements likely result, respectively, from single and double template RNA switches during reverse transcription. Several chimeric retroelements are transcribed in *M. grisea*. The major fungal chimera components are MGL LINE retrotransposon and WEIRD. WEIRD does not encode for a protein and is not a transposable element. It is expressed at a high level in mycelium and spores and is up-regulated during plant infection. We hypothesize that WEIRD is a functional non-coding RNA. We have also identified novel chimeras including a novel LINE retrotransposon *Enigma *and fungal Mg-SINE element. To conclude, we have shown that in *M. grisea *template switching during reverse transcription occurs at specific sites within the initial template RNA, and a consensus sequence for these chimerization sites is proposed.

## Methods

### DNA sequence analysis

Homology searches against GenBank were done using the BLAST Web-server at NCBI [32]. Flanking regions of mammalian retroelements were investigated with the RepeatMasker program [33]. For fungal genomes, flanking regions were aligned with known filamentous fungal transposable elements reported elsewhere [29]. Direct repeats were detected by visual inspection of retroelement flanking sequences. Novel repeats were assigned to subfamilies according to the nomenclature proposed by Daboussi and Capy [29]. For multiple alignments, BLAST pairwise search, Vector NTI and Clustal W programs [34] were used.

### Fungal strains and growth conditions

Two *Magnaporthe grisea *strains – P1.2 and Guy11 – were used in this study. As all rice pathogenic *Magnaporthe grisea *isolates, these strains are also pathogenic on barley. Fungal strains were grown and stored as described [35].

### RT-PCR

Total RNAs were extracted from *M. grisea *mycelium, spores (strain P1.2) and infected barley leaves at 0 h, 8 h and 24 h (strain Guy11) using Rneasy Plus Mini Kit (Qiagen). cDNA synthesis was performed with 2 μg of total RNA using random primers and ThermoScript RT-PCR System (Invitrogen). The level of WEIRD and MGL transcription was assessed by real-time PCRs with genomic primers corresponding to the 5' and 3'-terminal parts of the elements using an ABI Prism 7700 Sequence Detection System (Applied Biosystems) and Power SYBR Green PCR Master Mix (Applied Biosystems); primers Wfor, Wrev and MGLfor, MGLrev for WEIRD and MGL, respectively. Primer sequences for real-time PCR were chosen using Primer Express software (Applied Biosystems) and are presented in supplementary material (Additional file 4). All measurments were carried out in quadruplicate and expression levels were normalized to *Ilv5 *and *Ef1 *using the following formulae 2−ΔCt=2−(Ctsample−Ctrefrence)
 MathType@MTEF@5@5@+=feaafiart1ev1aaatCvAUfKttLearuWrP9MDH5MBPbIqV92AaeXatLxBI9gBaebbnrfifHhDYfgasaacH8akY=wiFfYdH8Gipec8Eeeu0xXdbba9frFj0=OqFfea0dXdd9vqai=hGuQ8kuc9pgc9s8qqaq=dirpe0xb9q8qiLsFr0=vr0=vr0dc8meaabaqaciaacaGaaeqabaqabeGadaaakeaacqaIYaGmdaahaaWcbeqaaiabgkHiTiabfs5aejabboeadjabbsha0baakiabg2da9iabikdaYmaaCaaaleqabaGaeyOeI0IaeiikaGIaee4qamKaeeiDaq3aaSbaaWqaaiabdohaZjabdggaHjabd2gaTjabdchaWjabdYgaSjabdwgaLbqabaWccqGHsislcqqGdbWqcqqG0baDdaWgaaadbaGaemOCaiNaemyzauMaemOzayMaemOCaiNaemyzauMaemOBa4Maem4yamMaemyzaugabeaaliabcMcaPaaaaaa@50D3@[36]. All RT-PCR experiments were performed against the negative RT "-" controls (no reverse transcriptase added at the stage of the first strand cDNA synthesis) to control the DNA contamination. Only those samples displaying negative results on the RT "-" control experiments were further analyzed. To identify transcriptionally active MINEs a general RT-PCR was performed using primers designed to the 5' terminal part of WEIRD (in the sense orientation), and to the 3' end of MGL (in the reverse orientation) under the following conditions: 95°C – 2 min; 95°C – 25 s, 62°C – 25 s, 72°C – 2 min; 25 cycles. The products of RT-PCR were then diluted into 40 times and used as a template for the nested PCR (95°C – 2 min; 95°C – 25 s, 62°C – 25 s, 72°C – 2 min; 25 cycles). Nested PCR was performed to reduce the background originated due to the use of primers corresponding to repetitive sequences. The obtained products were sequenced by Genome Expressed (Meylan, France). Three transcriptionally active MINEs identified during this study were further analyzed by real-time RT-PCR using primers designed for the specific WEIRD-MGL junctions (q1 and q2 for MINE1; q3 and q4 for MINE2; q5 and q6 for MINE3).

## Authors' contributions

EG carried out the bioinformatic survey of *M. grisea *genomic databases, participated in the design of this study and performed the molecular genetic experiments. CB participated in the design of all experimental strategies employed throughout the research and contributed greatly to the immunocytochemistry and qRT-PCR experiments. MHL and AB conceived of the study, participated in its design and coordination, performed the statistical analysis and wrote the paper. All authors read and approved the final manuscript.

## Supplementary Material

Additional file 1 Chimeric retroelements of rice blast fungus *Magnaporthe grisea*. The data provided represent the detailed sequence information on all chimeric retrotranscripts identified so far in *Magnaporthe grisea *genome. The data provided represent the detailed sequence information on the triple. Click here for file

Additional file 2 Mammalian triple elements. The data provided represent the detailed sequence information on the triple chimeric retrotranscripts identified in the mammalian (mouse and rat) genomes. Click here for file

Additional file 3 Putative RNA secondary structure elements, predicted upstream template switching sites. RNA secondary structure features, predicted upstream template switching sites using Mfold version 3 software. Click here for file

Additional file 4 Oligonucleotides used in the present study. Sequence information for the primers used for RT-PCR experiments in the present study. Click here for file
