# Supplementary material for: Tripartite chimeric pseudogene from the genome of rice blast fungus Magnaporthe grisea suggests double template jumps during long interspersed nuclear element (LINE) reverse transcription
Source: BMC Genomics. 2007 Oct 8;8:360. doi: 10.1186/1471-2164-8-360 (PMC2104539; doi:10.1186/1471-2164-8-360)
Supplement: Additional file 1 — Chimeric retroelements of rice blast fungus Magnaporthe grisea. The data provided represent the detailed sequence information on all chimeric retrotranscripts identified so far in Magnaporthe grisea genome. The data provided represent the detailed sequence information on the triple. [file 1471-2164-8-360-S1.doc]

**Chimeric retroelements of rice blast fungus *Magnaporthe grisea*.**

**Table 1. MINEs and other chimeras from the *Magnaporthe grisea* genome.**

| Element ID | Accession number | Coordinates | 5' - part | 3' - part | Strand | Direct  repeats |
| --- | --- | --- | --- | --- | --- | --- |
| ***MINEs*** | | | | | | |
| 1 | AACU02000185 | 140238 - 141578 | WEIRD (1111nt) | MGL (230nt) | + | + |
| 2 | AACU02000635 | 56024 – 57398 | WEIRD (1113nt) | MGL (262nt) | - | + |
| 3 | AACU02000308 | 9698 - 11184 | WEIRD (1124nt) | MGL (363nt) | - | - |
| 4 | AACU02000436 | 64293 – 65785 | WEIRD (1111nt) | MGL (1025nt) | - | + |
| 5 | AACU02000380 | 47973 - 49468 | WEIRD (1111nt) | MGL (385nt) | + | + |
| 6 | AACU02000714 | 224443 – 225938 | WEIRD (1111nt) | MGL (385nt) | + | + |
| 7 | AACU02000527 | 2416 – 3954 | WEIRD (1114nt) | MGL (425nt) | + | + |
| 8 | AACU02000343 | 28159 - 29915 | WEIRD (1114nt) | MGL (643nt) | - | + |
| 9 | AACU02000467 | 3319 – 4429 | WEIRD (468nt) | MGL (643nt) | + | + |
| 10 | AACU02000098 | 991 - 2771 | WEIRD (1110nt) | MGL (671nt) | + | - |
| 11 | AACU02000173 | 96529-98316 | WEIRD (1117nt) | MGL (671nt) | - | - |
| 12 | AACU02000173 | 257655-259454 | WEIRD (1111nt) | MGL (689nt) | - | + |
| 13 | AACU02000326 | 82534 - 84341 | WEIRD (1119nt) | MGL (689nt) | - | + |
| 14 | AACU02000681 | 118115 – 120221 | WEIRD (1116nt) | MGL (991nt) | + | + |
| 15 | AACU02000436 | 32524 – 34661 | WEIRD (1111nt) | MGL (1027nt) | - | + |
| 16 | AACU02000589 | 55776 – 58085 | WEIRD (1111nt) | MGL (1199nt) | + | + |
| 17 | AACU02000448 | 97298 - 99813 | WEIRD (1118nt) | MGL (1398nt) | + | - |
| 18 | AACU02000448 | 3 – 2513 | WEIRD (1110nt) | MGL (1400nt) | - | - |
| 19 | AACU02000346 | 8097 - 10724 | WEIRD (1112nt) | MGL (1516nt) | - | - |
| 20 | AACU02000308 | 24615 - 27415 | WEIRD (1126nt) | MGL (1675nt) | + | - |
| 21 | AACU02000649 | 22755 – 25729 | WEIRD (1112nt) | MGL (1863nt) | - | + |
| 22 | AACU02000525 | 31188 – 34204 | WEIRD (1111nt) | MGL  (1906) | - | - |
| 23 | AACU02000324 | 34317 - 37342 | WEIRD (1111nt) | MGL (1915nt) | + | + |
| 24 | AACU02000514 | 14699 - 18014 | WEIRD (1111nt) | MGL (2004nt) | + | + |
| 25 | AACU02000457 | 390979 - 394653 | WEIRD (1111nt) | MGL (2563nt) | + | - |
| 26 | AACU02000708 | 16696 – 20527 | WEIRD (1111nt) | MGL (2721nt) | - | + |
| 27 | AACU02000560 | 15483 - //* | WEIRD (?) | MGL (3145nt) | - | ? |
| 28 | AACU02000591 | 4361 – 8975 | WEIRD (1118nt) | MGL (3497nt) | + | + |
| 29 | AACU02000397 | 8086 - 13226 | WEIRD (1110nt) | MGL (4031nt) | - | - |
| 30 | AACU02000227 | 4251 - 9758 | WEIRD (1112nt) | MGL (4396) | + | - |
| 31 | AACU02000381 | 27064 - 33050 | WEIRD (1113nt) | MGL (4874nt) | + | + |
| ***Other chimeras*** | | | | | | |
| 32 | AACU02000348 | 160236 - 161572 | WEIRD (1110nt) | Mg-SINE (227nt) | - | + |

***Triple chimera***

| Element ID | Accession number | Coordinates | 5' - part | Middle part | 3' - part | Strand | Direct  repeats |
| --- | --- | --- | --- | --- | --- | --- | --- |
| 33 | AACU02000185 | 6882-12722 | WEIRD (1113nt) | MgSINE (349nt) | MGL (4379nt) | - | + |

- * the end of the contig is within the WEIRD sequence

# Please find the full chimera sequences below ↓

| 1 | AACU02000185 |
| --- | --- |

139681 tttcccttta atatcgccaa acctgcattc gccatttgtt gacttaaaca agtatattcc

139741 aatcgcccta ctgccgttta tttatcatgg ttttctactc ttagtattac tcggggctgt

139801 tcgttgtggg tccccttaga cacgggcccc aacaatgaag actttcgatt ttaactgcgt

139861 gtgttgaaca accacgtttt taatatttat ttggaattgg ttacattaag tatcgcatac

139921 actaccattc atgtaccgga tggtatacta ttgaagaaat cgtggaaatt aatataaata

139981 gaagaaaacc aaagcattaa caaattaatt caccgaaatg gcccacaata ccaatagcat

140041 tgtttaatct ataaatattt aatgccatga ttgttattta taaaaggcat aatttttcta

140101 aaaaaaaaaa gaaacttgta cgtaaataat ggcgtaccaa tatttgatgt aaccaagttt

140161 ttttaataca tattgaaacc gtggttgttg agatccagat aatcaaaact cacaatgtta

140221 cac**aagttgt** **gcatttt**caa ctctctcccg tacacaaaat tttcagtatg ggcgcacgga

140281 ccatgctgtt aacgtttgcg cgggacttcc gcctgtagcc ctaacaggcg cgcacacacg

140341 aaaaactctt caacctgtcg ccctaagcac cgtcactaca cggaacgcct ttcctcgcaa

140401 cacccatgta taatcgacag atctcccctt tgacgagttt tccactgatc gcggtgaagg

140461 aagagtccga tcggcggctc ggcgcgccac ccgcatagag gcgcacaaga caaggcccaa

140521 tctcgcgatg cgagccagcg tggcacaaaa aactttgcca gccggaaagg caggcgtcct

140581 tccccggaag gctagacaat acggtttaaa agaaagggaa attaggctga gaaccacccg

140641 acggttcttc gaatcgacag ggcaccctct caccccctca caccttctgg gattgcctgc

140701 agcgaggagg tagtgccgtt acgggggttg gttagttgta cgtggaccgg aagcactgtg

140761 ccggcgcggc agtctccaaa gggtcccgac agcaagacgt ggtcggccca ttgctggaga

140821 cggctttggg taacgggaag caagaaacgt gggcacggcc ttggttcgca cccttggtac

140881 accccgtgga cggtgggccg tggtggaata gtgtgactga ggcgccccat gcttctcact

140941 cgcactggga atctgcgccg ggtctggagg acgggaagaa ctcgattgca ttcatgtcca

141001 cattgtggcg ctgggctagc ccaggaacca acgggggact aagctgtgga tggacatgta

141061 cggggtgtcg ccggcttttc gaagctttct actcgcacta gggacgtaca ccggggcaga

141121 tgatgtcgtc ggcatgttag gcggaagaga cgctatttta gccggagagg acggatgcgc

141181 gcgtcggtct accaagaggc ctttttgtgg atgcggtagg cttaaggtgc aaaccttgta

141241 tttacgtggc aggctgggta tccagcgact aggagggctc tagctggcct gtccgttaga

141301 gggtctggtc caaccctgtg actatagttt actgtaggaa atgcaagg(WEIRD 1111bp)ag acgaaccctc

141361 cgtgcgcctg aaggcacggg gtcgcgtcag tgaacaaacc tgtaagcagg accgggcccg

141421 aacccggtca ggcacgatcc gcctctgccc tccttgtttt ccccctgtgt aaataaagaa

141481 gatagaacgc gcgccgagat acccctcggg aggttgctaa cggccggcta acaagccggg

141541 ccgagcccgg cgttaactaa tactactact actactac(MGL, 5748-5977, str+)ta ctact**aagtt** **gtgcatttt**c

141601 ctagataaaa cttaatcata gtccaagtta atcaggtgaa caagagggga ccacaacgaa

141661 cagcccacga gtaacatgtc acgtttgaga ttatcatttt tggtggaata agttggttcg

141721 ccgctaaaca cgagcatatt caagtcactt ctccgttgta tatcaacgcc ttcaggcctt

141781 cagtatgttt agggcggacg tgcttggggc tacgctatat tacaaatgca ttgaaatacc

141841 acttacggga aaaagaaggt ttcgcattga ggtgcaaaag ttgattgtgt ttaaccgcga

141901 agacgttttg attgatctta cgaggagggc tgaaaagtga aaaggggcag aacaatcacc

141961 ttgtgtaatg ccgaggtacc tgttgctgaa atggctccga gttgatgacg tgattaatta

142021 gaactatcta gaggaaatct aatcaaacgc aaacacgcat ccagagctaa aaagtatcag

142081 catagcgccg tatggccgcc accataacct cgagcaaatc ttgccgagtc tccaggtcga

142141 cgccgaccga cagccggaga aaggggtcag agtcctttgc catggacgac gcggacgaga

142201 tcctggtggt ggaaaaccca aagctcacgc ccttggtgac ggccagaccc ttggccttgg

142261 cgccggcgag caccaagtct atacaggctt cgagcccgtc cttgttgttc agcccgtcgc

| 2 | AACU02000635 |
| --- | --- |

55441 ccctgaggca ccgcgcttat ggttatagta aaacatcgga tgatcccgat tatatatatg

55501 gtattgtctg taaactggat ccgtgaatgc atctgccgaa actttcttat tcattgatta

55561 gattcgcccc tcgaaaagct tgtatgttgt acgaagccgg tttcttataa aaagggggcg

55621 taactcaata ggaatgtcca accacatttt gcacccacac cccgcaaatt acacggtggt

55681 atcggtagct acttgtgggc acccgacaaa agtttggttt attattttcc aacaggcgat

55741 acgttaatga acttgttgat aggaacaggg gtgctcatag cccccaatcc atacctttgt

55801 aaaatcgaat ccccaaatat acgtatccct tcgaacccct ggtacaccaa gaccccaagg

55861 tttacattct tttgagtcac gtatgcataa ctttcatcaa tcccataact tggtttcgat

55921 gtggaacttg gctttgttcg acattgcaat gactgcgcgc gcacccttgc ttatacgttg

55981 ttacttgtgt gtgtttttct gtttgtttct tggttaaaga gtagtagtag tagtagtagt

56041 attagttaac gccgggctcg gcccggcttg ttagccggcc gttagcaacc tcccgagggg

56101 tatctcggcg cgcgttctat cttctttatt tacacagggg gaaaacaagg agggcagagg

56161 cgtgatcgtg cctgaccggg ttcgggcccg gtcctgctta gggggttagt tcactgacgc

56221 gaccccgtgc ctaaggtgca cggagggttc gtctgacggc ttgtgccgtg aagtgtgggt

56281 gaaaa(MGL, str-, 5717-5977)ccttg catttcctac agtaaactat agtcacaggg ttggaccaga ccctctaacg

56341 gacaggccag ctagagccct cctagtcgct ggatacccag cctgccacgt aaatacaagg

56401 tttgcacctt aagcctaccg catccacaaa aaggcctctt ggtagaccga cgcgcgcatc

56461 cgtcctctcc ggctaaaata gcgtctcttc cgcctaacat gccgacgaca tcatctgccc

56521 cggtgtacgt ccctagtgcg agtagaaagc ttcgaaaagc cggcgacacc ccgtacatgt

56581 ccatccacag cttagtcccc cgttggttcc tgggctagcc cagcgccaca atgtggacat

56641 gaatgcaatc gagttcttcc cgtcctccag acccggcgca gattcccagt gcgagtgaga

56701 agcatggggc gcctcagtca cactattcca ccacggccca ccggtccacg gggtggtacc

56761 aagggtgcga accaaggccg tgcccacgtt tcttgcttcc cgttacccaa agccgtctcc

56821 agcaatgggc cgaccacgtc ttgctgtcgg gaccctttgg agactgccgc gccggcacag

56881 tgcttccggt ccacgtacaa ctaaccaacc cccgtaacgg cactacctcc tcgctgcagg

56941 caatcccaga aggtgtgagg gggtgagagg gtgccctgtc gattcgaaga accgtcgggt

57001 ggttctcagc ctaatttccc tttcttttaa accgtattgt ctagccttcc ggggaaggac

57061 gcctgccttt ccggctggca aagttttttg tgccacgctg gctcgcatcg cgagattggg

57121 ccttgtcttg tgcgcctcta tgcgggtggc gcgccgaacc gccgatcgga ctcttccttc

57181 accgcgatca gtggaaaact cgtcaaaggg gagatctgtc gattatacat gggtgttgcg

57241 aggaaaggcg ttccgtgtag tgacggtgct tagggcgaca ggttgaagag tttttcgtgt

57301 gtgcgcgcct gttagggcta caggcggaag tcccgcgcaa acgttaacag catggtccgt

57361 gcgcccatac tgaaaatttt gtgtacggga gagagttg(WEIRD, str-)tt ggttaacgag tacatctacg

57421 agtttaacaa accttcagga aaggttaaat ggcctttgtt ggaggtttta aattcccgat

57481 ataacgtgag agccgaataa aatcaactgc ctggtttcca ttccgggctt gcaagcgttc

57541 gtcccaattc gcaaagcaaa aagaaagaat gctcctcgga ccagagaaac ccgcgctccc

57601 cgagctcgac gaccagctgc ggcgctctgc ctgtgaccgc tgccggtgcc agaaactgcg

57661 ctgcgagcgg cctagcggcc cgccggatcc cagctccgag accgcaacta gagatggcag

57721 cgacggcggc aggtcctcga ctggcgtccc gttggtgtcc tgcctccgct gccagcgggt

57781 tggcgccatt tgcaccacca gcttccagca gcgaccgggc cgtccgcggc tgttggacag

57841 ctcgtcattg cgtggggctg ggacacgccg ggcgcgcgcg aaacaggtgg cggcgcaggc

57901 tgaccgggcc gactcctccc atcacggccg gcggcagtgt caacagcgga gtaatgcggc

57961 cgctgctgct tgtccgagcc ggcacgatgg cctccgtgct gaagaggagc catgccaggc

58021 ggcccaggta cagcaggtta catcgccacg accagctgat ggacagcaaa gccatcctag

58081 tggcttcagc agccccatcg atccttttcc ttctccgcac cctcgcacct ccgaggggag

58141 caggaccact ccgtctgatg aggaccattc gggcttctgg ggctcgtgtc ttcagtgcat

58201 gtccccttcg tgggatttgg gcggggagga ctttcccaac ctgttcacga cggacatgga

58261 aatactgagg atggacggta ccgacgtacc tgctcccgcc agcaatgccg atgccaccgt

| 3 | AACU02000308 |
| --- | --- |

9361 tttcgtgaca ttgggattct tttataagag ttgacatttt gtgttgcggt aaagaacggg

9421 tgtttaagtt ggaaatggtg tggtgcagaa aaagtgcgcg actcggtggt atgcgcgact

9481 cggtgatgaa ccacgttaca aattaattta tggaaattca tttgcggcac cggaaaccct

9541 tctggcgtgc cgacccccac ttcaatgtcg gagggtatat aagaggacag agcaaagtcc

9601 tcccaccaat acattaccgt caaattgcgt acaacgttcc attttctagt gcatctttta

9661 gcgcttttag cacctgttag caaccacgcc gcacctagta gtagtagtag tagtattagt

9721 taacgccggg ctcggcccgg cttgttagcc ggccgttagc aacctcccga ggggtatctc

9781 ggcgcgcgtt ctatcttctt tatttacaca gggggaaaac aaggagggca gaggcggatc

9841 gtgcctgacc gggttcgggc ccggtcctgc ttagggggtt agttcactga cgcgaccccg

9901 tgcctaaggt gcacggaggg ttcgtctgac ggcttgtgcc gtgaagtgtg ggtgaaaagg

9961 cagtaagtct attcctcgtc agagttcgag tcgtttacga tcggtgtccc taggcgtaaa

10021 gtgcgttcgt ggcgcgcggg gcgctgg(MGL, 5615-5977, str-)cgg gccgctctgg ccttgcattt cctacagtaa

10081 actatagtca cagggttgga ccagaccctc taacggacag gccagctaga gccctcctag

10141 tcgctggata cccagcctgc cacgtaaata caaggtttgc accttaagcc taccgcatcc

10201 acaaaaaggc ctcttggtag accgacgcgc gcatccgtcc tctccggcta aaatagcgtc

10261 tcttccgcct aacatgccga cgacatcatc tgccccggtg tacgtcccta gtgcgagtag

10321 aaagcttcga aaagccggcg acaccccgta catgtccatc cacagcttag tcccccgttg

10381 gttcctgggc tagcccagcg ccacaatgtg gacatgaatg caatcgagtt cttcccgtcc

10441 tccagacccg gcgcagattc ccagtgcgag tgagaagcat ggggcgcctc agtcacacta

10501 ttccaccacg gcccaccgtc cacggggtgt accaagggtg cgaaccaagg ccgtgcccac

10561 gtttcttgct tcccgttacc caaagccgtc tccagcaatg ggccgaccac gtcttgctgt

10621 cgggaccctt tggagactgc cgcgccggca cagtgcttcc ggtccacgta caactaacca

10681 acccccgtaa cggcactacc tcctcgctgc aggcaatccc agaaggtgtg agggggtgag

10741 agggtgccct gtcgattcga agaaccgtcg ggtggttctc agcctaattt ccctttcttt

10801 taaaccgtat tgtctagcct tccggggaag gacgcctgcc tttccggctg gcaaagtttt

10861 tttttttttt tttttgtgcc acgctggctc gcatcgcgag attgggcctt gtcttgtgcg

10921 cctctatgcg ggtggcgcgc cgagccgccg atcggactct tccttcaccg cgatcagtgg

10981 aaaactcgtc aaaggggaga tctgtcgatt atacatgggt gttgcgagga aaggcgttcc

11041 gtgtagtgac ggtgcttagg gcgacaggtt gaagagtttt tcgtgtgtgc gcgcctgtta

11101 gggctacagg cggaagtccc gcgcaaacgt taacagcatg gtccgtgcgc ccatactgaa

11161 aattttgtgt acgggagaga gttg(WEIRD, str-)tcctct gattacgagt cagctttacg gggacaaagc

11221 ccagacctgt ttaggcgctg agagtggcgt gcattacagt ttccgggagc aatgacggag

11281 ggggcccttg tacaatacgg gtgttgaaat gcatgcccgg ttggacagat aaaggagaaa

11341 tcaataagta ttaaattctc cactactcag ctctggcttc agtaacaatt gcacaaatat

11401 gttccgagtt tattcctacg taaacagtaa tgacctcgca agtatcgcca ggtttgccaa

11461 gtgagatcag gtagcggcca gcgaaaataa gaattcgaga ccatacacgc tacttatttt

11521 gataatgtat actccttcct aacgctgacc tctaaaaaat gacaacagac gtggtaagac

11581 gtatgttcct caagaaagag ttatcactac tataacaatt gacaaataga attgttagcc

11641 ttgtacacta cagccaaaca tttgcttgag gaacctgccc gtgtatgcgc cactcccctt

11701 gcatagccag acgcgagaca ggtcaggaat tgagatggca aatatgtctt gtacccaagt

11761 ggctgtgtcg cacttgtgat ttggcagtcg attccacgtc tcctcgactg ttactgtcgt

11821 cactgggagc tgtttgtcac gagacaacgc cttaataaat ctcgaccttt cccatgatca

11881 tggatggcta caaggtccca aaagctttgg gcttggaggg aatagccagc tgaaccgtgg

11941 tattgctcca agtctagcat tggcgtagat attagcagaa gaaggtcaag aaacccacca

12001 ggtaatccag gaccatgaga agaatcatga aaggcaagca gccagcctgc gccgaacctg

| 4 | AACU02000436 |
| --- | --- |

31861 tgccctcaag ctcattggac tggaagacag agaaatagtg cccttttgga tgagatctgg

31921 cttaatggga agcgtttcat accccgcctt tctcaccagc aaaaccgtag ctcgacttat

31981 ccacacatgc aatgtttaaa ctgctgcatc ccacgggtcc aacggaaacc atgagacgaa

32041 aagcaacact gacagcagtc aaaattctgc ccaacagagc tggaatggcg tgtctgatct

32101 catgtcgtgg caagacccac gcggttatta tctccccggg cgatgctaaa atccgcgccc

32161 tacatgggga aagtcaatgt ttcccctcac tcttttccaa acgtggtaat taatccaaaa

32221 gcaaaacatg tgaaccccgc ttgtaggtcc ctcgggtccc tcgatcctgt caacgcaaga

32281 agccagagcg tcctgcatcg cgtaccaaca caaggcggct taaaggtttc gtcggacaca

32341 tgctagacct tcaaccgccg tagcgcaacg tgcctgtgat aaagcgattt gaaagccttg

32401 cctggctgtt gaacggaatt gcacagggca aaatgctcaa atgtctatca tgcacacaac

32461 gcttcacagt agatgagtga aagtaatcca aggaagtcga c**gctgctcag** **gcaa**gtagta

32521 gtagtagtag tagtagtagt attatttaac gccgggctcg gcccggcttg ttagccggcc

32581 gttagcaacc tcccgagggg tatctcggcg cgcgttctat tttctcttat ttacacaggg

32641 ggaaaacaag gagggcagag gcggatcgtg cctgaccggg ttcgggcccg gtcctgctta

32701 gggggatagt tcactgacgc gaccccgtgc ctaaggtgca cggagggttc gtctgacggc

32761 ttgtgccgtg aagtgtgggt gaaaaggcag taagtctatt cctcgtctga gttcgagtcg

32821 tttacgatcg gtgtccctag gcgtaaagtg cgttcgtggc gcgcggggcg ctggcgggcc

32881 gctctggggc gggcgctgaa atagttggtg gctatcgaaa acgcctgaaa gcttgtcggt

32941 tgcccgaggc ttgtctggaa gaatttgcgg cgttgggcgc ggtctggcgg cccgaccggc

33001 cggtcgttgt caggccacgg ccagtttctc cacaccgccc gcgaaaagcg gcagtgcacc

33061 gggtgttcag gggaggtccg cttccagcac caggcacacg aggtgtttgc atcctggtgg

33121 ttgaaacggt catggtaggc tttgaaatcg ccgtggccgg tcctcatggc caaataatgg

33181 cccagtaggg gttttggcaa acgcagttcc tcgggctcct ttctcggtgt atattggaat

33241 ttccattccc tatatgcggg ggaccgttca cagagttttt tacgccacca gtccttcttt

33301 atattcgaaa gaatggtttt gaggaccgtg ccggcaccgc tatacgtggt ttgctgagcc

33361 ctcgggtctg ggtccggcgg tccggcggag ccggccttgg ccagctcgtc agcccggtcg

33421 tttcccggga ttccctggtg cccagggcac caacggaccc gaacctcggt actttatttt

33481 cggagtagat cgacaaggtt atggaactcc agaaaggccc attgggacga acgcggcgcg

33541 tcgcctctaa(MGL, 4952-5977, str-) ccttgcattt cctacagtaa attatagtca cagggttgga ccagaccctc

33601 taacggacag gccagctaga gccctcctag tcgctggata cccagcctgc cacgtaaata

33661 caaggtttgc accttaaacc taccgcatcc acaaaaaggc ctcttggtag accgacgcgc

33721 gcatccgtcc tctccggcta aaatagcgtc tcttccgcct aacatgccga cgacatcatc

33781 tgccccggtg tacgtcccta gtgcgagtag aaagcttcga aaagccggcg acaccccgta

33841 catgtccatc cacagcttag tcccccgttg gttcctgggc tagcccagcg ccacaatgtg

33901 gacatgaatg caatcgagtt cttcccgtcc tccagacccg gcgcagattc ccagtgcgag

33961 tgagaagcat ggggcgcctc agtcacacta ttccaccacg gcccaccgtc cacggggtgt

34021 accaagggtg cgaaccaagg ccgtgcccac gtttcttgct tcccgttacc caaagccgtc

34081 tccagcaatg ggccgaccac gtcttgctgt cgggaccctt tggagactgc cgcgccggca

34141 cagtgcttcc ggtccacgta caactaacca acccccgtaa cggcactacc tcctcgctgc

34201 aggcaatccc agaaggtgtg agggggtgag agggtgccct gtcgattcga agaaccgtcg

34261 ggtggttctc agcctaattt ccctttcttt taaaccgtat tgtctagcct tccggggaag

34321 gacgcctgcc tttccggctg gcaaagtttt ttgtgccacg ctggctcgca tcgcgagatt

34381 gggccttgtc ttgtgcgcct ctatgcgggt ggcgcgccga accgccgatc ggactcttcc

34441 ttcaccgcga tcagtggaaa actcgtcaaa ggggagatct gtcgattata catgggtgtt

34501 gcgaggaaag gcgttccgtg tagtgacggt gcttagggcg acaggttgaa gagtttttcg

34561 tgtgtgcgcg cctgttaggg ctacaggcgg aagtcccgcg caaacgttaa cagcatggtc

34621 cgtgcgccca tactgaaaat tttgtgtacg ggagagagtt g(WEIRD, str-)**gctgctcag** **gcaa**ggagtg

34681 ttgtgagaca cggcagaata agaattagcc cgcccagcgt aatgcttgcg atataagcgc

| 5 | AACU02000380 |
| --- | --- |

47581 gatttaatgg cctggtgtta gggcgtaata aaatccggcc attacagcga aaagagcctg

47641 gaacgcgggg ttggacgagc ataatcgaat gtgtatcagc tacgggggct gttatacctc

47701 ccctcgttat atttaagggg aaaaacgtac agcaacaatg gtttccagct gatttaagtc

47761 ctttcgatac ctggcaattt catgcaaccg aaaacgggtg gacaaataat gaaacaggta

47821 tcgaatggtt aaaaaaggtg tttattccgt atacccaact tttaacccct gaaaagcggt

47881 tattagttat ggatggccat ggatcacata taacggacga atttatgctt ctttgcttgc

47941 aaaacaatat tcaactcc**ta tatttacccc ct**caactctc tcccgtacac aaaattttca

48001 gtatgggcgc acggaccatg ctgttaacgt ttgcgcggga cttccgcctg tagccctaac

48061 aggcgcgcac acacgaaaaa ctcttcaacc tgtcgcccta agcaccgtca ctacacggaa

48121 cgcctttcct cgcaacaccc atgtataatc gacagatttc ccctttgacg agttttccac

48181 tgatcgcggt gaaggaagag tccgatcggc ggttcggcgc gccacccgca tagaggcgca

48241 caagacaagg cccaatctcg cgatgcgagc cagcgtggca caaaaaactt tgccagccgg

48301 aaaggcaggc gtccttcccc ggaaggctag acaatacggt ttaaaagaaa gggaaattag

48361 gctgagaacc acccgacggt tcttcgaatc gacagggcac cctctcaccc cctcacacct

48421 tctgggattg cctgcagcga ggaggtagtg ccgttacggg ggttggttag ttgtacgtgg

48481 accggaagca ctgtgccggc gcggcagtct ccaaagggtc ccgacagcaa gacgtggtcg

48541 gcccattgct ggagacggct ttgggtaacg ggaagcaaga aacgtgggca cggccttggt

48601 tcgcaccctt ggtacacccc gtggacggtg ggccgtggtg gaatagtgtg actgaggcgc

48661 cccatgcttc tcactcgcac tgggaatctg cgccgggtct ggaggacggg aagaactcga

48721 ttgcattcat gtccacattg tggcgctggg ctagcccagg aaccaacggg ggactaagct

48781 gtggatggac atgtacgggg tgtcgccggc ttttcgaagc tttctactcg cactagggac

48841 gtacaccggg gcagatgatg tcgtcggcat gttaggcgga agagacgcta ttttagccgg

48901 agaggacgga tgcgcgcgtc ggtctaccaa gaggcctttt tgtggatgcg gtaggtttaa

48961 ggtgcaaact ttgtatttac gtggcaggct gggtatccag cgactaggag ggctctagct

49021 ggcctgtccg ttagagggtc tggtccaacc ctgtgactat agtttactgt aggaaatgca

49081 agg(WEIRD, str+)accaact acttcagcgc ccgccccaga gcggcccgcc agcgccccgc gcgccacgaa

49141 cgcactttac gcctagggac actgatcgta aacgactcga actctgacga ggaatagact

49201 tactgccttt tcacccacac ttcacggcac aagccgtcag acgaaccctc cgtgcacctt

49261 aggcacgggg tcgcgtcagt gaactaaccc cctaagcagg accgggcccg aacccggtca

49321 ggcacgatcc gcctctgccc tccttgtttt ccccctgtgt aaataaagaa gatagaacgc

49381 gcgccgagat acccctcggg aggttgctaa cggccggcta acaagccggg ccgagcccgg

49441 cgttaactaa tactactact actactac(MGL, 49312-49450, str+)ta ctactactac tactac**tata** **tttaccccct**

49501 cattcgtcac acgttcttca accgttggat ttatcggttt ttgggccgtt aaaggaagct

49561 tatcgacgtc acctgggatt tgtaaaccag ttttgctgtt caacggttgt tgggaaacga

49621 aactttctac tttgctatcg aaaagccaga tcaaaagcat ttatagcaaa aaccattcaa

49681 tctggtgggc gtacgacggg gttatggccg gtgaacttgg caaaaccact tttaaaccct

49741 tttttattag aaaatagcaa cgccaacgtc gaaaaaggta aaaataacgg cttccaaagg

49801 gataaaacac cggaaaatcc aacccaaaaa attaacgacc agtctttact tatttggaaa

49861 acccctaaaa cgacccgaga tattcgactt caactacagg aaatttcccg gtccgaaaaa

| 6 | AACU02000714 |
| --- | --- |

224101 accaaaggcg aggccgccag agacgtcaac tcactggcag gtattacttt ggtgcaatgg

224161 tgcttaacaa aggcgttttg cgccacatgc atagttggga tctcgcctgg ctgttgtggc

224221 ttcaaggcta tctacttgat tatttgagtg attaatagat tgaggacatg taaggcggat

224281 tgagttactg aatgggcaaa gatgagctgg gctggagatg ctcatcaagg agtgaaaggg

224341 cctggaaccc attgtatttt ctacaccgct cctatttttg aaagcgataa caataacaaa

224401 aagcacaact ttatccacgg tacagctctt accattatcg ttcaactctc tcccgtacac

224461 aaaattttca gtatgggcgc acggaccatg ctgttaacgt ttgcgcggga cttccgcctg

224521 tagccctaac aggcgcgcac acacgaaaaa ctcttcaacc tgtcgcccta agcaccgtca

224581 ctacacggaa cgcctttcct cgcaacaccc ctgtataatc gacagatctc ccctttgacg

224641 agttttccac tgatcgcggt gaaggaagag tccgatcggc ggctcggcgc gccacccgca

224701 tagaggcgca caagacaagg cccaatctcg cgatgcgagc cagcgtggca caaaaaactt

224761 tgccagccgg aaaggcaggc gtccttcccc ggaaggctag acaatacggt ttaaaagaaa

224821 gggaaattag gctgagaacc acccgacggt tcttcgaatc gacagggcac cctctcaccc

224881 ccttacacct tctgggattg cctgcagcga ggaggtagtg ccgttacggg ggttggttag

224941 ttgtacgtgg accggaagca ctgtgccggc gcggcagtct ccaaagggtc ccgacagcaa

225001 gacgtggtcg gcccattgct ggagacggct ttgggtaacg ggaagcaaga aacgtgggca

225061 cggccttggt tcgcaccctt ggtacacccc gtggacggtg ggccgtggtg gaatagtgtg

225121 actgaggcgc cccatgcttc tcactcgcac tgggaatctg cgccgggtct ggaggacggg

225181 aagaactcga ttgcattcat gtccacattg tggcgctggg ctagcccagg aaccaacggg

225241 ggactaagct gtggatggac atgtacgggg tgtcgccggc ttttcgaagc tttctactcg

225301 cactagggac gtacaccggg gcagatgatg tcgtcggcat gttaggcgga agagacgcta

225361 ttttagccgg agaggacgga tgcgcgcgtc ggtttaccaa gaggcctttt tgtggatgcg

225421 gtaggcttaa ggtgcaaacc ttgtatttac gtggcaggct gggtatccag cgactaggag

225481 ggctctagct ggcctgtccg ttagagggtc tggtccaacc ctgtgactat agtttactgt

225541 aggaaatgca agg(WEIRD, str+)accaact acttcagcgc ccgccccaga gcggcccgcc agcgccccgc

225601 gcgccacgaa cgcactttac gcctaggggc accgatcgta aacgactcga actctgacga

225661 ggaatagact tactgccttt tcacccacac ttcacggcac aagccgtcag acgaaccctc

225721 cgtgcacctt aggcacgggg tcgcgtcagt gaactaaccc cctaagcagg accgggcccg

225781 aacccggtca ggcacgatcc gcctctgccc tccttgtttt ccccctgtgt aaataaagaa

225841 gatagaacgc gcgccgagat acccctcggg aggttgctaa cggccggcta acaagccggg

225901 ccgagcccgg cgttaaataa tactactact actactac(MGL, str+, 5593-3977)ta ctactaccat tatcgttccc

225961 gcaattcatg cgcggctgtt ggtgcctttg ctctgtgaca aacggctcac cccaccagga

226021 tgcctcagag ctggactagt agcttgttga aatcaaacaa aatctacccc tcctagactc

226081 aagactaagg atgctgcgcc ataacagcac gattaggtgc tgacgtacca ttgtacacgc

226141 ccttgattga acaccaagac ttacacaaaa gtgttggata ccaacatgcc caagagtcca

226201 atccgccaaa aatctggtca tttgcgtcct ggccgctctg tgggccctcc acgtaccatc

226261 aactacacgc tggaactacg tggagcaagt attcctgcgc cgcatcccga gaccaccagg

| 7 | AACU02000527 |
| --- | --- |

1921 gtttgatgct caaccttacg tcatatcctt acctatccat acaaaaaaac tcttgggaca

1981 tgactgacct ggttcggccc ctcacacagg tcgcagctca tgcccgatcc tcaactccct

2041 cgccaaccat ggattcctcc ctcgcgacgg cctcaatatt tcgcgcgagc aggtgctaga

2101 cgcgatgcaa aagggcctgg gcttcaacac gaccggcccg ctcgagtcga ccactgccca

2161 cgggctcacc atgtcgagca ctggggacaa caatacgatg cacctcgacg acattgatag

2221 gcacaatggt aatagaacat gatgccacca gagaatcggg caaatgaatt agcttgatcg

2281 attgctgatt ccctgttgtg catattctca ttctagtcat cgagcacgac gccagcctga

2341 gtcgcgccga cctcggtgtg ggcgacccaa gacccttcaa cccaaccatc tgggccactt

2401 cgcttgtgtt cttctcaact ctctcccgta cacaaaattt tcagtatggg cgcacggacc

2461 atgctgttaa cgtttgcgcg ggacttccgc ctgtagccct aacaggcgcg cacacacgaa

2521 aaactcttca acctgtcgcc ctaagcaccg tcactacacg gaacgccttt cctcgcaaca

2581 cccatgtata atcgacagat ctcccctttg acgagttttc cactgatcgc ggtgaaggaa

2641 gagtccgatc ggcggctcgg cgcgccaccc gcatagaggc gcacaagaca aggcccaatc

2701 tcgcgatgcg agccagcgtg gcacaaaaaa aaactttgcc agccggaaag gcaggcgtcc

2761 ttccccggaa ggctagacaa tacggtttaa aagaaaggga aattaggctg agaaccaccc

2821 gacggttctt cgaatcgaca gggcaccctc tcaccccctc acaccttctg ggattgcctg

2881 cagcgaggag gtagtgccgt tacgggggtt ggttagttgt acgtggaccg gaagcactgt

2941 gccggcgcgg cagtctccaa agggtcccga cagcaagacg tggtcggccc attgctggag

3001 acggctttgg gtaacgggaa gcaagaaacg tgggcacggc cttggttcgc acccttggta

3061 caccccgtgg acggtgggcc gtggtggaat agtgtgactg aggcgcccca tgcttctcac

3121 tcgcactggg aatctgcgcc gggtctggag gacgggaaga actcgattgc attcatgtcc

3181 acattgtggc gctgggctag cccaggaacc aacgggggac taagctgtgg atggacatgt

3241 acggggtgtc gccggctttt cgaagctttc tactcgcact agggacgtac accggggcag

3301 atgatgtcgt cggcatgtta ggcggaagag acgctatttt agccggagag gacggatgcg

3361 cgcgtcggtt taccaagagg cctttttgtg gatgcggtag gcttaaggtg caaaccttgt

3421 atttacgtgg caggctgggt atccagcgac taggagggct ctagctggcc tgtccgttag

3481 agggtctggt ccaaccctgt gactatagtt tactgtagga aatgcaagg(WEIRD, str+)t cgggcaaccg

3541 aaaagctttg aggcgttttc gatagccacc aactacttca gcgcccgccc cagagcggcc

3601 cgccagcgcc ccgcgcgcca cgaacgcact ttacgcctag ggacaccgat cgtaaacgac

3661 tcgaactctg acgaggaata gacttactgc cttttcaccc acacttcacg gcacaagccg

3721 tcagacgaac cctccgtgca ccttaaggca cggggtcgcg tcagtgaact aaccccctaa

3781 gcaggaccgg gcccgaaccc ggtcaggcac gatccgcctc tgccctcctt gttttccccc

3841 tgtgtaaata aagaagatag aacgcgcgcc gagatacccc ctcgggaggt tgctaacggc

3901 cggctaacaa gccgggccga gcccggcgtt aaataatact actactacta ctac(MGL, str+, 5555-5977)tactac

3961 ttcgcttgtg ttctggccgg accaggccat caacatcaat caaatggctc gcgcctttgc

4021 tcaacggatg acggcggccg cggcgtccaa cccgagattc tcgctgagcg aaacccagga

4081 ggccgtcgcc atcaacgcac tcagcaacgt gatgctcatg tttggcgacg gtaccgtcaa

4141 caccacggcc aacaagctgt gggtgagggt gctatttggt gagcaggagc tctttttctg

4201 tctcaacagt cttgcccacg ggcgcagcac agacgagaaa atgtctgttg ggatcctcca

4261 ctgactctcc ttgttgctac gatataaaga gcaagaacgt ctcccgtttg ctgagggatg

4321 gcgccagccg gagcagccac tccagcctgc agtaacagcc gagctcaaca aaagtttcaa

4381 ggcggcaatg cccgagcagc gcctggggtg tcccgctact ccaccaagca tgccagtgtc

4441 agctcccccg tagctgcgct ggcgacagca ccggcgcctg ccccagttgc tgccccagca

4501 ttccagaagg gagcggcata ggcagagatc acgactctga atgcccaaag agcagccaca

| 8 | AACU02000343 |
| --- | --- |

27781 aggcatagct tagtgttgta cgaggcaaaa tttgacggca aacccagaca gtttggactt

27841 tggcatatct accgtctttt actcgcttgt cggattccaa gcaaagctca gctcgcaaag

27901 ccaggggcac aaaaaaaaaa accttgttgt cggccaaggt gagaaaagac taacccagcc

27961 ctgccgagca cgcttcatgc gccgcccctc aaggctgata gagaagaagc agtatgtggc

28021 atttcaaaat tgagatgacc tgaccacaga tcgtcgccac agctcgccac aatgtacaaa

28081 gttgcagctg ggctgcggcc tgtctttgag gcgtgcgacc gaacctttcg aatgtgtacg

28141 atctt**ctctg** **caccccta**gt agtagtagta gtagtattag ttaacgccgg gctcggcccg

28201 gcttgttagc cggccgttag caacctcccg aggggtatct cggcgcgcgt tctatcttct

28261 ttatttacac agggggaaaa caaggagggc agaggcggat cgtgcctgac cgggttcggg

28321 cccggtcctg cttagggggt tagttcactg acgcgacccc gtgcctaagg tgcacggagg

28381 gttcgtctga cggcttgtgc cgtgaagtgt gggtgaaaag gcagtaagtc tattcctcgt

28441 cagagttcga gtcgtttacg atcggtgtcc ctaggcgtaa agtgcgttcg tggcgcgcgg

28501 ggcgctggcg ggccgctctg gggcgggcgc tgaagtagtt ggtggctatc gaaaacgcct

28561 caaagctttt cggttgcccg aagcttgtct ggaagaattt ccagcgttgg gcgcgatttg

28621 gcggcccggc cggccggtcg ttattaggcc acggccagtt tctccacacc gcccgcgaat

28681 agcggcagtg caccgggtgc tcaggggagg tccgcttcca gcaccaggca cacgaggtgt

28741 ttgcatcctg gtggttgaaa cggtcatggt aggccttgaa atcgccgtgg ccggtcctca

28801 t(MGL, 5335-5935, str -)ccttgcatt tcctacagta aactatagtc acagggttgg accagaccct ctaacggaca

28861 ggccagctag agccctccta gtcgctggat acccagcctg ccacgtaaat acaaggtttg

28921 caccttaagc ctaccgcatc cacaaaaagg cctcttggta gaccgacgcg cgcatccgtc

28981 ctctccggct aaaatagcgt ctcttccgcc taacatgccg acgacatcat ctgccccggt

29041 gtacgtccct agtgcgagta gaaagcttcg aaaagccggc gacaccccgt acatgtccat

29101 ccacagctta gtcccccgtt ggttcctggg ctagcccagc gccacaatgt ggacatgaat

29161 gcaatcgagt tcttcccgtc ctccagaccc ggcgcagatt cccagtgcga gtgagaagca

29221 tggggcgcct cagtcacact attccaccac ggcccaccgt ccacggggtg taccaagggt

29281 gcgaaccaag gccgtgccca cgtttcttgc ttcccgttac ccaaagccgt ctccagcaat

29341 gggccgacca cgtcttgctg tcgggaccct ttggagactg ccgcgccggc acagtgcttc

29401 cggtccacgt acaactaacc aacccccgta acggcactac ctcctcgctg caggcaatcc

29461 cagaaggtgt gagggggtga gagggtgccc tgtcgattcg aagaaccgtc gggtggttct

29521 cagcctaatt tccctttctt ttaaaccgta ttgtctagcc ttccggggaa ggacgcctgc

29581 ctttccggct ggcaaagttt tttttgtgcg cacgctggct cgcatcgcga gattggccct

29641 tgtcttgtgc gcctctatgc gggtggcgcg ccgaaccgcc gatcggactc ttccttcacc

29701 gcgatcagtg gaaaactcgt caaaggggag atctgtcgat tatacatggg tgttgcgagg

29761 aaaggcgttc cgtgtagtga cggtgcttag ggcgacaggt tgaagagttt ttcgtgtgtg

29821 cgcgcctgtt agggctacag gcggaagtcc cgcgcaaacg ttaacagcat ggtccgtgcg

29881 cccatactga aaattttgtg tacgggagag agttg(WEIRD, str-)**ctctg** **caccccta**ag cgatccatta

29941 ttttgattac tttgtacttt gcccatcaac cgcatggcct gatgtctgcg cagcctaaga

30001 ccagattgat gcgccaaaaa aacaaagatg atgttgcctg ggggaaaaaa cgaggatgcg

30061 acggatgcgt gggtagccag catcttgggc aaacattaga gagccatcgg ggagttgaga

| 9 | AACU02000467 |
| --- | --- |

2641 gcgagatcga gagcctgctc gggtcaaatg atgagcaagt agttgttatt ctcttcaaca

2701 acggccaagg gaacaacaac tcgccgagat taataggctt tcacgccgcc aacagtccag

2761 ctcgagacca caccgtggat ggggcctgtg aagtcaaaga agacaccagc cctatagggc

2821 aagctcgcac ccaaatgctc caagcacgag cccgcgataa acttgcgccg ttcatagtgc

2881 ccaacatgct catcgccatc acagctatgc cgctaaattc caacggcaag gttaaccaat

2941 caaagctcaa gtctttctat gcagcacgcc aaggggccag tcccttgtcg gtcaatcttc

3001 ctagtccttc taccatcgag gcgcacaaac ctggttcgaa ggatttggaa tatcccaccc

3061 cgccgaatga tgctctagcg gactgcctcg tgcactttcg gtcacgtaaa agctctgggc

3121 caacccagca aagaaccatc tatggactct tcgccgtcac aggcctatca aggcaatatc

3181 gcgccatcgc tgagagacta actccggact tcaaccttgt tggcgtcgac aacatattcc

3241 gtgaccagcc aaagcactac ccctccatcg ccggagcaat ggctgccgac catgcagccg

3301 ccattcttca tcacctttac cccgtggacg gtgggccgtg gtggaatagt gtgactgagg

3361 cgccccatgc ttctcactcg cactgggaat ctgcgccggg tctggaggac gggaagaact

3421 cgattgcatt catgtccaca ttgtggcgct gggctagccc aggaaccaac gggggactaa

3481 gctgtggatg gacatgtacg gggtgtcgcc ggcttttcga agctttctac tcgcactagg

3541 gacgtacacc ggggcagatg atgtcgtcgg catgttaggc ggaagagacg ctattttagc

3601 cggagaggac ggatgcgcgc gtcggtctac caagaggcct ttttgtggat gcggtaggct

3661 taaggtgcaa accttgtatt tacgtggcag gctgggtatc cagcgactag gagggctcta

3721 gctggcctgt ccgttagagg gtctggtcca accctgtgac tatagtttac tgtaggaaat

3781 gcaagg(WEIRD, str+, 647-1114)atga ggaccggcca cggcgatttc aaagcctacc atgaccgttt caaccaccag

3841 gatgcaaaca cctcgtgtgc ctggtgctgg aagcggacct cccctgaaca cccggtgcac

3901 tgccgctttt cgtgggcggt gtggagaaac tggccgtggc ctgacaacga ccggccggtc

3961 gggccgccag accgcgccca acgccgcaaa ttcttccaga caagcctcgg gcaaccgaca

4021 agctttcagg cgttttcgat agccaccaac tacttcagcg cccgccccag agcggcccgc

4081 cagcgccccg cgcgccacga acgcacttta cgcctaggga caccgatcgt aaacgactcg

4141 aactctgacg aggaatagac ttactgcctt ttcacccaca cttcacggca caagccgtca

4201 gacgaaccct ccgtgcacct taggcacggg gtcgcgtcag tgaactaacc ccctaagcag

4261 gaccgggccc gaacccggtc aggcacgatc cgcctctgcc ctccttgttt tccccctgtg

4321 taaataaaga agatagaacg cgcgccgaga tacccctcgg gaggttgcta acggccggct

4381 aacaagccgg gccgagcccg gcgttaacta atactactac tactactac(MGL, str+, 5335-5977)t actactactt

4441 cttcatcacc gacgcagttt aggcaaggaa aatgatccgg agcccgtcct gctctttggc

4501 tattcatttt ctgggaccct tgcttgggag attgcgcgag agcttctcgg acgtggccat

4561 cgggtgcaaa tcgtcatggt cgacgccgac gcccgcccgc gccccccgaa agactacgaa

4621 caattcccgg atgaagcttt ggagcggtac ctcgagctgc ccagtgagga aaccatcaag

4681 caaataagag ccctggagat tgatgaggga gacgagatgg ctgtacatcg gaggaatgtc

4741 ctgagccaga gccgccacaa catgcgcctc tgcgacgaat gcgtgccggg tccaatgggc

| 10 | AACU02000098 |
| --- | --- |

661 gcttacatgc gtcgttacat cagcagaaac agccccaaga atcgtctatg gttgagacaa

721 gtcacaagct gcatgctatt gttcaacagt aaatcgtctg ccagtccgga tttggcactc

781 ccagtaacaa ggaagataat ggcaacaacc cgtctgggaa acaaaagggc atcagcaagc

841 aagtcaggag ttgcatgatc agatatttgt ctaataaata tattgcttgg ctctagatct

901 gtaacctggc cttggcgaga atgttgtctt actcatagta agccggccac atcttctaca

961 aaccactaga ctcatcttca ggctattaag caactctctc ccgtacacaa aattttcaat

1021 atgggcgcac ggaccatgct gttaacgttt gcgcgggact tccgcctgta gccctaacag

1081 gcgcgcacac acgaaaaact cttcaacctg tcgccctaag caccgtcact acacggaacg

1141 cctttcctcg caacacccat atataatcga cagatctccc ctttgacgag ttttccactg

1201 atcgcggtga aggaagagtc cgatcggcgg ctcggcgcgc cacccgcata gaggcgcaca

1261 agacaaggcc caatctcgcg atgcgagcca gcgtggcaca aaaaactttg ccagccggaa

1321 aggcaggcgt ccttccccgg aaggctagac aatacggttt aaaagaaagg gaaattaggc

1381 tgagaaccac ccgacggttc ttcgaatcga cagggcaccc tctcaccccc tcacaccttc

1441 tgggattgcc tgcagcgagg aggtagtgcc gttacggggg ttggttagtt gtacgtggac

1501 cggaagcact gtgccggcgc ggcagtctcc aaagggtccc gacagcaaga cgtggtcggc

1561 ccattgctgg agacggcttt gggtaacggg aagcaagaaa cgtgggcacg gccttggttc

1621 gcacccttgg tacaccccgt ggacggtggg ccgtggtgga atagtgtgac tgaggcgccc

1681 catacttctc actcgcactg ggaatctgcg ccgggtctgg aggacgggaa gaactcgatt

1741 gcattcatgt ccacattgtg gcgctggcta gcccaggaac caacggggga ctaagctgtg

1801 gatggacatg tacggggtgt cgccggcttt tcgaagcttt ctactcgcac tagggacgta

1861 caccggggca gatgatgtcg tcggcatgtt aggcggaaga gacgctattt tagccggaga

1921 ggacggatgc gcgcgtcggt ctaccaagag gcctttttgt ggatgcggta ggcttaaggt

1981 gcaaaccttg tatttacgtg gcaggctggg tatccagcga ctaggagggc tctagctggc

2041 ctgtccgtta gagggtctgg tccaaccctg taactatagt ttactgtagg aaatgcaagg(WEIRD, str+)

2101 aagaccccta ctgggccatt atttggccat gaggaccggc cacggcgatt tcaaagccta

2161 ccatgaccgt ttcaaccacc aggatgcaaa cacctcgtgt gcctggtgct ggaagcggac

2221 ctcccctgag cacccggtgc actgccgcta ttcgcgggcg gtgtggagaa actggccgtg

2281 gcttgacaac gaccggccgg tcgggccgcc agaccgcgcc caacgccgca aattcttcca

2341 gacaagcctc gggcaaccga aaagctttga ggcgttttcg atagccacca actacttcag

2401 cgcccgcccc agagcggccc gccagcgccc cgcgcgccac gaacgcactt tacgcctagg

2461 gacaccgatc gtaagcgact cgaactctga cgaggaatag acttactgcc ttttcaccca

2521 cacttcacgg cacaagccgt cagacgaacc ctccgtgcac cttaggcacg gagtcgcgtc

2581 agtgaactaa ccccctaagc aggaccgggc ccgaacccgg tcaggcacga tccgcctctg

2641 ccctccttgt tttccccctg tgtaaataaa gaagatagaa cgcgcgccga gatacccctc

2701 gggaggttgc taacggccgg ctaacaagcc gggccgagcc cggcgttaaa taatactact

2761 actactacta c(MGL, str+, 5307-5977)tactacttc cctacgaaca agtcctttca agctcataca gccacaatgc

2821 gcgctacgtc taccactatt tttgcccttt tgacctccgg cgctattgtc gccggcacgc

2881 agcccaaaaa tgtggcatgc ggcggatgca gtttgaacca aatgtcgaaa caaaatatcg

2941 aaacttgcgc tacacactac gacccaaccg caagttatcc tgtttgggat gaatctgaag

3001 atttttcctg caatatgtgc ccaccgagag agaaattatt gagattaaag gccaagaaaa

| 11 | AACU02000173 |
| --- | --- |

96061 cgcagctgat cctgctgccc tacggctaca gctgcacggc cgtggccgag ggcctggagc

96121 gccagatgga gctggccggc ggcgtcgcgg ccgccatcaa ggccaccaac gggctcgact

96181 ttgtctacgg ccccacctgc accaccatct accagaccgc cggtggcagc aacgactggg

96241 tcgccgacgt gctcggctcc gagctgccgt gggcctttga gatgaggccc cagggcgccg

96301 cggagggcgg cttcgtcatc tcgcctgaca acatcgtccc cagcggcgag gagatctggg

96361 ctggcatgaa ggacctgttt aagaactggt agatggagca aagttttcag agcgttgggt

96421 ggttggaatc tggggattcc gtgtctggtc cagttgtata ttcttttcta tttcgagccc

96481 catcaacact gacaggccaa tctagactag agtagactag tagaggtagt agtagtagta

96541 gtagtattag ttaacgccgg gctcggcccg gcttgttagc cggccgttag caacctcccg

96601 aggggtatct cggcgcgcgt tctatcttct ttatttacac agggggaaaa caaggagggc

96661 agaggcggat cgtgcctgac cgggttcggg cccggtcctg cttagggggt tagttcactg

96721 acgcgacccc gtgcctaagg tgcacggagg gttcgtctga cggcttgtgc cgtgaagtgt

96781 gggtgaaaag gcagtaagtc tattcctcgt cagagttcga gtcgtttacg atcggtgtcc

96841 ctaggcgtaa agtgcgttcg tggcgcgcgg ggcgctggcg ggccgctctg gggcgggcgc

96901 tgaagtagtt ggtggctatc gaaaacgcct caaagctttt cggttgcccg aagcttgtct

96961 ggaagaattt ccagcgttgg gcgcgatttg gcggcccggc cggccggtcg ttattaggcc

97021 acggccagtt tctccacacc gcccgcgaat agcggcagtg caccgggtgc tcaggggagg

97081 tccgcttcca gcaccaggca cacgaggtgt ttgcatcctg gtggttgaaa cggtcatggt

97141 aggctttgaa atcgccgtgg ccggtcctca tggccaaata atggcccagt aggggtctt(MGL, 5307-5977, str -)c

97201 cttgcatttc ctacagtaaa ctatagtcac agggttggac cagaccctct aacggacagg

97261 ccagctagag ccctcctagt cgctggatac ccagcctgcc acgtaaatac aaggtttgca

97321 ccttaagcct accgcatcca caaaaaggcc tcttggtaga ccgacgcgcg catccgtcct

97381 ctccggctaa aatagcgtct cttccgccta acatgccgac gacatcatct gccccggtgt

97441 acgtccctag tgcgagtaga aagcttcgaa aagccggcga caccccgtac atgtccatcc

97501 acagcttagt cccccgttgg ttcctgggct agcccagcgc cacaatgtgg acatgaatgc

97561 aatcgagttc ttcccgtcct ccagacccgg cgcagattcc cagtgcgagt gagaagcatg

97621 gggcgcctca gtcacactat tccaccacgg cccaccgtcc acggggtgta ccaagggtgc

97681 gaaccaaggc cgtgcccacg tttcttgctt cccgttaccc aaagccgtct ccagcaatgg

97741 gccgaccacg tcttgctgtc gggacccttt ggagactgcc gcgccggcac agtgcttccg

97801 gtccacgtac aactaaccaa cccccgtaac ggcactacct cctcgctgca ggcaatccca

97861 gaaggtgtga gggggggtga gagggtgccc tgtcgattcg aagaaccagt cgggtggttc

97921 tcagcctaat ttccctttct tttaaaccgt attgtctagc ctttccgggg aaggacgcct

97981 gcctttccgg ctggcaaaag tttttttgtg ccacgctggc tcgcatcgcg agattgggcc

98041 ttgtcttgtg cgcctctatg cgggtggcgc gccgaaccgc cgatcggact cttccttcac

98101 cgcgatcagt ggaaaactcg tcaaagggga gatctgtcga ttatacatgg gtgttgcgag

98161 gaaaggcgtt ccgtgtagtg acggtgctta gggcgacagg ttgaagagtt tttcgtgtgt

98221 gcgcgcctgt tagggctaca ggcggaagtc ccgcgcaaac gttaacagca tggtccgtgc

98281 gcccatactg aaaattttgt gtacgggaga gagttg(WEIRD, str -)tcat cttttttttt atgtatactg

98341 ccacagtata tcaaagtcga caacccgtct cttgtgctat ttctccgatg ggagttggat

98401 aggtaaggtg tctaggtaac gataccgcat ccagtagaca aagacggccg tcgacagtca

98461 caagacacga taaccaagta ccctaagccg ggtggtttgg atccatggac tatggtatat

| 12 | AACU02000173 |
| --- | --- |

257401 gccactatgt tatatattca ttgcctaccg tgtaggtcac acgaatataa tgctttttca

257461 aaagttatta gagtcataat agggactttg gaaattctga tgcttggaaa aagtagaaat

257521 gttttcttgt tagtctaaga gtgtgagctg agatactttg tctggagtga tgaggtgcaa

257581 gtctctgtgt taccttagcg tgaaaggttt gggttttcac agcatgaggc aggtggt**tta**

257641 **tcaggtattc agt**agtagta gtagtagtag tattagttaa cgccgggctc ggcccggctt

257701 gttagccggc cgttagcaac ctcccgaggg gtatctcggc gcgcgttcta tcttctttat

257761 ttacacaggg ggaaaacaag gagggcagag gcggatcgtg cctgactggg ttcgggcccg

257821 gtcctgctta gggggttagt tcactgacgc gaccccgtgc ctaaggtgca cggagggttc

257881 gtctgacggc ttgtgccgtg aagtgtgggt gaaaaggcag taagtctatt cctcgtcaga

257941 gttcgagtcg tttacgatcg gtgtccctag gcgtaaagtg cgttcgtggc gcgcggggcg

258001 ctggcgggcc gctctggggc gggcgctgaa gtagttggtg gctatcgaaa acgcctcaaa

258061 gcttttcggt tgcccgaagc ttgtctggaa gaatttccag cgttgggcgc gatttggcgg

258121 cccggccggc cggtcgttat taggccacgg ccagtttctc cacaccgccc gcgaatagcg

258181 gcagtgcacc gggtgctcag gggaggtccg cttccagcac caggcacacg aggtgtttgc

258241 atcctggtgg ttgaaacggt catggtaggc cttgaaatcg ccgtggccgg tcctcatggc

258301 caaataatgg cccagtaggg gtcttggcaa acgcagttcc tcg(MGL, 5289-5971, str-)ccttgca tttcctacag

258361 taaactatag tcacagggtt ggaccagacc ctctaacgga caggccagct agagccctcc

258421 tagtcgctgg atacccagcc tgccacgtaa atacaaggtt tgcaccttaa gcctaccgca

258481 tccacaaaaa ggcctcttgg tagaccgacg cgcgcatccg tcctctccgg ctaaaatagc

258541 gtctcttccg cctaacatgc cgacgacatc atctgccccg gtgtacgtcc ctagtgcgag

258601 tagaaagctt cgaaaagccg gcgacacccc gtacatgtcc atccacagct tagtcccccg

258661 ttggttcctg ggctagccca gcgccacaat gtggacatga atgcaatcga gttcttcccg

258721 tcctccagac ccggcgcaga ttcccagtgc gagtgagaag catggggcgc ctcagtcaca

258781 ctattccacc acggcccacc gtccacgggg tgtaccaagg gtgcgaacca aggccgtgcc

258841 cacgtttctt gcttcccgtt acccaaagcc gtctccagca atgggccgac cacgtcttgc

258901 tgtcgggacc ctttggagac tgccgcgccg gcacagtgct tccggtccac gtacaactaa

258961 ccaacccccg taacggcact acctcctcgc tgcaggcaat cccagaaggt gtgagggggt

259021 gagagggtgc cctgtcgatt cgaagaaccg tcgggtggtt ctcagcctaa tttccctttc

259081 ttttaaaccg tattgtctag ccttccgggg aaggacgcct gcctttccgg ctggcaaagt

259141 tttttgtgcc acgctggctc gcatcgcgag attgggcctt gtcttgtgcg cctctatgcg

259201 ggtggcgcgc cgagccgccg atcggactct tccttcaccg cgatcagtgg aaaactcgtc

259261 aaaggggaga tctgtcgatt atacatgggt gttgcgagga aaggcgttcc gtgtagtgac

259321 ggtgcttagg gcgacaggtt gaagagtttt tcgtgtgtgc gcgcctgtta gggctacagg

259381 cggaagtccc gcgcaaacgt taacagcatg gtccgtgcgc ccatactgaa aattttgtgt

259441 acgggagaga gttg(WEIRD,str-)**ttatca** **ggtattcagt** tagcccgccg gggatattcc aacttcaccc

259501 aaattactgc ttggtgattc ttcgtaccat ctagacgtta tacagaattg tttgatttac

259561 gttgagcgaa tctctataga tcatcactgt ctttacctgt atcaaggctc actacggtca

259621 agtagttttc aacagcctgc cacggtgcgg atgttagcat tttcaaacgt aggtaaacaa

259681 tcactttgag gagaatctac gacagctgcc tggcttgggt ttattttgtc cttgactccc

| 13 | AACU02000326 |
| --- | --- |

82021 ccaactggta catgttcggg ctggcgtaca aagacagcag ctggagacat cgtgaaaacg

82081 gcgacggcac catgacacaa gttgaagccg agccgatgga tgtacactcc aaatcaagat

82141 ggattttcaa aggagtgatt gtagaccctg agataaacgt gccagggtcg ccagttctca

82201 tcggaatagg caagtttttt gtttcatacc aagtccctac aggaccaaca caccccgatt

82261 ctaacagttg atacgcaacg atagccaatt ctttgagcgg aggcagattt tcccaccgga

82321 cttggaattg ccgcatattt gttgagaggt ttttggaggg gcttgaacct tttctagaca

82381 agagctatgc cgactagaag taggggcttt tattgtcctt tagactccga aggaaaggag

82441 tcaggaatga ttgcatcaag catgttcatc ctcgaggcga cggagagtag cctgagcggt

82501 acaacctgga catggctgc**g** **agtaaacatt** **taa**gtagtag tagtagtagt attagttaac

82561 gccgggctcg gcccggcttg ttagccggcc gttagcaacc tcccgagggg tatctcggcg

82621 cgcgttctat cttctttatt tacacagggg gaaaacaagg agggcagagg cggatcgtgc

82681 ctgaccgggt tcgggcccgg tcctgcttag ggggttagtt cactgacgcg accccgtgcc

82741 taaggtgcac ggagggttcg tctgacggct tgtgccgtga agtgtgggtg aaaaggcagt

82801 aagtctattc ctcgtcagag ttcgagtcgt ttacgatcgg tgtccctagg cgtaaagtgc

82861 gttcgtggcg cgcggggcgc tggcgggccg ctctggggcg ggcgctgaag tagttggtgg

82921 ctatcgaaaa cgcctcaaag cttttcggtt gcccgaagct tgtctggaag aatttccagc

82981 gttgggcgcg atttggcggc ccggccggcc ggtcgttatt aggccacggc cagtttctcc

83041 acaccgcccg cgaatagcgg cagtgcaccg ggtgctcagg ggaggtccgc ttccagcacc

83101 aggcacacga ggtgtttgca tcctggtggt tgaaacggtc atggtaggcc ttgaaatcgc

83161 cgtggccggt cctcatcgcc aaataatggc ccagtagggg tcttggcaaa cgcagttcct

83221 cg(MGL, 5289-5977, str-)accttgca tttcctacag taaactatag tcacagggtt ggaccagacc ctctaacgga

83281 caggccagct agagccctcc tagtcgctgg atacccagcc tgccacgtaa atacaaggtt

83341 tgcaccttaa gcctaccgca tccacaaaaa ggcctcttgg tagaccgacg cgcgcatccg

83401 tcctctccgg ctaaaatagc gtctcttccg cctaacatgc cgacgacatc atctgccccg

83461 gtgtacgtcc ctagtgcgag tagaaagctt cgaaaagccg gcgacacccc gtacatgtcc

83521 atccacagct tagtcccccg ttggttcctg ggctagccca gcgccacaat gtggacatga

83581 atgcaatcga gttcttcccg tcctccagac ccggcgcaga ttcccagtgc gagtgagaag

83641 catggggcgc ctcagtcaca ctattccacc acggcccacc gtccacgggg tgtaccaagg

83701 gtgcgaacca aggccgtgcc cacgtttctt gcttcccgtt acccaaagcc gtctccagca

83761 atgggccgac cacgtcttgc tgtcgggacc ctttggagac tgccgcgccg gcacagtgct

83821 tccggtccac gtacaactaa ccaacccccg taacggcact acctcctcgc tgcaggcaat

83881 cccagaaggt gtgagggggt gagagggtgc cctgtcgatt cgaagaaccg tcgggtggtt

83941 ctcagcctaa tttccctttc ttttaaaccg tattgtctag ccttccgggg aaggacgcct

84001 gcctttccgg ctggcaaagt tttttttttt ttgtgccacg ctggctcgca tcgcgagatt

84061 gggccttgtc ttgtgcgcct ctatgcgggt ggcgcgccga accgccgatc ggactcttcc

84121 ttcaccgcga tcagtggaaa actcgtcaaa ggggagatct gtcgattata catgggtgtt

84181 gcgaggaaag gcgttccgtg tagtgacggt gcttagggcg acaggttgaa gagtttttcg

84241 tgtgtgcgcg cctgttaggg ctacaggcgg aagtcccgcg caaacgttaa cagcatggtc

84301 cgtgcgccca tactgaaaat tttgtgtacg ggagagagtt g(WEIRD, str-)**gagtaaaca** **tttaa**gatat

84361 tgttgcaaag atacacggtg ggcttttctt ccctgtgtac ctttggtacc tgtccatatt

84421 actctgctgt agattgttgt ttttaacccg cagctattgt ctacgccttg tgccgcatga

| 14 | AACU02000681 |
| --- | --- |

117721 acgacaaatc agatggtcag aattcctgtc agaattccat tttaggatca tttaccgaaa

117781 aggaacagaa aacggcaggg ccgacgccct cagccgaaga ccagatcacg agaacatagt

117841 gccagaggaa acacgggtta tcctcaccac agacggaaac ggaaaccttt taccagcaca

117901 ccggagcctt atgacaacaa atacggtaac cacaccagaa gaaatacgga agatccacgg

117961 aaacaaagcc cacggacacc aaggaatttc caagacatgg aaacggctaa aacagcatca

118021 caattttaaa ggaacacgac aagaagtacg agaagccatc aaggactgcg aactttgtgc

118081 caagagcaag tccgcaagac ataggcctta cggacaactc tctcaccgta cacaaaattt

118141 tcagtatggg cgcacggacc atgctgttaa cgtttgcgcg ggacttccgc ctgtagccct

118201 aacaggcgcg cacacacgaa aaacttttca acctgtcgcc ctaagcaccg tcactacacg

118261 gaacgccttt cctcgcaaca cccatgtata atcgacagat ctcccctttg acgagttttc

118321 cactgatcgc ggtgaagtaa gagtccgatc ggcggctcgg cgcgccaccc gcatagaggc

118381 gcacaagaca aggcccaatc tcgcgatgcg agccagcgtg gcacaaaaaa ctttgccagc

118441 cggaaaaggc aggcgtcctt tccccggaag gctagacaat acggtttaaa agaaagggaa

118501 attaggctga gaaccacccg acggttcttc gaatcgacag ggcaccctct cacccccctc

118561 acaccttctg ggattgcctg cagcgaggag gtagtgccgt tacgggggtt ggttagttgt

118621 acgtggaccg gaagcactgt gccggcgcgg cagtctccaa agggtcccga cagcaagacg

118681 tggtcggccc attgctggag acggctttgg gtaacgggaa gcaagaaacg tgggcacggc

118741 cttggttcgc acccttggta caccccgtgg acggtgggcc gtggttggaa tagtgtgact

118801 gaggcgcccc atgcttctca ctcgcactgg gaatctgcgc cgggtctgga ggacgggaag

118861 aactcgattg cattcatgtc cacattgtgg cgctgggcta gcccaggaac caacggggga

118921 ctaagctgtg gatggacatg tacggggtgt cgccggcttt tcgaagcttt ctactcgcac

118981 tagggacgta caccggggca gatgatgtcg tcggcatgtt aggcggaaga gacgctattt

119041 tagccggaga ggacggatgc gcgcgtcggt ctaccaagag gcctttttgt ggatgcggta

119101 ggcttaaggt gcaaaccttg tatttacgtg gcaggctggg tatccagcga ctaggagggc

119161 tctagctggc ctgtccgtta gagggtctgg tccaaccctg tgactatagt ttactgtagg

119221 aaatgcaagg(WEIRD, str+) tttctggagt tccataacct tgtcgatcta ctccgaaaac aaggtaccga

119281 ggttcgggtc cgttggtgcc ctgggcacca gggaatcccg ggaaacgacc gggctgacga

119341 gctggccaag gccggctccg ccggaccgcc ggacccagac ccgagggctc agcaaaccac

119401 gtatagcggt gccggcacgg tcctcagagc cattctttcg aatatagaga aggactggtg

119461 gcgtaaagaa ctctgtgaac ggtcccccgc atatagggaa tggaaattcc aatacacacc

119521 gagaaaggag cccgaggaac tgcgtttgcc aagaccccta ctgggccatt acttggccat

119581 gaggaccggc cacggcgatt tcaaagccta ccatgaccgt ttcaaccacc aggatgcaaa

119641 cacctcgtgt gcctggtgct ggaagcggac ctcccctgaa cacccggtgc actgccgctt

119701 ttcgcgggcg gtgtggagaa actggccgtg gcctgacaac gaccggccgg tcgggccgcc

119761 agaccgcgcc caacgccgca aattcttcca gacaagcctc gggcaaccga aaagctttga

119821 ggcgttttcg atagccacca actacttcag cgcccgcccc agagcggccc gccagcgccc

119881 cgcgcgccac gaacgcactt tacgcctagg gacaccgatc gtaaacgact cgaactctga

119941 cgaggaatag acttactgcc ttttcaccca cacttcacgg cacaagccgt cagacgaacc

120001 ctccgtgcac cttaggcacg gggtcgcgtc agtgaactaa ccccctaagc aggaccgggc

120061 ccgaacccgg tcaggcacga tccgcctctg ccctccttgt tttccccctg tgtaaataaa

120121 gaagatagaa cgcgcgccga gatacccctc gggaggttgc taacggccgg ctaacaagcc

120181 gggccgagcc cggcgttaaa taatactact actactacta c(MGL, str+, 4987-5977)tactatagg ccttacggac

120241 tcctacaacc cttaccagcc cccagcaagg catggcagac cattacaatg gacttcatcg

120301 tcaagttacc cccttcggaa gaaccactca ctaagaccaa gtacgacagc atactggtta

120361 tagtggacaa gctcaccaaa tacgcctact tcctaccata caaagaaagc agcaacgccg

| 15 | AACU02000436 |
| --- | --- |

32161 tacatgggga aagtcaatgt ttcccctcac tcttttccaa acgtggtaat taatccaaaa

32221 gcaaaacatg tgaaccccgc ttgtaggtcc ctcgggtccc tcgatcctgt caacgcaaga

32281 agccagagcg tcctgcatcg cgtaccaaca caaggcggct taaaggtttc gtcggacaca

32341 tgctagacct tcaaccgccg tagcgcaacg tgcctgtgat aaagcgattt gaaagccttg

32401 cctggctgtt gaacggaatt gcacagggca aaatgctcaa atgtctatca tgcacacaac

32461 gcttcacagt agatgagtga aagtaatcca aggaagtcga c**gctgctcag** **gcaa**gtagta

32521 gtagtagtag tagtagtagt attatttaac gccgggctcg gcccggcttg ttagccggcc

32581 gttagcaacc tcccgagggg tatctcggcg cgcgttctat tttctcttat ttacacaggg

32641 ggaaaacaag gagggcagag gcggatcgtg cctgaccggg ttcgggcccg gtcctgctta

32701 gggggatagt tcactgacgc gaccccgtgc ctaaggtgca cggagggttc gtctgacggc

32761 ttgtgccgtg aagtgtgggt gaaaaggcag taagtctatt cctcgtctga gttcgagtcg

32821 tttacgatcg gtgtccctag gcgtaaagtg cgttcgtggc gcgcggggcg ctggcgggcc

32881 gctctggggc gggcgctgaa atagttggtg gctatcgaaa acgcctgaaa gcttgtcggt

32941 tgcccgaggc ttgtctggaa gaatttgcgg cgttgggcgc ggtctggcgg cccgaccggc

33001 cggtcgttgt caggccacgg ccagtttctc cacaccgccc gcgaaaagcg gcagtgcacc

33061 gggtgttcag gggaggtccg cttccagcac caggcacacg aggtgtttgc atcctggtgg

33121 ttgaaacggt catggtaggc tttgaaatcg ccgtggccgg tcctcatggc caaataatgg

33181 cccagtaggg gttttggcaa acgcagttcc tcgggctcct ttctcggtgt atattggaat

33241 ttccattccc tatatgcggg ggaccgttca cagagttttt tacgccacca gtccttcttt

33301 atattcgaaa gaatggtttt gaggaccgtg ccggcaccgc tatacgtggt ttgctgagcc

33361 ctcgggtctg ggtccggcgg tccggcggag ccggccttgg ccagctcgtc agcccggtcg

33421 tttcccggga ttccctggtg cccagggcac caacggaccc gaacctcggt actttatttt

33481 cggagtagat cgacaaggtt atggaactcc agaaaggccc attgggacga acgcggcgcg

33541 tcgcctctaa(MGL, 4952-5977, str-) ccttgcattt cctacagtaa attatagtca cagggttgga ccagaccctc

33601 taacggacag gccagctaga gccctcctag tcgctggata cccagcctgc cacgtaaata

33661 caaggtttgc accttaaacc taccgcatcc acaaaaaggc ctcttggtag accgacgcgc

33721 gcatccgtcc tctccggcta aaatagcgtc tcttccgcct aacatgccga cgacatcatc

33781 tgccccggtg tacgtcccta gtgcgagtag aaagcttcga aaagccggcg acaccccgta

33841 catgtccatc cacagcttag tcccccgttg gttcctgggc tagcccagcg ccacaatgtg

33901 gacatgaatg caatcgagtt cttcccgtcc tccagacccg gcgcagattc ccagtgcgag

33961 tgagaagcat ggggcgcctc agtcacacta ttccaccacg gcccaccgtc cacggggtgt

34021 accaagggtg cgaaccaagg ccgtgcccac gtttcttgct tcccgttacc caaagccgtc

34081 tccagcaatg ggccgaccac gtcttgctgt cgggaccctt tggagactgc cgcgccggca

34141 cagtgcttcc ggtccacgta caactaacca acccccgtaa cggcactacc tcctcgctgc

34201 aggcaatccc agaaggtgtg agggggtgag agggtgccct gtcgattcga agaaccgtcg

34261 ggtggttctc agcctaattt ccctttcttt taaaccgtat tgtctagcct tccggggaag

34321 gacgcctgcc tttccggctg gcaaagtttt ttgtgccacg ctggctcgca tcgcgagatt

34381 gggccttgtc ttgtgcgcct ctatgcgggt ggcgcgccga accgccgatc ggactcttcc

34441 ttcaccgcga tcagtggaaa actcgtcaaa ggggagatct gtcgattata catgggtgtt

34501 gcgaggaaag gcgttccgtg tagtgacggt gcttagggcg acaggttgaa gagtttttcg

34561 tgtgtgcgcg cctgttaggg ctacaggcgg aagtcccgcg caaacgttaa cagcatggtc

34621 cgtgcgccca tactgaaaat tttgtgtacg ggagagagtt g(WEIRD, str-)**gctgctcag** **gcaa**ggagtg

34681 ttgtgagaca cggcagaata agaattagcc cgcccagcgt aatgcttgcg atataagcgc

34741 gtctggaata tgcctcgtca tttttcgaca tttagtggat gcaaagagcg acagcatagg

34801 gcgcccgtaa ctcccacccc cacgacctgc agcgttatag ctctggacaa catagactgg

34861 ctccgaccct ggattctatt cttctctctt tcagagttta tttgtcccct tgacagaccc

34921 ctgacaacgg aaccagacat ttgacatcca acatacccgg ccgggtcgac actaaagtca

| 16 | AACU02000589 |
| --- | --- |

55441 gggggtgatg agcatggact tttgcttgct ctggcggacc agcgccgggc tgagtatctt

55501 ggcggcgtac agggttggca cccgcataaa gtctccgtac ccctggccga tctggaaggc

55561 cgtgttgaca atgtcgccca ggctgtgcat gttgacaaag tcggcaaacg aaatcatgag

55621 ctcgtcgggt acctggtccg ggaggtagta tcccctgtcc aggtatgaga aattggactc

55681 caccagcttc gcccagcggt ccagaccgtc ctgcaccgcc tggcccggag gcggttcaaa

55741 gccgacctcc tggccgcttt taaagtcgta atatgaactc tctccccgta cacaaaattt

55801 tcagtatggg cgcacggacc atgctgttaa cgtttgcgcg ggacttccgc ctgtagccct

55861 aacaggcgcg cacacacgaa aaactcttca acctgtcgcc ctaagcaccg tcactacacg

55921 gaacgccttt cctcgcaaca cccatgtata atcgacagat ctcccctttg acgagttttc

55981 cactgatcgc ggtgaaggaa gagtccgatc ggcggttcgg cgcgccaccc gcatagaggc

56041 gcacaagaca aggcccaatc tcgcgatgcg agccagcgtg gcacaaaaaa ctttgccagc

56101 cggaaaggca ggcgtccttc cccggaaggc tagacaatac ggtttaaaag aaagggaaat

56161 taggctgaga accacccgac ggttcttcga atcgacaggg caccctctca ccccctcaca

56221 ccttctggga ttgcctgcag cgaggaggta gtgccgttac gggggttggt tagttgtacg

56281 tggaccggaa gcactgtgcc ggcgcggcag tctccaaagg gtcccgacag caagacgtgg

56341 tcggcccatt gctggagacg gctttgggta acgggaagca agaaacgtgg gcacggcctt

56401 ggttcgcacc cttggtacac cccgtggacg gtgggccgtg gtggaatagt gtgactgagg

56461 cgccccatgc ttctcactcg cactgggaat ctgcgccggg tctggaggac gggaagaact

56521 cgattgcatt catgtccaca ttgtggcgct gggctagccc aggaaccaac gggggactaa

56581 gctgtggatg gacatgtacg gggtgtcgcc ggcttttcga agctttctac tcgcactagg

56641 gacgtacacc ggggcagatg atgtcgtcgg catgttaggc ggaagagacg ctattttagc

56701 cggagaggac ggatgcgcgc gtcggtctac caagaggcct ttttgtggat gcggtaggct

56761 taaggtgcaa accttgtatt tacgtggcag gctgggtatc cagcgactag gagggctcta

56821 gctggcctgt ccgttagagg gtctggtcca accctgtgac tatagtttac tgtaggaaat

56881 gcaagg(WEIRD, str+)gctt ttagggcggg cctggaattt gccgcaggct ccgcatctat tacgccggaa

56941 agccacgttt tcgacgccga ggcgattggc gccctaaaag ggctacaggc ggcagccaag

57001 gcccagccag gcgcccggat ttggatttgt gtggacagca cctcggttat ttggggtctt

57061 agaggcgacg cgccgcgttc gtcccaatgg gcctttctgg agttccataa ccttggtcga

57121 tctactccga aaacaaggta ccgaggttcg ggtccgttgg tgccctgggc accagggaat

57181 cccgggaaac gaccgggctg acgagctggc caaggccggc tccgccggac cgccggaccc

57241 agacccgagg gctcagcaaa ccacgtatag cggtgccggc acggtcctca gagccattct

57301 ttcgaatata gagaaggact ggtggcgtaa agaactctgt gaacggtccc ccgcatatag

57361 ggaatggaaa ttccaataca caccgagaaa ggagcccgag gaactgcgtt tgccaagacc

57421 cctactgggc cattatttgg ccatgaggac cggccacggc gatttcaagg cctaccatga

57481 ccgtttcaac caccaggatg caaacacctc gtgtgcctgg tgctggaagc ggacctcccc

57541 tgagcacccg gtgcactgcc gctattcgcg ggcggtgtgg agaaactggc cgtggcctga

57601 caacgaccgg ccggtcgggc cgccagaccg cgcccaacgc cgcaaattct tccagacaag

57661 cctcgggcaa ccgacaagct ttcaggcgtt ttcgatagcc accaactact tcagcgcccg

57721 ccccagagcg gcccgccagc gccccgcgcg ccacgaacgc actttacgcc tagggacacc

57781 gatcgtaaac gactcgaact ctgacgagga atagacttac tgccttttca cccacacttc

57841 acggcacaag ccgtcagacg aaccctccgt gcaccttagg cacggggtcg cgtcagtgaa

57901 ctaaccccct aagcaggacc gggcccgaac ccggtcaggc acgatccgcc tctgccctcc

57961 ttgttttccc cctgtgtaaa taaagaagat agaacgcgcg ccgagatacc cctcgggagg

58021 ttgctaacgg ccggctaaca agccgggccg agcccggcgt taactaatac tactactact

58081 actac(MGL, str+, 4867-5977) tacta aagtcgtaat atttgattgc gtggctgggg tcgttgatgg gcggtatctg

58141 ggagaagctg acgttgtacc gctgcatgta gtcgcgcatc ttcggggtgt cctggaaaaa

58201 gacattgcca tactcgatgg ccacgccgct ggcagggtcg gtataagtct gcgtgtgtcc

58261 acctaggttg ctctgcttct caaccactgc gaagctgtac cctctatcat gcagccaggt

58321 tgccgcgtag ctgccggtca cgccgccgcc gatgatggtc acgtcgcggg tgagcacatt

58381 cgacgttgag tttgggcctc cagcgtcggt atccgagtcc acgctgccgg ccgcgttggc

58441 agcggcaagg aatacggtgg ccagcgtccg ggtggcgtgc atgatggtag ttgttgacaa

| 17 | AACU02000448 |
| --- | --- |

96781 gggcgggcag ctgtacataa tcgggcggtc caaagacatg atcatccgag gcggggaaaa

96841 catcgccccg gccgccatcg agtcgcgtct tgccgagaac cccaacctct cccggcttgc

96901 cattcagatc gtcggagccc ccgaccccat cgccggccag gtccctgttg ccgtcatcga

96961 ggcaagtgcc gagaggctga agctggtcgc aaaggaaatc cacgacactg tcctcgccaa

97021 gatgggaccc atgtttgtac caacccaaat tctcccgctc caggaactcg gcctggccac

97081 ttggccgcga accactacgg gcaagatcaa gaagccgccc gtggcagacg cggtcgagaa

97141 gctcttgagg cagcaagagg attctgagtc ccggaacgac gtgggtcccg gggaagcaag

97201 atcaaattca agcagagcct cgtgatcagg atatggtctc gctccgtcgg cctcccagaa

97261 gccagcctgt cgctgcagca accaattgct cagtttgcaa ctctctcccg tacacaaaat

97321 tttcagtatg ggcgcacgga ccatgctgtt aacgtttgcg cgggacttcc gcctgtagcc

97381 ctaacaggcg cgcacacacg aacaactctt caacctgtcg ccctaagcac cgttactaca

97441 cggaacgcct ttcctcgcaa cacccatgta taatcgacag atctcccctt tgacgagttt

97501 tccactgatc gcggtgaagg aagagtccga tcggcggctc ggcgcgccca cccgcataga

97561 ggcgcacaag acaaggccca atctcgcgat gcgagccagc gtggcacaaa aaactttgcc

97621 agccggaaag gcaggcgtcc ttccccggaa ggctagacaa tacggtttaa aagaaaggga

97681 aattaggctg agaaccaccc gaccgggttc ttcgaatcga caagggcacc ctcttcaccc

97741 cccctcacac cttctgggat tgcctgcagc gaggaggtag tgccgttacg ggggttggtt

97801 agttgtacgt ggaccggaag cactgtgccc ggcgcggcag tctccaaagg gtcccgacag

97861 caagacgtgg tcggcccatt gctggagacg gctttgggta acgggaagca agaaacgtgg

97921 gcacggcctt ggttcgcacc cttggtacac cccgtggacg gtgggccgtg gtggaatagt

97981 gtgactgagg cgccccatgc ttctcactcg cactgggaat ctgcgccggg tctggaggac

98041 gggaagaact cgattgcatt catgtccaca ttgtggcgct gggctagccc aggaaccaac

98101 gggggactaa gctgtggatg gacatgtacg gggtgtcgcc ggcttttcga agctttctac

98161 tcgcactagg gacgtacacc ggggcagatg atgtcgtcgg catgttaggc ggaagagacg

98221 ctattttagc cggagaggac ggatgcgcgc gtcggtttac caagaggcct ttttgtggat

98281 gcggtaggct taaggtgcaa accttgtatt tacgtggcag gctgggtatc cagcgactag

98341 gagggctcta gctggcctgt ccgttagagg gtctggtcca accctgtgat tataatttac

98401 tgtaggaaat gcaagggatc cggagactag agttgcgcgc acctcgcaat gccccggatt

98461 ctcgcactga ccccaccgga ggagtcccga aagaggaagc ggcccgccgc tttatcgaat

98521 ggctggacat ggtatcacct gacgatatcg tggtatatac ggatggttcg gaaaaacacg

98581 aaaacaattg cgtccagata gggtacggat gggccgcttt tagggcgggc ctggaatttg

98641 ccgcaggctc cgcatttatt acgccggaaa gccacgtttt cgacgccgag gcgattggcg

98701 ccctaaaagg gctacaggcg gcaaccaagg cccaaccagg cgcccggatc tggatttgtg

98761 tggacagcac ctcggttatt tggggtctta gaggcgacgc gccgcgttcg tcccaatggg

98821 cctttctgga gttccatgac cttgtcgatt tactccgaaa acaaggtacc gaggttcggg

98881 tccgttagtg ccctgggcac cagggaatcc cgggaaacga ccgggctgac gagctggcca

98941 aggccggctc cgccggaccg ccggacccag acccgagggc tcagcaaacc acgtatagcg

99001 gtgccggcac ggttctcaga gccattcttt cgaatataga gaaggactgg tggcgtaaag

99061 aactctgtaa acggtccccc gcatataggg aatggaaatt ccaatacaca ccgagaaagg

99121 agcccgagga actgcgtttg ccaagacccc tactgggcca ttatttggcc atgaggaccg

99181 gccacggcga tttcaaagcc taccataacc gtttcaacca ccaggatgca aacacctcgt

99241 gtgcctggtg ctggaagcgg acctccccta agcacccggt gcactgccgc tattcgcggg

99301 cggtgtggag aaactggccg tggcttaata acaagcggcc ggtcaggccg ccagaccgcg

99361 cccaacgccg caaattcttc cagacaagcc tcgggcaacc gacaagcttt caggcgtttt

99421 cgatagccac caactacttc agcgcccgcc ccagagcggc ccgccagcgc cccgcgcgcc

99481 acgaacgcac tttacgccta gggacaccga tcgtaaacga ctcgaactca gacgaggaat

99541 agacttactg ccttttcacc cacacttcac ggcacaagcc gtcagacgaa ccctccgtgc

99601 accttaggca cggggtcgcg tcattgaact aaccccctaa gcaggaccgg gcccgaaccc

99661 ggtcaggcac gatccgcctc tgccctcctt gttttccccc tgtgtaaata aagaagatag

99721 aacgcgcgcc gagatacccc tcgggaggtt gctaacggcc ggctaacaag ccgggccgag

99781 cccggcgtta actgatacta ctactactac tac(MGL, str+, 4580-5977)tacagca tcctgactac cagaccatat

99841 cccagtgagc cacgcggtgc tggacgcaag tagcatatgg atgttccagg aggatttgga

99901 caccattctg tcaggcaagc aacttcaacc ccacgtggat tacaagctct gggccgacgc

99961 gtactattcc tttcgcgcgt ctcctcatgc tcgcaagtca ctcaactggc acgcaagata

100021 cctaaacggt ctcgagagcc atctagacaa ggccctttgg ccacccctcc cgccacgcga

| 18 | AACU02000448 |
| --- | --- |

1 gtagtagtag tagtagtagt attatttaac accgggctcg gcccggcttg ttaaccggcc

61 gttagcaacc tcccgagggg gtatctcggc gcgcgttcta tcttctttat ttacacaggg

121 ggaaaacaag gagggcagag gcggatcgtg cctgaccggg ttcgggcccg gtcctgttta

181 gggggatagt tcactaacgc gaccccgtgc ctaagatgca cggagggttc gtctgacggc

241 ttgtgccgtg aattgtgggt gaaaaggcag caagtctatt cctcgtctga gttcgagtcg

301 tttacgatcg gtgtccctag gcgtaaagtg cgttcgtgac gcgcggggcg ctggcgggcc

361 gctctggggc gggcgctgaa gtagttggtg gctatcgaaa acgcctgaaa gcttgtcggt

421 tgcccgaggc ttgtctggaa gaatttgcgg cgttgggcgc ggtctgccgg cccgaccggc

481 cgcttgttat taagccacgg ccagtttctc cacaccgccc gcgaatagcg ccagtgcacc

541 cgcccttcag gggaggtccg cttacagcac caggcacacg aggtgtttgc atcctggtgg

601 ttgaaacggt catggtaggc tttgaaatcg ccgtggccgg tcctcatggc caaataatgg

661 cccagtaggg gtcttggcaa acgcagttcc tcgggctcct ttctcggtgt gtattggaat

721 ttccattccc tatatgcggg ggaccgttta cagagttctt tacgcccacc agtccttctc

781 tatattcgaa agaatggctc tgagaaccgt gccggcaccg ctatacgtgg tttgctgagc

841 cctcgggtct gggtccggcg gtccggcgga gccggccttg gccagctcgt cagcccggtc

901 gtttcccggg attccctggt gcccagggca ctaacggacc cgaacctcgg taccttgttt

961 tcggagtaaa tcgacaaggt catggaactc cagaaaggcc cattgggacg aacgcggcgc

1021 gtcgcctcta agaccccaaa taaccgaggt gctgtccaca caaatccaga tccgggcgcc

1081 tggttgggcc ttggttgccg cctgtagccc ttttagggcg ccaatcgcct cggcgtcgaa

1141 aacgtggctt tccggcgtaa taaatgcgga gcctgcggca aattccaggc ccgccctaaa

1201 agcggcccat ccgtacccta tctggacgca attgttttcg tgtttttccg aaccatccgt

1261 atataccacg atatcgtcag gtgataccat gtccagccat tcgataaagc ggcgggccgc

1321 ttcctctttc gggactcctc cggtggggtc agtgcgagaa tccggggcat tgcgaggtga

1381 gcgcaactct agtctccaga tcc(MGL, str-, 4580-5977)cttgcat ttcctacaaa aaatgataat cacagggttg

1441 gaccagaccc tctaacggac aggccagcta gagccctcct agtcgctgga tacccagcct

1501 gccacgtaaa tacaaggttt gcaccttaag cctaccgcat ccacaaaaag gcctcttggt

1561 aaaccgacgc gcgcatccgt cctctccggc taaaatagcg tctcttccgc ctaacatgcc

1621 gacgacatca tctgccccgg tgtacgtccc tagtgcgagt agaaagcttc gaaaagccgg

1681 cgacaccccg tacatgtcca tccacagctt agtcccccgt tggttcctgg gctagcccag

1741 cgccacaatg tggacatgaa tgcaatcgag ttcttcccgt cctccagacc cggcgcagat

1801 tcccagtgcg agtgagaagc atggggcgcc tcagtcacac tattccacca cggcccaccg

1861 tccacggggt gtaccaaggg tgcgaaccaa ggccgtgccc acgtttcttg cttcccgtta

1921 cccaaagccg tctccagcaa tgggccgacc acgtcttgct gtcgggaccc tttggagact

1981 gccgcgccgg cacagtgctt ccggtccacg tacaactaac caacccccgt aacggcacta

2041 cctcctcgct gcaggcaatc ccagaaggtg tgagggggtg agagggtgcc ctgtcgattc

2101 gaagaaccgt cgggtggttc tcagcctaat ttccctttct tttaaaccgt attgtctagc

2161 cttccgggga aggacgcctg cctttccggc tggcaaagtt ttttgtgcca cgctggctcg

2221 catcgcgaga ttgggccttg tcttgtgcgc ctctatgcgg gtggcgcgcc gagccgccga

2281 tcggactctt ccttcaccgc gatcagtgga aaactcgtca aaggggagat ctgtcgatta

2341 tacatgggtg ttgcgaggaa aggcgttccg tgtagtgacg gtgcttaggg cgacaggttg

2401 aagagttttt cgtgtgtgcg cgcctgttag ggctacaggc ggaagtcccg cgcaaacgtt

2461 aacagcatgg tccgtgcgcc catactgaaa attttgtgta cgggagagag ttg(WEIRD, str-)atgggag

2521 caagtgacgt gggtcgtggg tacgccggcg ggaaaaaggg gcagctcggc tccctggtgg

2581 accgaaaact gccagcggct ctggtccgaa ttccagcggg ttaaacggag cgctgtaaat

2641 agggcagacg cctctgccga ggaaaaagcc tatacgaaag gggtgcgggc cgcgaagcgg

2701 gagtactgga ggcaccggat cgaccaactg cgcgacgata aggatttgtg gaacatgggt

2761 ggggctgggc tgggaaccgg accacgcctc cggtccccgc cgctggttat taacggcgag

| 19 | AACU02000346 |
| --- | --- |

7801 gccagggttt accagtggtc gcacggcacg tacattgagg atcaggacta gtggtggctg

7861 tctgggatct tcagggacgt ctacctgatt ccgtttgcac agtcgtccat tgtggatttc

7921 gaggtgcatc cggaactcga cgcagccctc gaaaccggca cgcttcgcac tgtggtcaaa

7981 gtacagggcg atgacgggcc tctgaagtag tagtagtagt agtagtagta gtagtagtag

8041 tagtagtagt agtagtagta gtagtagtag tagtagtagt agtagtagta gtagtagtag

8101 tagtagtagt agtattattt aacgccgggc tcggcccggc ttgttagccg gccgttagca

8161 acctcccgag gggtatctcg gcgcgcgttc tatcttcttt atttacacag ggggaaaaca

8221 aggagggcag aggcggatcg tgcctgaccg ggttcgggcc cggtcctgct tagggggtta

8281 gttcactgac gcgaccccgt gcctaaggtg cacggagggt tcgtctgacg gcttgtgccg

8341 tgaagtgtgg gtgaaaaggc agtaagtcta ttcctcgtca gagttcgagt cgtttacgat

8401 cggtgtccct aggcgtaaag tgcgttcgtg gcgcgcgggg cgctggcggg ccgctctggg

8461 gcgggcgctg aagtagttgg tggctatcga aaacgcctca aagcttttcg gttgcccgaa

8521 gcttgtctgg aagaatttcc agcgttgggc gcgatttggc ggcccggccg gccggtcgtt

8581 attaggccac ggccagtttc tccacaccgc ccgcgaatag cggcagtgca ccgggtgctc

8641 aggggaggtc cgcttccagc accaggcaca cgaggtgttt gcatcctggt ggttgaaacg

8701 gtcatggtag gccttgaaat cgccgtggcc ggtcctcatg gccaaataat ggcccagtag

8761 gggtcttggc aaacgcagtt cctcgggctc ctttctcggt gtgtattgga atttccattc

8821 cctatatgcg ggggaccgtt cacagagttc tttacgccac cagtccttct ctatattcga

8881 aagaatggct ctgaggaccg tgccggcacc gctatacgtg gtttgctgag ccctcgggtc

8941 tgggtccggc ggtccggcgg agccggcctt ggccagctcg tcagcccggt cgtttcccgg

9001 gattccctgg tgcccagggc accaacggac ccgaacctcg gtaccttgtt ttcggagtag

9061 atcgacaagg ttatggaact ccagaaaggc ccattgggac gaacgcggcg cgtcgcctct

9121 aagaccccaa ataaccgagg tgctgtccac acaaatccag atccgggcgc ctggctgggc

9181 cttggctgcc gcctgtagcc cttttagggc gccaatcgcc tcggcgtcga aaacgtggct

9241 ttccggcgta atagatgcgg agcctgcggc aaattccagg cccgccctaa aagcggccca

9301 tccgtaccct atttggacgc agttgttttc gtgtttttcc gaaccatccg tatataccac

9361 gatatcgtca ggtgatacca tgtccagcca ttcgataaag cggcgggccg cttcctcttt

9421 cgggactcct ccggtggggt cagtgcgaga atccggggca ttgcgaggtg cgcgcaactc

9481 tagtctccgg atccgcggga gaagttgtgc agccgtttgc agggtcgtgg ccgaatggcc

9541 cgagttgcgg gcacgaggtg tttcacgcag gcggctgaca agggggtgcc ccttgtccgc

9601 gagccttagt cg(MGL, str-, 4462-5977)ccttgcat ttcctacagt aaattatagt cacagggttg gaccagaccc

9661 tctaacggac aggccagcta gagccctcct agtcgctgga tacccagcct gccacgtaaa

9721 tacaaggttt gcaccttaag cctaccgcat ccacaaaaag gcctcttggt agaccgacgc

9781 gcgcatccgt cctctccggc taaaatagcg tctcttccgc ctaacatgcc gacgacatca

9841 tctgccccgg tgtacgtccc tagtgcgagt agaaagcttc gaaaagccgg cgacaccccg

9901 tacatgtcca tccacagctt agtcccccgt tggttcctgg gctagcccag cgccacaatg

9961 tggacatgaa tgcaatcgag ttcttcccgt cctccagacc cggcgcagat tcccagtgcg

10021 agtgagaagc atggggcgcc tcagtcacac tattccacca cggcccaccg tccacggggt

10081 gtaccaaggg tgcgaaccaa ggccgtgccc acgtttcttg cttcccgtta cccaaagccg

10141 tctccagcaa tgggccgacc acgtcttgct gtcgggaccc tttggagact gccgcgccgg

10201 cacagtgctt ccggtccacg tacaactaac caacccccgt aacggcacta cctcctcgct

10261 gcaggcaatc ccagaaggtg tgagggggtg agagggtgcc ctgtcgattc gaagaaccgt

10321 cgggtggttc tcagcctaat ttccctttct tttaaaccgt attgtctagc cttccgggga

10381 aggacgcctg cctttccggc tggcaaagtt tttttgtgcc acgctggctc gcatcgcgag

10441 attgggcctt gtcttgtgcg cctctatgcg ggtggcgcgc cgagccgccg atcggactct

10501 tccttcaccg cgatcaatgg aaaactcgtc aaaggggaga tctgtcgatt atacatgggt

10561 gttgcgagga aaggcgttcc gtgtagtgac ggtgcttagg gcgacaggtt gaagagtttt

10621 tcgtgtgtgc gcgcctgtta gggctacagg cggaagtccc gcgcaaacgt taacagcatg

10681 gtccgtgcgc ccatactgaa aattttgtgt acgggagaga gttg(WEIRD, str-)gtatat catggacagg

10741 aagtacgcat caccttgaac ccagcgtttt cacacttgtg gtctaggttc caacgtgcac

10801 ataatatcgt cttgcagtac aatactggta ttaaccggat caggcccttt agccaacatt

10861 ctaacctctg cattggccca gaggaaaacc caagccagcc ttgtattgac atgtgattca

| 20 | AACU02000308 |
| --- | --- |

24001 cttttagcag catcgatgag atcctgatca gagatcttgt ttgactggca cgtggtggtg

24061 taaagtttgc gaagctcagc ggccgttttg cgccagcgct tgtgattctc gagggcttcg

24121 gcgtccgcgg atacacgatt cgtctgctct gtggtaaggc tagactccag ctgctgcacg

24181 agggcttggg cttcagaaaa ctgcttctgt aagcttgcca gttcggcttg gtgacaagct

24241 tgaaagtttt gggtcttctg atgccattct tggtttattt cagcaatacg ggattgcagg

24301 taagcttccc agtctttttg ctgcttttca gtccaatacc gtatcaatct cgaattgttg

24361 tttgttgctt cttccaattc tcggatattt ctgttgcatt ctgcgagttg ttcctggcag

24421 ttggctaccc gccacaggtg cattgtgtgg tccaagtcgc tacataagct ggacacaggc

24481 ttgtgactgt tacaatcgtc gccatctttc tcaacaggta ccctgctagt cgtgcatgcg

24541 cacgaaggcg acggtgattt gttggcgcgg atgcagtcca tcgcctcttc catgctggtg

24601 tcctcgtcta tgtacaactc tctcccgtac acaaaatttt cagtatgggc gcacggacca

24661 tgctgttaac gtttgcgcgg gacttccgcc tgtagcccta acaggcgcgc acacacgaaa

24721 aactcttcaa cctgtcgccc taagcaccgt cactacacgg aacgcctttc ctcgcaacac

24781 ccatgtataa tcgacagatc tcccctttga cgagttttcc actgatcgcg gtgaaggaag

24841 agtccgatcg gcggctcggc gcgccacccg catagaggcg cacaagacaa ggcccaatct

24901 cgcgatgcga gccagcgtgg cacaaaaaaa aaaaaaaaaa aaactttgcc agccggaaag

24961 gcaggcgtcc ttccccggaa ggctagacaa tacggtttaa aagaaaggga aattaggctg

25021 agaaccaccc gacggttctt cgaatcgaca gggcaccctc tcaccccctc acaccttctg

25081 ggattgcctg cagcgaggag gtagtgccgt tacgggggtt ggttagttgt acgtggaccg

25141 gaagcactgt gccggcgcgg cagtctccaa agggtcccga cagcaagacg tggtcggccc

25201 attgctggag acggctttgg gtaacgggaa gcaagaaacg tgggcacggc cttggttcgc

25261 acccttggta caccccgtgg acggtgggcc gtggtggaat agtgtgactg aggcgcccca

25321 tgcttctcac tcgcactggg aatctgcgcc gggtctggag gacgggaaga actcgattgc

25381 attcatgtcc acattgtggc gctgggctag cccaggaacc aacgggggac taagctgtgg

25441 atggacatgt acggggtgtc gccggctttt cgaagctttc tactcgcact agggacgtac

25501 accggggcag atgatgtcgt cggcatgtta ggcggaagag acgctatttt agccggagag

25561 gacggatgcg cgcgtcggtc taccaagagg cctttttgtg gatgcggtag gcttaaggtg

25621 caaaccttgt atttacgtgg caggctgggt atccagcgac taggagggct ctagctggcc

25681 tgtccgttag agggtctggt ccaaccctgt gactatagtt tactgtagga aatgcaagga

25741 aaaggactcc tcaaagcatt gaacaaaccg ctggttttag cggcacgggc aatcctcccg

25801 gcgtataaaa ccaccccctc gtccactgtt ctcagggacg caggactacc ctcggcccgc

25861 gtcgcgctgg cctacacccg cctgaaatac ggcgcccgac taaggctcgc ggacaagggg

25921 cacccccttg tcagccgcct gcgtgaaaca cctcgtgccc gcaactcggg ccattcggcc

25981 acgaccctgc aaacggctgc acaacttctc ccgcggatcc ggagactaga gttgcgcgca

26041 cctcgcaatg ccccggattc tcgcactgac cccaccggag gagtcccgaa agaggaagcg

26101 gcccgccgct ttatcgaatg gctggacatg gtatcacctg acgatatcgt ggtatatacg

26161 gatggttcgg aaaaacacga aaacaactgc gtccaaatag ggtacggatg ggccgctttt

26221 agggcgggcc tggaatttgc cgcaggctcc gcatctatta cgccggaaag ccacgttttc

26281 gacgccgagg cgattggcgc cctaaaaggg cttacaggcg gcagccaagg ctccagccag

26341 gcgcccggga tttggatttg tgtggacagc acctcggtta tttggggtct tagaggcgac

26401 gcgccgcgtt cgtcccaatg ggcctttttg gagttccata accttgtcga tttactccga

26461 aaacaaggta ccgaggttcg ggtccgttgg tgccctgggc accagggaat cccgggaaac

26521 gaccgggctg acgagctggc caaggccggc tccgccggac cgccggaccc agacccgagg

26581 gctcagcaaa ccacgtatag cggtgccggc acggtcctca gagccattct ttcgaatata

26641 gagaaagact ggtggcgtaa agaactctgt gaacggtccc ccgcatatag ggaatggaaa

26701 ttccaataca caccgagaaa ggagcccgag gaactgcgtt tgccaagacc cctactgggc

26761 cattatttgg ccatgaggac cggccacggc gatttcaaag cctaccatga ccgtttcaac

26821 caccaggatg caaacacctc gtgtgcctgg tgctggaagc ggacctcccc tgaacacccg

26881 gtgcactgcc gcttttcgcg ggcggtgtgg agaaactggc cgtggcctga caacgaccgg

26941 ccggtcgggc cgccagaccg cgcccaacgc cgcaaattct tccagacaag cctcgggcaa

27001 ccgacaagct ttcaggcgtt ttcgatagcc accaactact tcagcgcccg ccccagagcg

27061 gcccgccagc gccccgcgcg ccacgaacgc actttacgcc tagggacacc gatcgtaaac

27121 gactcgaact ctgacgagga atagacttac tgccttttca cccacacttc acggcacaag

27181 ccgtcagacg aaccctccgt gcaccttagg cacggggtcg cgtcagtgaa ctaaccccct

27241 aagcaggacc gggcccgaac ccggtcaggc acgatccgcc tctgccctcc ttgttttccc

27301 cctgtgtaaa taaagaagat agaacgcgcg ccgagatacc cctcgggagg ttgctaacgg

27361 ccggctaaca agccgggccg agcccggcgt taactaatac tactactact actactacta

27421 caacatcaat ttagacgaca cgctaatttt attcacaaac tcatatcgac ttgagccgaa

27481 tgcgaaggcc cgagccgtgg tgtccgttgt ccgattaatc cctgggtccg gaattacaaa

| 21 | AACU02000649 |
| --- | --- |

22381 gctggccgta ataaagacca gccaggatat ttgcgtacac gtgccaaata gacatgcctg

22441 ccatttgatt accaataacg tcgatggcgt tggccagata atgtagacct ggtgttacgt

22501 ctatatttct cctcggccct gacgtggaag cagaactttg gaaggaggct cgcctgctgc

22561 gttttgggtg cgacggcaca ttgttttgaa aggccgggga acctcgagaa ccgcatggct

22621 gagagttaag ggagcctatg ctatgcatct caccgaaggt gggaacgata tcgggaatac

22681 gatccttgtg gagacagatc tcgcctagtg caagaatgca gagaaccaca gcagtgctag

22741 tagtagtagt agtagtagta gtagtagtag tattagttaa cgccgggctc ggcccggctt

22801 gttagccggc cgttagcaac ctcccgaggg gtatctcggc gcgcgttcta tcttctttat

22861 ttacacaggg ggaaaacaag gagggcagag gcggatcgtg cctgaccggg ttcgggcccg

22921 gtcctgctta gggggttagt tcactgacgc gaccccgtgc ctaaggtgca cggagggttc

22981 gtctgacggc ttgtgccgtg aagtgtgggt gaaaaggcag taagtctatt cctcgtcaga

23041 gttcgagtcg tttacgatcg gtgtccctag gcgtaaagtg cgttcgtggc gcgcggggcg

23101 ctggcgggcc gctctggggc gggcgctgaa gtagttggtg gctatcgaaa acgcctgaaa

23161 gcttgtcggt tgcccgaggc ttgtctggaa gaatttgcgg cgttgggcgc ggtctggcgg

23221 cccgaccggc cggtcgttgt caggccacgg ccagtttctc cacaccgccc gcgaatagcg

23281 gcagtgcacc gggtgcttag gggaggtccg cttccagcac caggcacacg aggtgtttgc

23341 atcctggtgg ttgaaacggt catggtaggc tttgaaatcg ccgtggccgg tcctcatggc

23401 caaataatgg cccagtaggg gtcttggcaa acgcagttcc tcgggctcct ttctcggtgt

23461 gtattggaat ttccattccc tatatgcggg ggaccgttca cagagttctt tacgccacca

23521 gtccttcttt atattcgaaa gaatggctct gaggaccgtg ccggcaccgc tatacgtggt

23581 ttgctgagcc ctcgggtctg ggtccggcgg tccggcggag ccggccttgg ccagctcgtc

23641 agcccggtcg tttcccggga ttccctggtg cccagggcac caacggaccc gaacctcggt

23701 accttgtttt cggagtagat cgacaaggtt atggaactcc agaaaggccc attgggacga

23761 acgcggcgcg tcgcctctaa gaccccaaat aaccgaggtg ctgtccacac aaatccaaat

23821 ccgggcgcct ggctgggcct tggctgccgc ctgtagccct tttagggcgc caatcgcctc

23881 ggcgtcgaaa acgtggcttt ccggcgtaat agatgcggag cctgcggcaa attccaggcc

23941 cgccctaaaa gcggcccatc cgtaccctat ttggacgcag ttgttttcgt gtttttccga

24001 accatccgta tataccacga tatcgtcagg tgataccatg tccagccatt cgataaagcg

24061 gcgggccgct tcctctttcg ggactcctcc ggtggggtca gtgcgagaat ccggggcatt

24121 gcgaggtgcg cgcaactcta atctccggat ccgcgggaga agttgtgcag ccgtttgcag

24181 ggtcgtggcc gaatggcccg agttgcgggc acgaggtgtt tcacgcaggc ggctgacaag

24241 ggggtgcccc ttgtccgcga gccttagtcg ggcgccgtat ttcaggcggg tgtaggccag

24301 cgcgacgcgg gccgagggta atcctgcgtc cctgagaaca gtggacgagg gggtggtttt

24361 atacgccggg aggattgccc gtgccgctaa aaccagcggt ttgttcaatg ctttgaggaa

24421 tcctctttgc ctgtgcgctg ggttgtacca tgcctcggct gcgtaggtgg ccgaggaacc

24481 cacgcatgcc accgctgcct tgcgaagtgc agccgcaggc ggtccgtatc ttactgcccc

24541 aaagctcttg aggtgggcag cgaccgccat tgctttggca gccctttcgg ccacgtggcg

24601 gcgcccgtct agccgtccct tgcatttcct acagtaaact atagtcacag ggttggacca

24661 gaccctctaa cggacaggcc agctagagcc ctcctagtcg ctggataccc agcctgccac

24721 gtaaatacaa ggtttgcacc ttaagcctac cgcatccaca aaaaggcctc ttggtagacc

24781 gacgcgcgca tccgtcctct ccggctaaaa tagcgtctct tccgcctaac atgccgacga

24841 catcatctgc cccggtgtac gtccctagtg cgagtagaaa gcttcgaaaa gccggcgaca

24901 ccccgtacat gtccatccac agcttagtcc cccgttggtt cctgggctag cccagcgcca

24961 caatgtggac atgaatgcaa tcgagttctt cccgtcctcc agacccggcg cagattccca

25021 gtgcgagtga gaagcatggg gcgcctcagt cacactattc caccacggcc caccgtccac

25081 ggggtgtacc aagggtgcga accaaggccg tgcccacgtt tcttgcttcc cgttacccaa

25141 agccgtctcc agcaatgggc cgaccacgtc ttgctgtcgg gaccctttgg agactgccgc

25201 gccggcacag tgcttccggt ccacgtacaa ctaaccaacc cccgtaacgg cactacctcc

25261 tcgctgcagg caatcccaga aggtgtgagg gggtgagagg gtgccctgtc gattcgaaga

25321 accgtcgggt ggttctcagc ctaatttccc tttcttttaa accgtattgt ctagccttcc

25381 ggggaaggac gcctgccttt ccggctggca aagttttttt gtgccacgct ggctcgcatc

25441 gcgagattgg gccttgtctt gtgcgcctct atgcgggtgg cgcgccgaac cgccgatcgg

25501 actcttcctt caccgcgatc agtggaaaac tcgtcaaagg ggagatctgt cgattataca

25561 tgggtgttgc gaggaaaggc gttccgtgta gtgacggtgc ttagggcgac aggttgaaga

25621 gtttttcgtg tgtgcgcgcc tgttagggct acaggcggaa gtcccgcgca aacgttaaca

25681 gcatggtccg tgcgcccata ctgaaaattt tgtgtacggg agagagttga aacttgaacc

25741 tcatgtacgg tgcatgaccc tgctttacga atacgtgtct ttttggccct tcgtcgtttg

25801 ctaagcgcct cattggcctt ccgaaggctg cggttctctg cttcaagaag cgtggtccga

25861 tgagcaattg attctaatcc tttcgccaat tgcttcactg cctcaaaaat cggtgaagga

| 22 | AACU02000525 |
| --- | --- |

31081 gcattattca gaggaatcgc gaatgcgaga ggagaaagaa tcatggaaat tactaatcac

31141 tgtcaaacat tacccttcca aaatagctgc cggtggggga ataagtagta gtagtagtag

31201 tagtattagt taacgccggg ctcggcccgg cttgttagcc ggccgttagc aacctcccga

31261 ggggtatctc ggcgcgcgtt ctatcttctt tatttacaca gggggaaaac aaggagggca

31321 gaggcggatc gtgcctgacc gggttcgggc ccggtcctgc ttagggggtt agttcactga

31381 cgcgaccccg tgcctaaggt gcacggaggg ttcgtctgac ggcttgtgcc gtgaagtgtg

31441 ggtgaaaagg cagtaagtct attcctcgtc agagttcgag tcgtttacga tcggtgtccc

31501 taggcgtaaa gtgcgttcgt ggcgcgcggg gcgctggcgg gccgctctgg ggcgggcgct

31561 gaagtagttg gtggctatcg aaaacgcctc aaagcttttc ggttgcccga agcttgtctg

31621 gaagaatttc cagcgttggg cgcgatttgg cggcccggcc ggccggtcgt tattaggcca

31681 cggccagttt ctccacaccg cccgcgaata gcggcagtgc accgggtgct caggggaggt

31741 ccgcttccag caccaggcac acgaggtgtt tgcatcctgg tggttgaaac ggtcatggta

31801 ggccttgaaa tcgccgtggc cggtcctcat ggccaaataa tggcccagta ggggtcttgg

31861 caaacgcagt tcctcgggct cctttctcgg tgtgtattgg aatttccatt ccctatatgc

31921 gggggaccgt tcacagagtt ctttacgcca ccagtccttc tctatattgg aaagaatggc

31981 tctgaggacc gtgccggcac cgctatacgt ggtttgctga gccctcgggt ctgggtccgg

32041 cggtccggcg gagccggcct tggccagctc gtcagcccgg tcgtttcccg ggattccctg

32101 gtgcccaggg caccaacgga cccgaacctc ggtaccttgt tttcggagta gatcgacaag

32161 gtcatggaac tccagaaagg cccattggga cgaacgcggc gcgtcgcctc taagacccca

32221 aataaccgag gtgctgtcca cacaaatcca gatccgggcg cctggctggg ccttggctgc

32281 cgcctgtagc ccttttaggg cgccaatcgc ctcggcgtcg aaaacgtggc tttccggcgt

32341 aatagatgcg gagcctgcgg caaattccag gcccgcccta aaagcggccc atccgtaccc

32401 tatttggacg cagttgtttt cgtgtttttc cgaaccatcc gtatatacca cgatatcgtc

32461 aggtgatacc atgtccagcc attcgataaa gcggcgggcc gcttcctctt tcgggactcc

32521 tccggtgggg tcagtgcgag aatccggggc attgcgaggt gcgcgcaact ctaatctccg

32581 gatccgcggg agaagttgtg cagccgtttg cagggtcgtg gccgaatggc ccgagttgcg

32641 ggcacgaggt gtttcacgca ggcggctgaa caagggggtg ccccttgtcc gcgagcctta

32701 gtcgggcgcc gtattttcag ggcgggtgta ggccagcgcg acgtcgggcc gagggtagtc

32761 ctgcgtccct gagaacagtg gacgaggggg tggttttata cgccgggagg attgcccgtg

32821 ccgctaaaac cagcggtttg ttcaatgctt tgaggagtcc tctttgcttg tgcgctgggt

32881 tgtaccatgc ctcggctgcg taggtggccg aggaacccac gcatgccacc gctgccttgc

32941 gaagtgcagc cgcaggcggt ccgtatctta ctgccccaaa gctcttgagg tgggcagcga

33001 ccgccattgc tttggcagcc ctttcggcca cgtggcggcg cccgtctagc cgtcggctga

33061 accaaaaacc aagccaacgc atgcccgggt agc cttgcat ttcctacagt aaactatagt

33121 cacagggttg gaccagaccc tctaacggac aggccagcta gagccctcct agtcgctgga

33181 tacccagcct gccacgtaaa tacaaggttt gcaccttaag cctaccgcat ccacaaaaag

33241 gcctcttggt agaccgacgc gcgcatccgt cctctccggc taaaatagcg tctcttccgc

33301 ctaacatgcc gacgacatca tctgccccgg tgtacgtccc tagtgcgagt agaaagcttc

33361 gaaaagccgg cgacaccccg tacatgtcca tccacagctt agtcccccgt tggttcctgg

33421 gctagcccag cgccacaatg atggacatga atgcaatcga gttcttcccg tcctccagac

33481 ccggcgcaga ttcccagtgc gagtgagaag catggggcgc ctcagtcaca ctattccacc

33541 acggcccacc gtccacgggg tgtaccaagg gtgcgaacca aggccgtgcc cacgtttctt

33601 gcttcccgtt acccaaagcc gtctccagca atgggccgac cacgtcttgc tgtcgggacc

33661 ctttggagac tgccgcgccg gcacagtgct tccggtccac gtacaactaa ccaacccccg

33721 taacggcact acctcctcgc tgcaggcaat cccagaaggt gtgagggggt gagagggtgc

33781 cctgtcgatt cgaagaaccg tcgggtggtt ctcagcctaa tttccctttc ttttaaaccg

33841 tattgtctag ccttccgggg aaggacgcct gcctttccgg ctggcaaagt tttttgtgcc

33901 acgctggctc gcatcgcgag attgggcctt gtcttgtgcg cctctatgcg ggtggcgcgc

33961 cgagccgccg atcggactct tccttcaccg cgatcagtgg aaaactcgtc aaaggggaga

34021 tctgtcgatt atacatgggt gttgcgagga aaggcgttcc gtgtagtgac ggtgcttagg

34081 gcgacaggtt gaagagtttt tcgtgtgtgc gcgcctgtta gggctacagg cggaagtccc

34141 gcgcaaacgt taacagcatg gtccgtgcgc ccatactgaa aattttgtgt acgggagaga

34201 gttgagaggg agtgcagaag ggtgacaaga attgggaatc cgcgtcgtgg gtagcctgct

34261 ggatggcacg cgtagtaggc tcgtgacttg cgtggcagca ggcggttgct ggtgtgatcg

| 23 | AACU02000324 |
| --- | --- |

34141 cttgatcgta acatgtcact tttggattta cttttttctg attttaattc tccattttcc

34201 aatgaagcaa tatactgaaa aacagcttat atctgcaatt aacgacgtca ataatggcaa

34261 tccaattgca aaaacctccc gaaaatgggg aatacctagg tc**tacacttc** **aaagtc**caac

34321 tctctcccgt acacaaaatt ttcagtatgg gcgcacggac catgctgtta acgtttgcgc

34381 gggacttccg cctgtagccc taacaggcgc gcacacacga aaaacttttc aacctgtcgc

34441 cctaagcacc gtcactacac ggaacgcctt tcctcgcaac acccatgtat aatcgacaga

34501 tctccccttt gacgagtttt ccactgatcg cggtgaagga agagtccgat cggcggctcg

34561 gcgcgccacc cgcatagagg cgcacaagac aaggcccaat ctcgcgatgc gagccagcgt

34621 ggcacaaaaa actttgccag ccggaaaggc aggcgtcctt ccccggaagg ctagacaata

34681 cggtttaaaa gaaagggaaa ttaggctgag aaccacccga cggttcttcg aatcgacagg

34741 gcaccctctc accccctcac accttctggg attgcctgca gcgaggaggt agtgccgtta

34801 cgggggttgg ttagttgtac gtggaccgga agcactgtgc cggcgcggca gtctccaaag

34861 ggtcccgaca gcaagacgtg gtcggcccat tgctggagac ggctttgggt aacgggaagc

34921 aagaaacgtg ggcacggcct tggttcgcac ccttggtaca ccccgtggac ggtgggccgt

34981 ggtggaatag tgtgactgag gcgccccatg cttctcactc gcactgggaa tctgcgccgg

35041 gtctggagga cgggaagaac tcgattgcat tcatgtccac attgtggcgc tgggctagcc

35101 caggaaccaa cgggggacta agctgtggat ggacatgtac ggggtgtcgc cggcttttcg

35161 aagctttcta ctcgcactag ggacgtacac cggggcagat gatgtcgtcg gcatgttagg

35221 cggaagagac gctattttag ccggagagga cggatgcgcg cgtcggtcta ccaagaggcc

35281 tttttgtgga tgcggtaggc ttaaggtgca aaccttgtat ttacgtggca ggctgggtat

35341 ccagcgacta ggagggctct agctggcctg tccgttagag ggtctggtcc aaccctgtga

35401 ctatagttta ctgtaggaaa tgcaagg(WEIRD,str+)tcc ggggccgagc gctacccggg catgcgttgg

35461 cttggttttt ggttcagccg acggctagac gggcgccgcc acgtggccga aagggctgcc

35521 aaagcaatgg cggtcgctgc ccacctcaag agctttgggg cagtaagata cggaccgcct

35581 gcggctgcac ttcgcaaggc agcggtggca tgcgtgggtt cctcggccac ctacgcagcc

35641 gaggcatggt acaacccagc gcgcaagcaa agaggactcc tcaaagcatt gaacaaaccg

35701 ctggttttag cggcacgggc aatcctcccg gcgtataaaa ccaccccctc gtccactgtt

35761 ctcagggacg caggactacc ctcggcccgc gtcgcgctgg cctacacccg cctgaaatac

35821 ggcgcccgac taaggctcgc ggacaagggg cacccccttg tcagccgcct gcgtgaaaca

35881 cctcgtgccc gcaactcggg ccattcggcc acgaccctgc aaacggctgc acaacttctc

35941 ccgcggatcc ggagactaga gttgcgcgca cctcgcaatg ccccggattc tcgcactgac

36001 cccaccggag gagtcccgaa agaggaagcg gcccgccgct ttatcgaatg gctggacatg

36061 gtatcacctg acgatatcgt ggtatatacg gatggttcgg aaaaacacga aaacaactgc

36121 gtccaaatag ggtacggatg ggccgctttt agggcgggcc tggaatttgc cgcaggctcc

36181 gcatctatta cgccggaaag ccacgttttc gacgccgagg cgattggcgc cctaaaaggg

36241 ctacaggcgg cagccaaggc ccagccaggc gcccggatct ggatttgtgt ggacagcacc

36301 tcggttattt ggggtcttag aggcgacgcg ccgcgttcgt cccaatgggc ctttctggag

36361 ttccataacc ttgtcgatct actccgaaaa caaggtaccg aggttcgggt ccgttggtgc

36421 cctgggcacc agggaatccc gggaaacgac cgggctgacg agctggccaa ggccggctcc

36481 gccggaccgc cggacccaga cccgagggct cagcaaacca cgtatagcgg tgccggcacg

36541 gtcctcagag ccattctttc gaatatagag aaggactggt ggcgtaaaga actctgtgaa

36601 cggtcccccg catataggga atggaaattc caatacacac cgagaaagga gcccgaggaa

36661 ctgcgtttgc caagacccct actgggccat tatttggcca tgaggaccgg ccacggcgat

36721 ttcaaagcct accatgaccg tttcaaccac caggatgcaa acacctcgtg tgcctggtgc

36781 tggaagcgga cctcccctga acacccggtg cactgccgct tttcgcgggc ggtgtggaga

36841 aactggccgt ggcctgacaa cgaccggccg gtcgggccgc cagaccgcgc ccaacgccgc

36901 aaattcttcc agacaagcct cgggcaaccg acaagctttc aggcgttttc gatagccacc

36961 aactacttca gcgcccgccc cagagcggcc cgccagcgcc ccgcgcgcca cgaacgcact

37021 ttacgcctag ggacaccgat cgtaaacgac tcgaactctg acgaggaata gacttactgc

37081 cttttcaccc acacttcacg gcacaagccg tcagacgaac cctccgtgca ccttaggcac

37141 ggggtcgcgt cagtgaacta accccctaag caggaccggg cccgaacccg gtcaggcacg

37201 atccgcctct gccctccttg ttttccccct gtgtaaataa agaagataga acgcgcgccg

37261 agatacccct cgggaggttg ctaacggccg gctaacaagc cgggccgagc ccggcgttaa

37321 ctaatactac tactactact ac(MGL, 5428-7320, str+)tac**tacac** **ttcaaagtc**g acttaaaggt tctcaacctt

37381 ataaaaaagc acaaagccct tttcaaaggc tttccacgga acaggaaaag catttggctg

| 24 | AACU02000514 |
| --- | --- |

14641 gcaagccatg tgtaggtctg tctgtacatg actctgaacc aaaaggacac gagtctgacc

14701 aactctctcc cgtacacaaa attttcagta tgggcgcacg gaccatgctg ttaacgtttg

14761 cgcgggactt ccgcctgtag ccctaacagg cgcgcacaca cgaaaaactc ttcaacctgt

14821 cgccctaagc accgtcacta cacggaacgc ctttcctcgc aacacccatg tataatcgac

14881 agatctcccc tttgacgagt tttccactga tcgcggtgaa ggaagagtcc gatcggcggc

14941 tcggcgcgcc acccgcatag aggcgcacaa gacaaggccc aatctcgcga tgcgagccag

15001 cgtggcacaa aaaactttgc cagccggaaa ggcaggcgtc cttccccgga aggctagaca

15061 atacggttta aaagaaaggg aaattaggct gagaaccacc cgacggttct tcgaatcgac

15121 agggcaccct ctcaccccct cacaccttct gggattgcct gcagcgagga ggtagtgccg

15181 ttacgggggt tggttagttg tacgtggacc ggaagcactg tgccggcgcg gcagtctcca

15241 aagggtcccg acagcaagac gtggtcggcc cattgctgga gacggctttg ggtaacggga

15301 agcaagaaac gtgggcacgg ccttggttcg cacccttggt acaccccgtg gacggtgggc

15361 cgtggtggaa tagtgtgact gaggcgcccc atgcttctca ctcgcactgg gaatctgcgc

15421 cgggtctgga ggacgggaag aactcgattg cattcatgtc cacattgtgg cgctgggcta

15481 gcccaggaac caacggggga ctaagctgtg gatggacatg tacggggtgt cgccggcttt

15541 tcgaagcttt ctactcgcac tagggacgta caccggggca gatgatgtcg tcggcatgtt

15601 aggcggaaga gacgctattt tagccggaga ggacggatgc gcgcgtcggt ctaccaagag

15661 gcctttttgt ggatgcggta ggcttaaggt gcaaaccttg tatttacgtg gcaggctggg

15721 tatccagcga ctaggagggc tctagctggc ctgtccgtta gagggtctgg tccaaccctg

15781 tgactatagt ttactgtagg aaatgcaagg(WEIRD, str+) atatttggcg tatctcgtca aaaacggtac

15841 gaagtggcgg ttggcatacg cagacgacgt gcttacatgg aaatcgtcac cctcgttgga

15901 ggaaaacgta cgttggctgg aagataaact ccgggatatg cacgaaattg cggcggaaga

15961 gaagatccat tttgcagcgg aaaagacaga ggtgatccat atcactaaga aaaggcacgg

16021 tcgcaacccg gaaatccgga ttaatggtag aacggttacc ccggtccaac taccgggcgg

16081 tcgacgcgga caaagcgcct ccggggccga gcgctacccg ggcatgcgtt ggcttggttt

16141 ttggttcagc cgacggctag acgggcgccg ccacgtggcc gaaagggctg ccaaagcaat

16201 ggcggtcgct gcccacctca agagctttgg ggcagtaaga tacggaccgc ctgcggctgc

16261 acttcgcaag gcagcggtgg catgcgtggg ttcctcggcc acctacgcag ccgaggcatg

16321 gtacaaccca gcgcacaagc aaagaggact cctcaaagca ttgaacaaac cgctggtttt

16381 agcggcacgg gcaatcctcc cggcgtataa aaccaccccc tcgtccactg ttctcaggga

16441 cgcaggacta ccctcggccc gcgtcgcgct ggcctacacc cgcctgaaat acggcgcccg

16501 actaaggctc gcggacaagg ggcaccccct tgtcagccgc ctgcgtgaaa cacctcgtgc

16561 ccgcaactcg ggccattcgg ccacgaccct gcaaacggct gcacaacttc tcccgcggat

16621 ccggagacta gagttgcgcg cacctcgcaa tgccccggat tctcgcactg accccaccgg

16681 aggagtcccg aaagaggaag cggcccgccg ctttatcgaa tggctggaca tggtatcacc

16741 tgacgatatc gtggtatata cggatggttc ggaaaaacac gaaaacaact gcgtccaaat

16801 agggtacgga tgggccgctt ttagggcggg cctggaattt gccgcaggct ccgcatctat

16861 tacgccggaa agccacgttt tcgacgccga ggcgattggc gccctaaaag ggctacaggc

16921 ggcagccaag gcccagccag gcgcccggat ctggatttgt gtggacagca cctcggttat

16981 ttggggtctt agaggcgacg cgccgcgttc gtcccaatgg gcctttttgg agttccataa

17041 ccttatcgat ctactccgaa aacaaggcac cgaggttcgg gtccgttggt gccctgggca

17101 ccagggaatc ccgggaaacg accgggctga cgagctggcc aaggccggct ctgccggacc

17161 gccggaccca gacccgaggg ctcagcaaac cacgtatagc ggtgccggca cggtcctcag

17221 agccattctt tcgaatatag agaaggactg gtggcgtaaa gaactttgtg aacggtcccc

17281 cgcatatagg gaatggaaat tccaatacac accgagaaag gagcccgagg aactgcgttt

17341 gccaagaccc ctactgggcc attacttggc catgaggacc ggccacggcg atttcaaagc

17401 ctaccatgac cgtttcaacc accaggatgc aaacacctcg tgtgcctggt gctggaagcg

17461 gacctcccct gaacacccgg tgcactgccg cttttcgcgg gcggtgtgga gaaactggcc

17521 gtggcctgac aacgaccggc cggtcgggcc gccagaccgc gcccaacgcc gcaaattctt

17581 ccagacaagc ctcgggcaac cgaaaagctt tgaggcgttt tcgatagcca ccaactactt

17641 cagcgcccgc cccagagcgg cccgccagcg ccccgcgcgc cacgaacgca ctttacgcct

17701 agggacaccg atcgtaaacg actcgaactc tgacgaggaa tagacttact gccttttcac

17761 ccacacttca cggcacaagc cgtcagacga accctccgtg caccttaggc acggggtcgc

17821 gtcagtgaac taacccccta agcaggaccg ggcccgaacc cggtcaggca cgatccgcct

17881 ctgccctcct tgttttcccc ctgtgtaaat aaagaagata gaacgcgcgc cgagataccc

17941 ctcgggaggt tgctaacggc cggctaacaa gccgggccga gcccggcgtt aaataatact

18001 actactacta ctac(MGL, str+, 3774-5977)tacata acacgagtct gaccgtagag ttttgagccc aagagagaca

| 25 | AACU02000457 |
| --- | --- |

390901 atgttagtta gaacgtgccg tcccaaggct atcacctatg atgacttttg aaagaatacc

390961 ccaatttctt gctagcagtc aactctctcc cgtacacaaa attttcagta tgggcgcacg

391021 gaccatgctg ttaacgtttg cgcgggactt ccgcctgtag ccctaacagg cgcgcacaca

391081 cgaaaaactc ttcaacctgt cgccctaagc accgtcacta cacggaacgc ctttcctcgc

391141 aacacccatg tataatcgac agatctcccc tttgacgagt tttccactga tcgcggtgaa

391201 ggaagagtcc gatcggcggt tcggcgcgcc acccgcatag aggcgcacaa gacaaggccc

391261 aatctcgcga tgcgagccag cgtggcacaa aaaactttgc cagccggaaa ggcaggcgtc

391321 cttccccgga aggctagaca atacggttta aaagaaaggg aaattaggct gagaaccacc

391381 cgacggttct tcgaatcgac agggcaccct ctcaccccct cacaccttct gggattgcct

391441 gcagcgagga ggtagtgccg ttacgggggt tggttagttg tacgtggacc ggaagcactg

391501 tgccggcgcg gcagtctcca aagggtcccg acagcaagac gtggtcggcc cattgctgga

391561 gacggctttg ggtaacggga agcaagaaac gtgggcacgg ccttggttcg cacccttggt

391621 acaccccgtg gacggtgggc cgtggtggaa tagtgtgact gaggcgcccc atgcttctca

391681 ctcgcactgg gaatctgcgc cgggtctgga ggacgggaag aactcgattg cattcatgtc

391741 cacattgtgg cgctgggcta gcccaggaac caacggggga ctaagctgtg gatggacatg

391801 tacggggtgt cgccggcttt tcgaagcttt ctactcgcac tagggacgta caccggggca

391861 gatgatgtcg tcggcatgtt aggcggaaga gacgctattt tagccggaga ggacggatgc

391921 gcgcgtcggt ctaccaagag gcctttttgt ggatgcggta ggcttaaggt gcaaaccttg

391981 tatttacgtg gcaggctggg tatccagcga ctaggagggc tctagctggc ctgtccgtta

392041 gagggtctgg tccaaccctg tgactatagt ttactgtagg aaatgcaagg(WEIRD, str+) tgggccatac

392101 acgacaacgg gctccttagc tgcacccacg gcggtgccct acccaaacga tcggcgacgg

392161 atctggtttg tgccctcgcc cacgatatcg agcaggctct agctcgcggg ggaggaagtc

392221 acccttgtcg catgcgacgt ccaaggcgcc ttcgacgccc tcctccatag gcgattagat

392281 acggaaaatg cggagcctca ggtttagtaa aatgctcctc aggttcgtga ttaacttttt

392341 aagcggccgc caggcccgag tccggctgga gggtacgacc acgggcttta ggcggcttgg

392401 gtgcggcacc ccgcaagggt ctcccctttc cccgatccta tatatgctat atttggcgta

392461 tctcgtcaaa aacggtacga agtggcggtt ggcatacgca gacgacgtgc ttacatggaa

392521 atcgtcaccc tcgttggagg aaaacgtacg ttggctggaa gataaactcc gggatatgca

392581 cgaaattgcg gcggaagaga agatccattt tgcagcggaa aagacagagg tgatccatat

392641 cactaagaaa aggcacggtc gcaacccgga aatccggatt aatggtagaa cggttacccc

392701 ggtccaacta ccgggcggtc gacgcggaca aagcgcctcc ggggccgagc gctacccggg

392761 catgcgttgg cttggttttt ggttcagccg acggctagac gggcgccgcc acgtggccga

392821 aagggctgcc aaagcaatgg cggtcgctgc tcacctcaaa ggctttgggg cagtaagata

392881 cggaccgcct gcggctgcac ttcgcaaggc agcggtggca tgcgtgggtt cctcggccac

392941 ctacgcagcc gaggcatggt acaacccagc gcacaagcaa agaggactcc tcaaagcatt

393001 gaacaaaccg ctggttttag cggcacgggc aatcctcccg gcgtataaaa ccgaccccct

393061 cgtccactgt tctcagggac gcaggactac cctcggcccg cgtcgcgctg gcctacaccc

393121 gcctgaaata cggcgcccga ctaaggttcg cggacaaggg gcaccccctt gtcagccgcc

393181 tgcgtgaaac acctcgtgcc cgcaactcgg gccattcggc cacgaccctg caaacggctg

393241 cacaacttct cccgcggatc cggagactag agttgcgcgc acctcgcaat gccccggatt

393301 ctcgcactga ccccaccgga ggagtcccga aagaggaagc ggcccgccgc tttatcgaat

393361 ggctggacat ggtatcacct gacgatatcg tggtatatac ggatggttcg gaaaaacacg

393421 aaaacaactg cgtccaaata gggtacggat gggccgcttt tagggcgggc ctggaatttg

393481 ccgcaggctc cgcatctatt acgccggaaa gccacgtttt cgacgccgag gcgattggcg

393541 ccctaaaagg gctacaggcg gcagccaagg cccagccagg cgcccggatc tggatttgtg

393601 tggacagcac ctcggttatt tggggtctta gaggcgacgc gccgcgttcg tcccaatggg

393661 cctttctgga gttccatgac cttgtcgatc tactccgaaa acaaggtacc gaggttcggg

393721 tccgttggtg ccctgggcac cagggaatcc cgggaaacga ccgggctgac gagctggcca

393781 aggccggctc cgccggaccg ccggacccag acccgagggc tcagcaaacc acgtatagcg

393841 gtgccggcac ggtcctcaga gccattcttt ccaatataga gaaggactgg tggcgtaaag

393901 aactctgtga acggtccccc gcatataggg aatggaaatt ccaatacaca ccgaaaaagg

393961 agcccgagga actgcgtttg ccaagacccc tactgggcca ttatttggcc atgaggaccg

394021 gccacggcga tttcaaggcc taccatgacc gtttcaacca ccaggatgca aacacctcgt

394081 gtgcctggtg ctggaagcgg acctcccctg agcacccggt gcactgccgc tattcgcggg

394141 cggtgtggag aaactggccg tggcctaata acgaccggcc ggccgggccg ccaaatcgcg

394201 cccaacgctg gaaattcttc cagacaagct tcgggcaacc gaaaagcttt gaggcgtttt

394261 cgatagccac caactacttc agcgcccgcc ccagagctgc ccgccagcgc cccgcgcgcc

394321 acgaacgcac tttacgccta gggacaccga tcgtaaacga ctcgaactca gacgaggaat

394381 agacttactg ccttttcacc cacacttcac ggcacaagcc gtcagacgaa ccctccgtgc

394441 accttaggca cggggtcgcg tcagtgaact atccccctaa gcaggaccgg gcccgaaccc

394501 ggtcaggcac gatccgcctc tgccctcctt gttttccccc tgtgtaaata aagaagatag

394561 aacgcgcgcc gagatacccc tcgggaggtt gctaacggcc ggctaacaag ccgggccgag

394621 cccggcgtta aataatacta ctactactac tac(MGL, str+, 3418-5977)at**aaatg** **ttt**gaaacaa gccgagacaa

| 26 | AACU02000708 |
| --- | --- |

16501 ccgaacgaat tcttcaagcc gccggagaga caaaaggcct tggaaaacgt tggataactc

16561 gttttttggc tcgttatcca atccttaaaa cccaaaggcc ccgtcgaata gataacgccc

16621 gggttaatgg cgctactacg gaggtaatta aatcttggtg gctttatatt acgaacccgg

16681 ttattaacgc tattagtagt agtagtagta gtattagtta acgccgggct cggcccggct

16741 tgttagccgg ccgttagcaa cctcccgagg ggtatctcgg cgcgcgttct atcttcttta

16801 tttacacagg gggaaaacaa ggagggcaga ggcggatcgt gcctgaccgg gttcgggccc

16861 ggtcctgctt agggggttag ttcactgacg cgaccccgtg cctaaggtgc acggagggtt

16921 cgtctgacgg cttgtgccgt gaagtgtggg tgaaaaggca gtaagtctat tcctcgtcag

16981 agttcgagtc gtttacgatc ggtgtcccta ggcgtaaagt gcgttcgtgg cgcgcggggc

17041 gctggcgggc cgctctgggg cgggcgctga agtagttggt ggctatcgaa aacgcctcaa

17101 agcttttcgg ttgcccgagg cttgtctgga agaatttgcg gcgttgggcg cggtctggcg

17161 gcccgaccgg ccgctcgtta tcaagccacg gccagtttct ccacaccgcc cgcgaatagc

17221 ggcagtgcac cgggtgctca ggggaggtcc gcttccagca ccaggcacac gaggtgtttg

17281 catcctggtg gttgaaacgg tcatggtagg ctttgaaatc gccgtggccg gtcctcatgg

17341 ccaaataatg gcccagtagg ggtcttggca aacgcagttc ctcgggctcc tttctcggtg

17401 tgtattggaa tttccattcc ctatatgcgg gggaccgttc acagagttct ttacgccacc

17461 agtccttctc tatattcgaa agaatggctc tgaggaccgt gccggcaccg ctatacgtgg

17521 tttgctgagc cctcgggtct gggtccggcg gtccggcgga gccggccttg gccagctcgt

17581 cagcccggtc gtttcccggg attccctggt gcccagggca ccaacggacc cgaacctcgg

17641 taccttgttt tcggagtaga tcgacaaggt tatggaactc cagaaaggcc cattgggacg

17701 aacgcggcgc gtcgcctcta agaccccaaa taaccgaggt gctgtccaca caaatccaaa

17761 tccgggcgcc tggctgggcc ttggctgccg cctgtagccc ttttagggcg ccaatcgcct

17821 cggcgtcgaa aacgtggctt tccggcgtaa tagatgcgga gcctgcggca aattccaggc

17881 ccgccctaaa agcggcccat ccgtacccta tttggacgca gttgttttcg tgtttttccg

17941 aaccatccgt atataccacg atatcgtcag gtgataccat gtccagccat tcgataaagc

18001 ggcgggccgc ttcctctttc gggactcctc cggtggggtc agtgcgagaa tccggggcat

18061 tgcgaggtgc gcgcaactct agtctccgga tccgcgggag aagttgtgca gccgtttgca

18121 gggtcgtggc cgaatggccc gagttgcggg cacgaggtgt ttcacgcagg cggctgacaa

18181 gggggtgccc cttgtccgcg agccttagtc gggcgccgta tttcaggcgg gtgtaggcca

18241 gcgcgacgcg ggccgagggt agtcctgcgt ccctgagaac agtggacgag ggggtggttt

18301 tatacgccgg gaggattgcc cgtgccgcta aaaccagcgg tttgttcaat gctttgagga

18361 gtcctctttg cttgtgcgct gggttgtacc atgcctcggc tgcgtaggtg gccgaggaac

18421 ccacgcatgc caccgctgcc ttgcgaagtg cagccgcagg cggtccgtat cttactgccc

18481 caaagctctt gaggtgggca gcgaccgcca ttgctttggc agccctttcg gccacgtggc

18541 ggcgcccgtc tagccgtcgg ctgaaccaaa aaccaagcca acgcatgccc gggtagcgct

18601 cggccccgga ggcgctttgt ccgcgtcgac cgcccggtag ttggaccggg gtaaccgttc

18661 taccattaat ccggatttcc gggttgcgac cgtgcctttt cttagtaata tggatcacct

18721 ctgtcttttc cgctgcaaaa tggatcttct cttccgccgc aatttcgtgc atatcccgga

18781 gtttatcttc cagccaacgt acgttttcct ccaacgaggg tgacgatttc catgtaagca

18841 cgtcgtctgc gtatgccaac cgccacttcg taccgttttt gacgagatac gccaaatata

18901 gcatatatag gatcggggaa aggggagacc cttgcggggt gccgcaccca agccgcctaa

18961 agcccgtggt cgtaccctcc agccggactc gggcctggcg gccgcttaaa aagttaatca

19021 cgaacctgag gagcatttta ctaaacccga ggctccgcat tttccgtatt aatcgcctat

19081 ggaggagggc gtcgaaggcg ccttggacgt cgcatgcgac aagggtaact tcctccccgc

19141 gagctagagc ctgctcgata tcgtgggcga gggcacaaac cagatccgtc gccgatcgtt

19201 tgggtagggc accgccgtgg gtgcagctaa ggagcccgtt gtcgtgtatg gcccaagcta

19261 tccggcgtgc aacgagcctc tcgaggcctt ttccgatgca ggagaggagg gctataggcc

19321 gccaggatcg aacgctgctc cggtctttct tccccgtttt cggtagcatc gcgacttcgg

19381 ccttcttcca aggcgctggg aaatgtccga gttcca(MGL, str-, 3257-5977)cctt gcatttccta cagtaaacta

19441 tagtcacagg gttggaccag accctctaac ggacaggcca gctagagccc tcctagtcgc

19501 tggataccca gcctgccacg taaatacaag gtttgcacct taagcctacc gcatccacaa

19561 aaaggcctct tggtagaccg acgcgcgcat ccgtcctctc cggctaaaat agcgtctctt

19621 ccgcctaaca tgccgacgac atcatctgcc ccggtgtacg tccctagtgc gagtagaaag

19681 cttcgaaaag ccggcgacac cccgtacatg tccatccaca gcttagtccc ccgttggttc

19741 ctgggctagc ccagcgccac aatgtggaca tgaatgcaat cgagttcttc ccgtcctcca

19801 gacccggcgc agattcccag tgcgagtgag aagcatgggg cgcctcagtc acactattcc

19861 accacggccc accgtccacg gggtgtacca agggtgcgaa ccaaggccgt gcccacgttt

19921 cttgcttccc gttacccaaa gccgtctcca gcaatgggcc gaccacgtct tgctgtcggg

19981 accctttgga gactgccgcg ccggcacagt gtttccggtc cacgtacaac taaccaaccc

20041 ccgtaacggc actacctcct cgctgcaggc aatcccagaa ggtgtgaggg ggtgagaggg

20101 tgccctgtcg attcgaagaa ccgtcgggtg gttctcagcc taatttccct ttcttttaaa

20161 ccgtattgtc tagccttccg gggaaggacg cctgcctttc cggctggcaa agttttttgt

20221 gccacgctgg ctcgcatcgc gagattgggc cttgtcttgt gcgcctctat gcgggtggcg

20281 cgccgaaccg ccgatcggac tcttccttca ccgcgatcag tggaaaactc gtcaaagggg

20341 aggtctgtcg attatacatg ggtgttgcga ggaaaggcgt tccgtgtagt gacggtgctt

20401 agggcgacag gttgaagagt ttttcgtgtg tgcgcgcctg ttagggctac aggcggaagt

20461 cccgcgcaaa cgttaacagc atggtccgtg cgcccatact gaaaattttg tgtacgggag

20521 agagttg(WEIRD, str-)tcg atatcgtggg cgagggcaca aaccagatcc gtcgccgatc gtttgggtag

20581 ggcaccgccg tgggtgcagc taaggagccc gttgtcgtgt atggcccaag ctatccggcg

20641 tgcaacgagc ctctcgaggc cttttccgat gcaggagagg agggctatag gccgccagga

20701 tcgaacgctg ctccggtctt tcttccccgt tttcggtagc atcgcgactt cggccttctt

| 27 | AACU02000560 |
| --- | --- |

15121 tggagcagag cccaagtgcc tcaaaggcaa cctaccaaaa cgtccccgtc cgcgccccgg

15181 tctttaaccg cccacgaatc atcaacaagt catacgaggc actggtagac accgcatctg

15241 aatgctccga gtccgaagat ccgggctcac agtcgctgtc gctgcgaggc ggtggcgacc

15301 tcgaccgcga gcaacccccg cggtccgcac tcgactttga cctgctcaag gcccggctga

15361 agcgactgtg cacaaagcag ccggacccgg agttggaggc aagggtcaag agactggagg

15421 agtgtagtcg gcaacaggct ggcgtctggg aagaagagac cgaagaggtg atagtagtag

15481 tagtagtagt agtagtagta ttagttaacg ccgggctcgg cccggcttgt tagccggccg

15541 ttagcaacct cccgaggggt atctcggcgc gcgttctatc ttctttattt acacaggggg

15601 aaaacaagga gggcagaggc ggatcgtgcc tgaccgggtt cgggcccggt cctgcttagg

15661 gggttagttc actgacgcga ccccgtgcct aaggtgcacg gagggttcgt ctgacggctt

15721 gtgccgtgaa gtgtgggtga aaaggcagta agtctattcc tcgtcagagt tcgagtcgtt

15781 tacgatcggt gtccctaggc gtaaagtgcg ttcgtggcgc gcggggcgct ggcgggccgc

15841 tctggggcgg gcgctgaagt agttggtggc tatcgaaaac gcctcaaagc ttttcggttg

15901 cccgaagctt gtctggaaga atttccagcg ttgggcgcga tttggcggcc cggccggccg

15961 gtcgttatta ggccacggcc agtttctcca caccgcccgc gaatagcggc agtgcaccgg

16021 gtgctcaggg gaggtccgct tccagcacca ggcacacgag gtgtttgcat cctggtggtt

16081 gaaacggtca tggtaggcct tgaaatcgcc gtggccggtc ctcatggcca aataatggcc

16141 cagtaggggt cttggcaaac gcagttcctc gggctccttt ctcggtgtgt attggaattt

16201 ccattcccta tatgcggggg accgttcaca gagttcttta cgccaccagt ccttctctat

16261 attcgaaaga atggctctga ggaccgtgcc ggcaccgcta tacgtggttt gctgagccct

16321 cgggtctggg tccggcggtc cggcggagcc ggccttggcc agctcgtcag cccggtcgtt

16381 tcccgggatt ccctggtgcc cagggcacca acggacccga acctcggtac cttgttttcg

16441 gagtagatcg acaaggttat ggaactccag aaaggcccat tgggacgaac gcggcgcgtc

16501 gcctctaaga ccccaaataa ccgaggtgct gtccacacaa atccaaatcc gggcgcctgg

16561 ctgggccttg gctgccgcct gtagcccttt tagggcgcca atcgcctcgg cgtcgaaaac

16621 gtggctttcc ggcgtaatag atgcggagcc tgcggcaaat tccaggcccg ccctaaaagc

16681 ggcccatccg taccctattt ggacgcagtt gttttcgtgt ttttccgaac catccgtata

16741 taccacgata tcgtcaggtg ataccatgtc cagccattcg ataaagcggc gggccgcttc

16801 ctctttcggg actcctccgg tggggtcagt gcgagaatcc ggggcattgc gaggtgcgcg

16861 caactctagt ctccggatcc gcgggagaag ttgtgcagcc gtttgcaggg tcgtggccga

16921 atggcccgag ttgcgggcac gaggtgtttc acgcaggcgg ctgacaaggg ggtgcccctt

16981 gtccgcgagc cttagtcggg cgccgtattt caggcgggtg taggccagcg cgacgcgggc

17041 cgagggtagt cctgcgtccc tgagaacagt ggacgagggg gtggttttat acgccgggag

17101 gattgcccgt gccgctaaaa ccagcggttt gttcaatgct ttgaggagtc ctctttgctt

17161 gtgcgctggg ttgtaccatg cctcggctgc gtaggtggcc gaggaaccca cgcatgccac

17221 cgctgccttg cgaagtgcag ccgcaggcgg tccgtatctt actgccccaa agctcttgag

17281 gtgggcagcg accgccattg ctttggcagc cctttcggcc acgtggcggc gcccgtctag

17341 ccgtcggctg aaccaaaaac caagccaacg catgcccggg tagcgctcgg ccccggaggc

17401 gctttgtccg cgtcgaccgc ccggtagttg gaccggggta accgttctac cattaatccg

17461 gatttccggg ttgcgaccgt gccttttctt agtgatatgg atcacctctg tcttttccgc

17521 tgcaaaatgg atcttctctt ccgccgcaat ttcgtgcata tcccggagtt tatcttccag

17581 ccaacgtacg ttttcctcca acgagggtga cgatttccat gtaagcacgt cgtctgcgta

17641 tgccaaccgc cacttcgtac cgtttttgac gagatacgcc aaatataaca tatataggat

17701 cggggaaagg ggagaccctt gcggggtgcc gcacccaagc cgcctaaagc ccgtggtcgt

17761 accctccagc cggactcggg cctggcggcc gcttaaaaag ttaatcacga acctgaggag

17821 cattttacta aacccgaggc tccgcatttt ccgtattaat cgcctatgga ggagggcgtc

17881 gaaggcgcct tggacgtcgc atgcgacaag ggtgacttcc tccccgcgag ctagagcctg

17941 ctcgatatcg tgggcgaggg cacaaaccag atccgtcgcc gatcgtttgg gtagggcacc

18001 gccgtgggtg cagctaagga gcccgttgtc gtgtatggcc caagctatcc ggcgtgcaac

18061 gagcctctcg aggccttttc cgatgcagga gaggagggct ataggccgcc aggatcgaac

18121 gctgctccgg tctttcttcc ccgttttcgg tagcatcgcg acttcggcct tcttccaagg

18181 cgctgggaaa tgtccgagtt ccaggcaacg ttggtagagc ctgcgcaccg gctcggccaa

18241 cgaagcccat ccggctttta gtagccggac ggttattccg tcgatgcccg gggacgtgct

18301 cgtaacccca atgcaggagc gctccgcctc ttccgcacta acctgggtgt cccaagggat

18361 tttagcctcg tccggcgacc aggcctccaa ggggtctccc tgcaggtcat cctccgccga

18421 aaatcggcca agcacctccc gttgcagtgc ttccgctttt gctaaaggct cgctcacttg

18481 ctcgccgtta ataaccagcg gcggggaccg gaggcgtggt ccggctccca gccagcccac

18541 catgttccac aaattcttat cgtcgcgcag ttggtcgatc cggtgcctcc agtactcccg

18601 cttcgcggcc cgcacccctt tcgtatacct tgcatttcct acagtaaact atagtcacag

18661 ggttggacca gaccctctaa cggacaggcc agctagagcc ctcctagtcg ctggataccc

18721 agcctgccac gtaaatacaa ggtttgcacc ttaagcctac cgcatccaca aaaaggcctc

18781 ttggtagacc gacgcgcgca tccgtcctct ccggctaaaa tagcgtctct tccgcctaac

18841 atgccgacga catcatctgc cccggtgtac gtccctagtg cgagtagaaa gcttcgaaaa

18901 gccggcgaca ccccgtacat gtccatccac agcttagtcc cccgttggtt cctgggctag

18961 cccagcgcca caatgtggac atgaatgcaa tcgagttctt cccgtcctcc agacccggcg

19021 cagattccca gtgcgagtga gaagcatggg gcgcctcagt cacactattc caccacggcc

19081 caccgtccac ggggtgtacc aagggtgcga accaaggccg tgcccacgtt tcttgcttcc

19141 cgttacccaa agccgtctcc agcaatgggc cgaccacgtc ttgctgtcgg gaccctttgg

19201 agactgccgc gccgg

| 28 | AACU02000591 |
| --- | --- |

4021 ttgagaacga ttataacttt accccagtta ttaagaaccc cagttaccca attaaacgct

4081 cagttgcttc aacccactgt actttaccat aagaacaggt tacccgatac cttgatcgta

4141 acagtgttct tactaccttg aggacaggcg ttaaatatag atacctggca atgcaaagtt

4201 agcagagaac tttgttttgc agattgaggg aaaagtgact gggttctgat gcggtacctt

4261 gcattagcct gaccaagtcc ctgacgggtg cacccttttg tgtaatgttt gcgacctagt

4321 gctcccttgg ggtttgagat ggttttttgg ggacagaggt caactctctc ccgtacacaa

4381 aattttcagt atgggcgcac ggaccatgct gttaacgttt gcgcgggact tccgcctgta

4441 gccctaacag gcgcgcacac acgaaaaact cttcaacctg tcgccctaag caccgtcact

4501 acacggaacg cctttcctcg caacacccat gtataatcga caagatctcc cctttgacga

4561 gttttccact gatcgcggtg aaggaagagt ccgatcggcg gctcggcgcg ccacccgcat

4621 agaggcgcac aagacaaggc ccaatctcgc gatgcgagcc agcgtggcaa aaaaaaaaaa

4681 aactttgcca gccggaaagg caggcgtcct tccccggaag gctagataat acggtttaaa

4741 agaaagggaa attaggctga gaaccacccg acggttcttc gaatcgacag ggcaccctct

4801 caccccctca caccttctgg gattgcctgc aacgaggagg tagtgccgtt acgggggttg

4861 gttagttgta cgtggaccgg aagcactgtg ccggcgcggc agtctccaaa gggtcccgac

4921 agcaagacgt ggtcggccca ttgctggaga cggctttggg taacgggaag caagaaacgt

4981 gggcacggcc ttggttcgca cccttggtac accccgtgga cggtgggccg tggtggaata

5041 gtgtgactga ggcgccccat gcttctcact cgcactggga atctgcgccg ggtctggagg

5101 acgggaagaa ctcgattgca ttcatgtcca cattgtggcg ctgggctagc ccaggaacca

5161 acgggggact aagctgtgga tggacatgta cggggtgtcg ccggcttttc gaagctttct

5221 actcgcacta gggacgtaca ccggggcaga tgatgtcgtc ggcatgttag gcggaagaga

5281 cgctatttta gccggagagg acggatgcgc gcgtcggtct accaagaggc ctttttgtgg

5341 atgcggtagg cttaaggtgc aaaccttgta tttacgtggc aggctgggta tccagcgact

5401 aggagggctc tagctggcct gtccgttaga gggtctggtc caaccctgtg actatagttt

5461 actgtaggaa atgcaagg(WEIRD, str+)ac cgcagggcca aaatacgcgg gacatactct ggctcgaaat

5521 taacggaata ttgtttgtta acgtatatag ggctccgggc actgaagccg cgctggaaat

5581 ggtatgcaat accgtgccaa atggacccac ggtgctaggc ggcgatttta acgtagcggc

5641 tgctgcatac cagccgggcc gcgcaaacgc acgcggaggg gaccaactga cggcatgggc

5701 gcaagcccag gggatgagtt ttacaggaaa tatcggcgtg cccacc(MGL, str+, 2127-2394)aact gccagcggct

5761 ctggtccgaa ttccagcggg ttaaacggag cgctgtaaat agggcagacg cctctgccga

5821 ggaaaaagcc tatacgaaag gggtgcgggc cgcgaagcgg gagtactgga ggcaccggat

5881 cgaccaactg cgcgacgata aggatttgtg gaatatggtg ggctggctgg gagccggacc

5941 acgcctccgg tccccgccgc tggttattaa cggcgagcaa gtgagcgagc ctttagcaaa

6001 agcggaagca ctgcaacggg aggtgcttgg ccgattttcg gcggaggatg acctgcaggg

6061 agaccccttg gaggcctggt cgccggacga ggctaaaatc ccttgggaca cccaggttag

6121 tgcggaagag gcggagcgct cctgcattgg ggttacgagc acgtccccgg gcatcgacgg

6181 aatgaccgtc cggctactaa aagccggatg ggcttcgttg gccgagccgg tgcgcaggct

6241 ctaccaacgt tgcctggaac tcggacattt cccagcgcct tggaagaagg ccgaagtcgc

6301 gatgctaccg aaaacgggga agaaagaccg gagcagcgtt cgatcctggc ggcctatagc

6361 cctcctctcc tgcatcggaa aaggcctcga gaggctcgtt gcacgccgga tagcttgggc

6421 catacacgac aacgggctcc ttagctgcac ccacggcggt gccctaccca aacgatcggc

6481 gacggatctg gtttgtgccc tcgcccacga tatcgaacag gctttagctc gcggggagga

6541 agttaccctt gtcgcatgcg acgtccaagg cgccttcgac gccctcctcc atgggcgatt

6601 aatacggaaa atgcggagcc ttgggtttag taaaatgctc ctcaggttcg tgattaactt

6661 tctaaacgac cgccaggccc gagtccggct ggagggtacg accacgggct ttaggcggct

6721 tgagtgcggc accccgcaag ggtctcccct ttccccgatc ctatatatgc tatatttggc

6781 gtatctcgtc aaaaacggta cgaagtggcg gttggcatac gcagacgacg tgtttacatg

6841 gaaatcgtca ccctcgttgg aggaaaacgt acgttggctg gaagataaac tccgggatat

6901 gcacgaaatt gcggcggaag agaagatcca ttttgcagcg gaaaagacag aggtgatcca

6961 tatcactaaa aaaaggcacg gtcgcaaccc ggaaatccgg attaatggta gaacggttac

7021 cccggtccaa ctaccgggcg gtcgacgcgg acaaagcgcc tccggggccg agcgctaccc

7081 gggcatgcgt tggcttggtt tttggttcag ccgacggtta gacgggcgcc gccacgtggc

7141 cgaaagggct gccaaagcaa tggcggtcgc tgcccacctc aagagctttg gggcagtaag

7201 atacggaccg cctgcggctg cacttcgcaa ggcagcggtg gcatgcgtgg gttcctcggc

7261 cacctacgca gccgaggcat ggtacaaccc agcgcacaag caaagaggac tcctcaaagc

7321 attgaacaaa ccgctggttt tagcggcacg ggcaatcctc ccggcgtata aaaccacccc

7381 ctcgtccact gttctcaggg acgcaggact accctcggcc cgcgtcgcgc tggcctacac

7441 ccgcctgaaa tacggcgccc gactaaggct cgcggacaag gggcaccccc ttgtcagccg

7501 cctgcgtgaa acacctcgtg cccgcaactc gggccattcg gccacgaccc tgcaaacggc

7561 tgcacaactt ctcccgcgga tccggagact agagttgcgc gcacctcgca atgccccgga

7621 ttctcgcact gaccccaccg gaggagtccc aaaagaggaa gcggcccgcc gctttatcga

7681 atggctggac atggtatcac ctaacgatat cgtggtatat acggatggtt cggaaaaaca

7741 cgaaaataat tgcgtccaaa tagggtacgg atgggccgct tttagggcgg gcctggaatt

7801 tgccgcaggc tccgcatcta ttacgccgga aagccacgtt ttcgacgccg aggcgattgg

7861 cgccctaaaa gggctacagg cggcagccaa ggcccagcca ggcgcccgga tctggatttg

7921 tgtggacagc acctcggtta tttggggtct tagaggcgac gcgccgcgtt cgtcccaatg

7981 ggcctttctg gagttccata accttgtcga tctactccga aaacaaggta ccgaggttcg

8041 ggtccgttgg tgccctgggc accagggaat cccgggaaac gaccgggctg acgagctggc

8101 caaggccggc tccgccggac cgccggaccc agacccgagg gctcagcaaa ccacgtatag

8161 cggtgccggc acggtcctca gagccattct ttcgagtata gagaaggact ggtggcgtaa

8221 agaactctgt gaacggtccc ccgcatatag ggaatggaaa ttccaataca caccgagaaa

8281 ggagcccgag gaactgcgtt tgccaagacc cctactgggc cattatttgg ccatgaggac

8341 cggccacggc gatttcaaag cctaccatga ccgtttcaac caccaggatg caaacacctc

8401 gtgtgcctgg tgctggaagc ggacctcccc tgaacacccg gtgcactgcc gcttttcgcg

8461 ggcggtgtgg agaaactggc cgtggcctga caacgaccgg ccggtcgggc cgccagaccg

8521 cgcccaacgc cgcaaattct tccagacaag cctcgggcaa ccgacaagct ttcaggcgtt

8581 ttcgatagcc accaactact tcagcgcccg ccccagagcg gcccgccagc gccccgcgcg

8641 ccacgaacgc actttacgcc tagggacacc gatcgtaaac gactcgaact ctgacgagga

8701 atagacttac tgccttttca cccacacttc acggcacaag ccgtcagacg aaccctccgt

8761 gcaccttagg cacggggtcg cgtcagtgaa ctaaccccct aagcaggacc gggcccgaac

8821 ccggtcaggc acgatccgcc tctgccctcc ttgttttccc cctgtgtaaa taaagaagat

8881 agaacgcgcg ccgagatacc cctcgggagg ttgctaacgg ccggctaaca agccgggccg

8941 agcccggcgt taactaatac tactactact actac(MGL, str+, 2749-5977)tacta cttggggaca gaggtctgtc

9001 aaagtcggag cttgtcccag gggcagtcgt acaaactcgc tctctacctt ggagattgag

9061 gtgcggacag ccggccaggc gaggacctct tggaaaaccc acttgtgaac ctttgtctcg

9121 gccaattttg cagaccccca gaccttgctg ggccccgggc atggccgttg tttgatttaa

| 29 | AACU02000397 |
| --- | --- |

7681 ctaaactcgt acaaataaag tcgctatgaa ctgcaatttc atcaacacat cactggcaca

7741 catgtttgaa ctttgaaata accaccaacc gagtctaaac tcgatttcca acaagagata

7801 gcctagcata ctgggtataa cgaaacagta atacacacaa aaatgttacc tgcaaatcta

7861 acaaagcagg aaagcagtgc tttgctttgc ataatgtgcg agatgtcaaa tcaacacggc

7921 caaacaagat agtatatatt cagctctttg tctttgatgt gccctggctt gatgtacgtt

7981 ggcttaacgc ctccaaacgc ttgtactctt cacaatagac cttgcaatat tttttaaatc

8041 ccggggtaaa atcaagccca gcctctcgta aggtttcttt gcaaagtagt agtagtagta

8101 ttagttaacg ccgggctcgg cccggcttgt tagccggccg ttagcaacct cccgaggggt

8161 atctcggcgc gcgttctatc ttctttattt acacaggggg aaaacaagga gggcagaggc

8221 ggatcgtgcc tgaccgggtt cgggcccggt cctgcttagg gggttagttc actgacgcga

8281 ccccgtgcct aaggtgcacg gagggttcgt ctgacggctt gtgccgtgaa gtgtgggtga

8341 aaaggcagta agtctattcc tcgtcagagt tcgagtcgtt tacgatcggt gtccctaggc

8401 gtaaagtgcg ttcgtggcgc gcggggcgct ggcgggccgc tctggggcgg gcgctgaagt

8461 agttggtggc tatcgaaaac gcctcaaagc ttttcggttg cccgaagctt gtctggaaga

8521 atttccagcg ttgggcgcga tttggcggcc cggccggccg gtcgttatta ggccacggcc

8581 agtttctcca caccgcccgc gaatagcggc agtgcaccgg gtgctcaggg gaggtccgct

8641 tccagcacca ggcacacgag gtgtttgcat cctggtggtt gaaacggtca tggtaggcct

8701 tgaaatcgcc gtggccggtc ctcatggcca aataatggcc cagtaggggt cttggcaaac

8761 gcagttcctc gggctccttt ctcggtgtgt attggaattt ccattcccta tatgcggggg

8821 accgttcaca gagttcttta cgccaccagt ccttctctat attcgaaaga atggctctga

8881 ggaccgtgcc ggcaccgcta tacgtggttt gctgagccct cgggtctggg tccggcggtc

8941 cggcggagcc ggccttggcc agctcgtcag cccggtcgtt tcccgggatt ccctggtgcc

9001 cagggcacca acggacccga acctcggtac cttgttttcg gagtagatcg acaaggttat

9061 ggaactccag aaaggcccat tgggacgaac gcggcgcgtc gcctctaaga ccccaaataa

9121 ccgaggtgct gtccacacaa atccagatcc ggggcgcctg gctgggcctt ggctgccgcc

9181 tgtagccctt ttagggcgcc aatcgcctcg gcgtcgaaaa cgtggctttc cggcgtaata

9241 gatgcggagc ctgcggcaaa ttccaggccc gccctaaaag cggcccatcc gtaccctatt

9301 tggacgcagt tgttttcgtg tttttccgaa ccatccgtat ataccacgat atcgtcaggt

9361 gataccatgt ccagccattc gataaagcgg cgggccgctt cctctttcgg gactcctccg

9421 gtggggtcag tgcgagaatc cggggcattg cgaggtgcgc gcaactctag tctccgggat

9481 ccgcgggaga agttgtgcag ccgttttgca gggtcgtggc cgaatggccc gagttgcggg

9541 cacgaggtgt ttcacgcagg cggcctgaca agggggtgcc cccttgtccg cgagccttag

9601 tcggggcgcc gtatttccag gcgggtgtag gccagcgcga cgcgggccga gggtagtcct

9661 gccgtccctg agaacagtgg acgagggggt ggttttatac gccgggagga ttgcccgtgc

9721 cgctaaaacc agcggtttgt tcaatgcttt gaggagtcct ctttgcttgt gcgctgggtt

9781 gtaccatgcc tcggctgcgt aggtggccga ggaacccacg catgccaccg ctgccttgcg

9841 aagtgcagcc gcaggcggtc cgtatcttac tgccccaaag ctcttgaggt gggcagcgac

9901 cgccattgct ttggcagccc tttcggccac gtggcggcgc ccgtctagcc gtcggctgaa

9961 ccaaaaacca agccaacgca tgcccgggta gcgctcggcc ccggaggcgc tttgtccgcg

10021 tcgaccgccc ggtagttgga ccggggtaac cgttctacca ttaatccgga tttccgggtt

10081 gcgaccgtgc cttttcttag tgatatggat cacctctgtc ttttccgctg caaaatggat

10141 cttctcttcc gccgcaattt cgtgcatatc ccggagttta ttttccagcc aacgtacgtt

10201 ttcctccaac gagggtgacg atttccatgt aagcacgtcg tctgcgtatg ccaaccgcca

10261 cttcgtaccg tttttgacga gatacgccaa atataacata tataggatcg gggaaagggg

10321 agacccttgc ggggtgccgc acccaagccg cctaaagccc gtggtcgtac cctccagccg

10381 gactcgggcc tggcggccgc ttaaaaagtt aatcacgaac ctgaggagca ttttactaaa

10441 cccgaggctc cgcattttcc gtattaatcg cctatggagg aggggcgtcg aaggcgcctt

10501 ggacgtcgca tgcgacaagg gtgacttcct ccccgcgagc tagagcctgc tcgatatcgt

10561 gggcgagggc acaaaccaga tccgtcgccg atcgtttggg tagggcaccg ccgtgggtgc

10621 agctaaggag cccgttgtcg tgtatggccc aagctatccg gcgtgcaacg agcctctcga

10681 ggccttttcc gatgcaggag aggagggcta taggccgcca ggatcgaacg ctgctccggt

10741 ctttcttccc cgttttcggt agcatcgcga cttcggcctt cttccaaggc gctgggaaat

10801 gtccgagttc caggcaacgt tggtagagcc tgcgcaccgg ctcggccaac gaaacccatc

10861 cggcttttag tagccggacg gtcattccgt cgatgcccgg ggacgtgctc gtaaccccaa

10921 tgcaggagcg ctccgcctct tccgcactaa cctgggtgtc ccaagggatt ttagcctcgt

10981 ccggcgacca ggcctccaag gggtctccct gcaggtcatc ctccgccgaa aatcggccaa

11041 gcacctcccg ttgcagtgct tccgcttttg ctaaaggctc gctcacttgc tcgccgttaa

11101 taaccagcgg cggggaccgg aggcgtggtc cggctcccag ccagcccacc atgttccaca

11161 aatccttatc gtcgcgcagt tggtcgatcc ggtgcctcca gtactcccgc ttcgcggccc

11221 gcaccccttt cgtataggct ttttcctcgg cagaggcgtc tgccctattt acagcgctcc

11281 gtttaacccg ctggaattcg gaccagagcc gctggcagtt ttcggtccac cagggagccg

11341 agctgtccct ttttcccgcc ggcgtaccca cgacccacgt cacttgctcc cataaagttg

11401 taaattcagc tacccacgcg tccaatgcgt agccatcggc cgcggatccc gggtccggca

11461 tgtcttgcat gccaaaagca agtagctccg cgaactttgg taaccggtcg tccctaaccg

11521 aaacgttaat cgccctccgg gcggggggcg ccgcgcgtcc gcagcgtggt ataaagggat

11581 tcgtggtcgg agcctgtgta aagactgctg tcaaccacag ttattgtgct ggggaggttg

11641 gaaaagacca tatccagtaa acccccgtcg cggtgggtgg gcacgccgat atttcctgta

11701 aaactcatcc cctgggcttg cgcccatgcc gtcagttggt cccctccgcg tgcgtttgcg

11761 cggcccggct ggtatgcagc agccgctacg ttaaaatcgc cgcctagcac cgtgggtcca

11821 tttggcacgg tattgcatac catttccagc gcggcttcag tgcccggagc cctatatacg

11881 ttaacaaaca atattccgtt aatttcgagc cagagtatgt cccgcgtatt ttggccctgc

11941 ggtagccgtc gttgtgcggc ccttagggct gcccccttct taacgtaggt gagcacccgg

12001 ggccggagcc cagtcatggc ttcgtaagtg ttggcgtgcc attcgtccac tggcgcaaaa

12061 acatcatagc ccgggtgcgt ttgtgtagtc gtatttagcc cgcaccatgg ctcttg(MGL, 1954-5977, str-)cctt

12121 gcatttccta cagtaaacta tagtcacagg gttggaccag accctctaac ggacaggcca

12181 gctagagccc tcctagtcgc tggataccca gcctgccacg taaatacaag gtttgcacct

12241 taaacctacc gcatccacaa aaaggcctct tggtaaccga cgcgcgcatc cgtcctctcc

12301 ggctaaaata gcgtctcttc cgcctaacat gccgacgaca tcatctgccc cggtgtacgt

12361 ccctagtgcg agtagaaagc ttcgaaaagc cggcgacacc ccgtacatgt ccatccacag

12421 cttagtcccc cgttggttcc tgggctagcc cagcgccaca atgtggacat gaatgcaatc

12481 gagttcttcc cgtcctccag acccggcgca gattcccagt gcgagtgaga agcatggggc

12541 gcctcagtca cactattcca ccacggccca ccgtccacgg ggtgtaccaa gggtgcgaac

12601 caaggccgtg cccacgtttc ttgcttcccg ttacccaaag ccgtctccag caatgggccg

12661 accacgtctt gctgtcggga ccctttggag actgccgcgc cggcacagtg cttccggtcc

12721 acgtacaact aaccaacccc cgtaacggca ctacctcctc gctgcaggca atcccagaag

12781 gtgtgagggg gtgagagggt gccctgtcga ttcgaagaac cgtcgggtgg ttctcagcct

12841 aatttccctt tcttttaaac cgtattgtct agccttccgg ggaaggacgc ctgcctatcc

12901 ggctggcaaa gttttttgtg ccacgctggc tcgcatcgcg agattgggcc ttgtcttgtg

12961 cgcctctatg cgggtggcgc gccgagccgc cgatcggact cttccttcac cgcgatcagt

13021 ggaaaactcg tcaaagggga gatctgtcga ttatacatgg gtgttgcgag gaaaggcgtt

13081 ccgtgtagtg acggtgctta gggcgacagg ttgaagagtt tttcgtgtgt gcgcgcctgt

13141 tagggctaca ggcggaagtc ccgcgcaaac gttaacagca tggtccgtgc gcccatactg

13201 aaaattttgt gtacgggaga gagttg(WEIRD, str-)catg acggagtatc aggaacagcg tgtccttcag

13261 tgagatattt ttcaggaaaa tagcggtgta accggcggaa accaataaga acgaggagag

13321 gctgccggta aaagatgggc ggtggattga gtaaaagtaa tgcatagcat acgcggtcgc

13381 ttgacaactg ggattctctg gactattacg aatcaaaact cgattgccag acctaaaagc

13441 tctttttgct tttctttttt catcacccag ctgagtctat taacagcaca tggaattgcg

| 30 | AACU02000227 |
| --- | --- |

3901 attgtcatat tttccccctt ggtattcgaa gggctttgtc ccttttgctg gcctttttac

3961 tcatttattt ttctgttttc cttttttatc ttgattttgc ggatctgtga atttgaagac

4021 gtagccacgt gcaaacagat tcatatatgt acaggtttca gtgtggcaaa ataagtttat

4081 ttttgacatt gggatttgtt tttaactttg ggatattttt gtggtgctgg gcaacggatg

4141 caatgtatgg acatagcttg taggaggtgg ccaacttggt agagttgagt tgctcctttt

4201 gccccttcca acacaccacc cgtatacagt ttatctttat tgcaaatatg caactctctc

4261 ccgtacacaa aattttcagt atgggcgcac gtaccatgct gttaacgttt gcgcgggact

4321 tccgcctgta gccctaacag gcgcgcacac acgaaaaact ctgcaacctg tcgccctaag

4381 caccgtcact acacggaacg cctttcctcg caacacccat gtataatcga cagatctccc

4441 ctttgacgag ttttccactg atcgcggtga aggaagagtc cgatcggcgg ttcggcgcgc

4501 cacccgcata gaggcgcaca agacaaggcc caatctcgcg atgcgagcca gcgtggcaca

4561 aaaaactttg ccagccggaa aggcaggcgt ccttccccgg aaggctagac aatacggttt

4621 aaaagaaagg gaaattaggc tgagaaccac ccgacggttc ttcgaatcga cagggcaccc

4681 tctcaccccc tcacaccttc tgggattgcc tgcagcgagg aggtagtgcc gttacggggg

4741 ttggttagtt gtacgtggac cggaagcact gtgccggcgc aggcagtctc caaagggtcc

4801 cgacagcaag acgtggtcgg cccattgctg gagacggctt tgggtaacgg gaagcaagaa

4861 acgtgggcac ggccttggtt cgcacccttg gtacaccccg tggacggtgg gccgtggtgg

4921 aatagtgtga ctgaggcgcc ccatgcttct cactcgcact gggaatctgc gccgggtctg

4981 gaggacggga aaaactcgat tgcattcatg tccacattgt ggcgctgggc tagcccagga

5041 accaacgggg gactaagctg tggatggaca tgtacggggt gtcgccggct tttcgaagct

5101 ttctactcgc attagggacg tacaccgggg caaatgatgt cgtcggcatg ttaggcggaa

5161 gagacgctat tttagccgga gaggacggat gcgcgcgtcg gtctaccaag aggccttttt

5221 gtggatgcgg taggcttaag gtgcaaacct tgtatttacg tggcaggctg ggtatccagc

5281 gactaggagg gctctagctg gcctgtccgt tagagggtct ggtccaaccc tgtgactata

5341 atttactgta ggaaatgcaa gg(WEIRD, 1112bp)ctaagcac cttatcgcag cattcgcaaa cctcgagctc

5401 ccgatcgagc atttcaaaca aaagagcgcg gccatgcgag gcggcagggg cggacatccg

5461 taacttcctg caggttgaac aggaacgcgt gcacgacgaa atcgtttggg cagccgaaat

5521 ggaggaggac gcagcggctg ccgaaccttg ccccgctaat ggaatagact tggaagcaac

5581 ccgtcgatta atatgacgaa atactcgctc ggcaatatgc agcgggaatg cctcgacgta

5641 gtttgggcca acgtaggtaa aaggatgggt gtgcatctga gcctattgga gttgtgccac

5701 cagagaaagg tggacctggt taacgttcaa gagccatagt gcgggctaaa tacaactaca

5761 caaacgcacc cggggctatg atgtttttgc gccagtggac gaatggcacg ccaacactta

5821 cgaagccata actgggctcc ggccccgggt gctcacctac gtcaagaagg gggcagccct

5881 aagggccgca caacgacggc taccgcaggg ccaaaatacg cgggacatac tctggctcga

5941 aattaacgga atattgtttg ttaacgtata tagggctccg ggcactaaag ccgcgctgga

6001 aatggtatgc aataccgtgc caaatggacc cacggtgcta ggcggcgatt ttaacgtagc

6061 ggctgctgca taccagccgg gccgcgcaaa cgcacgcgga ggggaccaac tgacggcatg

6121 ggcgcaagcc caggggatga gttttacagg aaatatcggc gtgcccaccc accgcgacgg

6181 gggtttactg gatatagtct tttccaacct ccccagcaca ataactgtgg ttgacagcag

6241 tctttacaca ggctccgacc acgaatccct ttataccacg ctgcggacgc gcggcgcccc

6301 ccgcccggag gcgatcaacg tttcggttag ggacgaccgg ttaccaaagt tcgcggagct

6361 acttgctttt ggcatataag atatgccgga cccgggatcc gcggccgatg gctacgcatt

6421 ggacgcgtgg gtagctgaat ttacaacttt atgggagcaa gtgacgtggg tcgtgggtac

6481 gccggcggga aaaagggaca gctcggctcc ctggtggacc gaaaactgcc agcggctctg

6541 gtccgaattc cagcgggtta aacggagcgc tgtaaatagg gcagacgcct ctgccgagga

6601 aaaagcctat acgaaagggg tgcgggccgc gaagcgggag tactggaggc accggatcga

6661 ccaactgcgc gacgataaga atttgtggaa catggtgggc tggctgggag ccggaccacg

6721 cctccggtcc ccgccgctgg ttattaacgg cgagcaagtg agcgagcctt tagcaaaagc

6781 ggaagcactg caacgggagg tgcttggccg attttcggcg gaggataact tgcagggaga

6841 ccccttggag gcctggtcgg cggacgaggc taaaatccct tgggacaccc aggttagtgc

6901 ggaagaggcg gagcgctcct gcattggggt tacgagcacg tccccgggta tcgacggaat

6961 gaccgtccgg ctactaaaag ccggataagg tttcgttggc cgagccggtg cgcaggctct

7021 accaacgttg cctggaactc ggacatttcc cagcgccttg gaagaaggcc gaagtcgcga

7081 tgctaccgaa aacggggaag aaagaccgga gcagcgttcg atcctggcgg cctatagccc

7141 tcctctcctg catcggaaaa ggcctcgaga ggctcgttgc acgccggata gcttgggcca

7201 tacacgataa cgggctcctt aactgcaccc acggcggtgc cctacccaaa cgatcggcga

7261 cggatctggt ttgtgccctc gcccacgata tcgagcaggt tttagctcgc ggggaggaag

7321 ttacccttgt cgcatgcgac gtccaaggcg ccttcgacgc cctcctccat gggcgattaa

7381 tacggaaaat gcggagcctt gggtttagta aaatgctcct caggttcgtg attaactttc

7441 taaacgaccg cgaggcccga gtccggctgg agggtacgac cacgggcttt aggcggtttg

7501 ggtgcggcac cccgcaaggg tctccccttt ccccgatcct atatatgcta tatttggcgt

7561 atctcgtcaa aaacggtacg aagtggcggt tggcatacgc agacgacgtg cttacatgga

7621 aatcgtcacc ctcgttggag gaaaacgtac gttggctgga agataaactc cgggatatgc

7681 acgaaattgc ggcggaagag aagatccatt ttgcagcgga aaagacagag gtgatccata

7741 tcactaaaaa aaggcacggt cgcaacccgg aaatccggat taatggtaga acggttaccc

7801 cggtccaact accgggcggg tcgacgcgga caaagcgcct ccggggccga gcgctacccg

7861 ggcatgcgtt ggcttggttt ttggttcagc cgacggttag acgggcgccg ccacgtggcc

7921 gaaagggctg ccaaaacaat ggcggtcgct gctcacctta aaggctttgg ggcagtaaaa

7981 tacggaccgc ctgcggctgc acttcgcaag gcagcggtgg catgcgtggg ttcctcggcc

8041 acctacgcag ccgaggcatg gtacaaccca gcgcacaagc aaagaggact cctcaaaaca

8101 ttgaacaaac cgctggtttt agcggcacgg gcaatcctcc cggcgtataa aaccaccccc

8161 tcgtccactg ttctcaggga cgcaggacta ccctcggccc gcgtacgcgc tggcctacac

8221 ccgcctgaaa tacggcgccc gactaaggtt cgcggacaag gggcaccccc ttgttaaccg

8281 cctgcgtgaa acacctcgtg cccgcaactc gggccattcg gccacgaccc tgcaaacggc

8341 tgcacaactt ctcccgcgga tccggagatt agagttgcgc gcacctcgca atgccccgga

8401 ttctcgcact gaccccaccg gaggagtccc gaaagaggaa gcggcccgcc gctttatcga

8461 atggctggac atggtattac ctgacgatat cgtggtatat acggatggtt cggaaaaaca

8521 cgaaaacaac tgcgtccaaa tagggtacgg atgggccgct tttagggcgg gcctggaatt

8581 tgccgcaggc tccgcattta ttacgccgga aagccacgtt ttcgacgccg aggcgattgg

8641 cgccctaaaa gggctacagg cggcagccaa ggcccagcca ggcgcccgga tctggatttg

8701 tgtggacagc acctcggtta tttggggtct tagaggcgac gcgccgcgtt cgtcccaatg

8761 ggcctttctg aagttccata accttgtcga tctactccga aaacaaagta ccgaggttcg

8821 ggtccgttgg tgccctgggc accagggaat cccgggaaac gaccgggcta acgagctggc

8881 caaggccggc tccgccggac cgccggaccc agacccgagg gctcagcaaa ccacgtatag

8941 cggatgtccg gcacggtcct cagagccatt ctttcgaata tagagaagga ctggtggcgt

9001 aaagaactct gtacaacggt cccccgcata tagggaatgg aaattccaat acacaccgag

9061 aaaggagccc gaggaactgc gtttgccaag acccctactg ggccattatt tggccatgag

9121 gaccggccac ggcgatttca aagcctacca tgaccgtttc aaccaccagg atgcaaacac

9181 ctcgtgtgcc tggtgctgga agcggacctc ccctgaacac ccggtgcact gccgcttttc

9241 gcgggcggtg tggagaaact ggccgtggcc tgacaacgac cggccggtcg ggccgccaga

9301 ccgcgcccaa cgccgcaaat tcttccagac aagcctcggg caaccgacaa gctttcaggc

9361 gttttcgata gccaccaact acttcagcgc ccgccccaga gcggcccgcc agcgccccgc

9421 gcgccacgaa cgcactttac gcctagggac accgatcgta aacgactcga actctgacga

9481 ggaatagact tactgccttt tcacccacac ttcacggcac aagccgtcag acgaaccctc

9541 cgtgcacctt aggcacgggg tcgcgtcagt gaactaaccc cctaagcagg accgggcccg

9601 aacccggtca ggcacgatcc gcctctgccc tccttgtttt ccccctgtgt aaataaagaa

9661 gatagaacgc gcgccgagat acccctcggg aggttgctaa cggccggcta acaagccggg

9721 ccgagcccgg cgttaactaa tactactact actactac(MGL, 1589-7977, str+)tt gcgggcagtt ctttagagca

9781 gcctttgatg aaagatatat gttccaggta ggaattcaat taaatctgag ctgggctggt

9841 gtctaaatag gtagcgcgga tgctaatctc atgctgggcc aaatatagga agccattaca

9901 tcaacaatca ccataccaga agatgccaaa acatttgggt ttctggcctt ttttgcctac

| 31 | AACU02000381 |
| --- | --- |

26701 ccccgcaaca acctcgatca cttttaattt tcctattttg gtgatttcaa ctcgtggcaa

26761 caatgcgtgg tccgacaggg taaatatgtc ctggatgact gccttgcaaa ccgaaagttg

26821 caaccactgg ttgaagtcat tgaccatgcc tcgtccagca gcctggccgg caataccaca

26881 caaaggatcg attggagact cgagcttcca ggatctggaa caggtgggtt caagtcagcg

26941 ttggcacccc gagcagctct acagacaggc gggattacca tctcttgttg gcaatccaat

27001 gacaactgag gcacaagaag tgccaactgc gaacccgggg aaattctct**t** **taggggattc**

27061 **tta**caactct ctcccgtaca caaaattttc agtatgggcg cacggaccat gctgttaacg

27121 tttgcgcggg acttccgcct gtagccctaa caggcgcgca cacacgaaaa actcttcaac

27181 ctgtcgccct aagcaccgtc actacacgga acgcctttcc tcgcaacacc catgtataat

27241 cgacagacct cccctttgac gagttttcca ctgatcgcgg tgaaggaaga gtccgatcgg

27301 cggttcggcg cgccacccgc atagaggcgc acaagacaag gcccaatctc gcgatgcgag

27361 ccagcgtggc acaaaaaaac tttgccagcc ggaaaggcag gcgtccttcc ccggaaggct

27421 agacaatacg gtttaaaaga aagggaaatt aggctgagaa ccacccgacg gttcttcgaa

27481 tcgacagggc accctctcac cccctcacac cttctgggat tgcctgcagc gaggaggtag

27541 tgccgttacg ggggtttggt tagttgtacg tggaccggaa gcactgtgcc ggcgcggcag

27601 tctccaaagg gtcccgacag caagacgtgg tcggcccatt gctggagacg gctttgggta

27661 acgggaagca agaaacgtgg gcacggcctt ggttcgcacc cttggtacac cccgtggacg

27721 gtgggccgtg gtggaatagt gtgactgagg cgccccatgc ttctcactcg cactgggaat

27781 ctgcgccggg tctggaggac gggaagaact cgattgcatt catgtccaca ttgtggcgct

27841 gggctagccc aggaaccaac gggggactaa gctgtggatg gacatgtacg gggtgtcgcc

27901 ggcttttcga agctttctac tcgcactagg gacgtacacc ggggcagatg atgtcgtcgg

27961 catgttaggc ggaagagacg ctattttagc cggagaggac ggatgcgcgc gtcggtctac

28021 caagaggcct ttttgtggat gcggtaggtt taaggtgcaa accttgtatt tacgtggcag

28081 gctgggtatc cagcgactag gagggctcta gctggcctgt ccgttagagg gtctggtcca

28141 accctgtgac tatagtttac tgtaggaaat gcaaggagca gccagtcaac tggcatattt

28201 ccaaacatgg ttacgaccca catacggctg agggcacgtg gatagtgtcc ttccttcaac

28261 ccgtaaagcc ctttcagctt tttggagtgt cgagacgtgc gcggaaggtt gaaaaatcac

28321 gcaaaattac acaccaccac gacggttgcc agggatattg cgaaatacgt cgctgcgtac

28381 ggcaagcgcg ttgcggcata tgtggtgaag caaaacatgc gacagaggaa gagccgtgca

28441 aggcagcccc caaatgcgtt aattgccacg gccatttccc atccggacat gagaattgcc

28501 ctgcccgacc tttggtggta aatgggaagt ttgaacgacc ttcacagcgt aaacttaagg

28561 ggattcgcag aatcggacgt gccaaccgtg ccgcgattat caacgaccgg gccgaaacgg

28621 cccgccgaga gccagcacct gcaatcccgg tcctaagcac cttatcgcag cattcgcaaa

28681 cctcgagctc ccgatcgagc atttcaaaca aaagagcgcg gccatgcgag gcggcagggg

28741 cggacatccg taacttcctg caggttgaac aggaacgcgt gcacgacgaa atcgtttggg

28801 cagccgaaat ggaggaggac gcagcggctg ccgaaccttg ccccgctgat gaggatagac

28861 ttggaagcaa cccgtcgatt aatatgacga aatactcgct cggcaatatg cagcgggaat

28921 gcctcgacgt agtttgggcc atacgtaggt aaaaggatgg gtgtgcatct gagcctattg

28981 gagttgtgcc accagagaaa ggtggacctg gttaacgttt aagagccatg gtgcgggcta

29041 aatacgacta cacaaacgca cccggggcta tgatgttttt gcgccagtgg acgaatggca

29101 cgccaacact tacgaagcca tgactgggct ccggccccgg gtgctcacct acgtcaaaaa

29161 gggggcagcc ctaagggccg cacaacgacg gctaccgcag ggccaaaata cgcgggacat

29221 actctggctc gaaattaacg gaatactgtt tgttaacgta tatagggctc cgggcactga

29281 agccgcgctg gaaatggtat gcagataccg tgccaaatgg acccacggtg ctaggcggcg

29341 attttaacgt agcggctgct gcataccagc cgggccgcgc aaacgcacgc ggaggggacc

29401 aactgacggc atgggcgcaa acccagggga tgagttttac aggaaatatc ggcgtgccca

29461 cccaccgcga cgggggttta ctggatatgg tcttttccaa cctccccagc acaataactg

29521 tggttgacag cagtctttac acaggctccg accacgaatc cctttatacc acgctgcgga

29581 cgcgcggcgc cccccgcccg gaggcgatca acgtttcggt tagggacgac cggttaccaa

29641 agttcgcgga gctacttgct tttggcatgc aagacatgcc ggacccggga tccgcggccg

29701 atggctacgc attggacgcg tgggtagctg aatttacaac tttatgggag caagtgacgt

29761 gggtcgtggg tacgccggcg ggaaaaaggg acagctcggc tccctggtgg accgaaaact

29821 gccagcggct ctggtccgaa ttccagcggg ttaaacggag cgctgtaaat agggcagacg

29881 cctctgccga ggaaaaagcc tatacgaaag gggtgcgggc cgcgaagcgg gagtactgga

29941 ggcaccggat cgaccaactg cgcgacgata aggatttgtg gaacatggtg ggctggctgg

30001 gagccggacc acgcctcgcg gtccccgccg ctggttatta acggcgagca agtgagcgag

30061 cctttagcaa aagcggaagc actgcaacgg gaggtgcttg gccgattttc ggcggaggat

30121 gacctgcagg gagacccctt ggaggcctgg tcgccggacg aggctaaaat cccttgggac

30181 acccaggtta gtgcggaaga ggcggagcgc tcctgcattg gggttacgag cacgtccccg

30241 ggcatcgacg gaatgaccgt ccggctacta aaagccggat gggcttcgtt ggccgagccg

30301 gtgacgcagg ctctaccaac gttgcctgga actcggacat ttcccagcgc cttggaagaa

30361 ggccgaagtc gcgatgctac cgaaaacggg gaagaaagac cggagcagcg ttcgatcctg

30421 gcggcctata gccctcctct cctgcatcgg aaaaggcctc cgagaggctc gttgcacgcc

30481 ggatagcttg ggccatacac gacaacgggc tccttagctg cacccacggc ggtgccctac

30541 ccaaacgatc ggcgacggat ctggtttgtg ccctcgccca cgatatcgag caggctctag

30601 ctcgcgggga ggaagtcacc cttgtcgcat gcgacgtcca aggcgccttc gacgccctcc

30661 tccataggcg attaatacgg aaaatgcgga gcctcgggtt tagataaaat gctcctcagg

30721 ctcgtgatta actttttaag cggccgccag gcccgagtcc ggctggaggg tacgaccacg

30781 ggctttaggc ggcttgggtg cggcaccccg caaggagtct cccctttccc cgatcctata

30841 tatgctatat ttggcgtatc tcgtcaaaaa cggtacgaag tggcggttgg catacgcaga

30901 cgacgtgctt acatggaaat cgtcaccctc gttggaggaa aacgtacgtt ggctggaaga

30961 taagactccg ggatatgcac gaaattgcgg cggaagagaa gatccatttt gcagcggaaa

31021 agacagaggt gatccatatc actaagaaaa ggcacggtcg caacccggaa atccggatta

31081 atggtagaac ggttaccccg gtccaactac cgggcggtcg acgcggacaa agcgcctccg

31141 gggccgagcg ctacccgggc atgcgttggc ttggtttttg gttcagccga cggctagacg

31201 ggcgccgcca cgtggccgaa agggctgcca aagcaatggc ggtcgctgcc cacctcaaga

31261 gctttggggc agtaagatac ggaccgcctg cggctgcact tcgcaaggca gcggtggcat

31321 gcgtgggttc ctcggccacc tacgcagccg aggcatggta caacccagcg cacaagcaaa

31381 gaggactcct caaagcattg aacaaaccgc tggttttagc ggcacgggca atcctcccgg

31441 cgtataaaac cacccccctc gtccactgtt ctcagggacg caggactacc ctcggcccgc

31501 gtcgcgctgg cctacacccg cctgaaatac ggcgcccgac taaggctcgc ggacaagggg

31561 cacccccttg tcagccgcct gcgtgaaaca cctcgtgccc gcaactcggg ccattcggcc

31621 acgaccctgc aaacggctgc acaacttctc ccgcggatcc ggagactaga gttgcgcgca

31681 cctcgcaatg ccccggattc tcgcactgac cccaccggag gagtcccgaa agaggaagcg

31741 gcccgccgct ttatcgaatg gctggacatg gtatcacctg acgatatcgt ggtatatacg

31801 gatggttcgg aaaaacacga aaacaactgc gtccaaatag ggtacggatg ggccgctttt

31861 agggcgggcc tggaatttgc cgcaggctcc gcatctatta cgccggaaag ccacgttttc

31921 gacgccgagg cgattggcgc cctaaaaggg ctacaggcgg cagccaaggc ccagccacgg

31981 cgcccggatc tggatttgtg tggacagcac ctcggttatt tggggtctta gaggcgacgc

32041 gccgcgttcg tcccaatggg cctttctgga gttccatgac cttgtcgatc tactccgaaa

32101 acaaggtacc gaggttcggg tccgttggtg ccctgggcac cagggaatcc cgggaaacga

32161 ccgggctgac gagctggcca aggccggctc cgccggaccg ccggacccag acccgagggc

32221 tcagcaaacc acgtatagcg gtgccggcac ggtcctcaga gccattcttt cgaatataga

32281 gaaggactgg tggcgtaaag aactctgtga acggtccccc gcatataggg aatggaaatt

32341 ccaatacaca ccgagaaagg agcccgagga actgcgtttg ccaagacccc tactgggcca

32401 ttatttggcc atgaggaccg gccacggcga tttcaaggcc taccatgacc gtttcaacca

32461 ccaggatgca aacacctcgt gtgcctggtg ctggaagcgg acctcccctg agcacccggt

32521 gcactgccgc tattcgcggg cggtgtggag aaactggccg tggcctaata acgaccggcc

32581 ggccgggccg ccaaatcgcg cccaacgctg gaaattcttc cagacaagct tcgggcaacc

32641 gaaaagcttt gaggcgtttt cgatagccac caactacttc agcgcccgcc ccagagcggc

32701 ccgccagcgc cccgcgcgcc acgaacgcac tttacgccta gggacaccga tcgtaaacga

32761 ctcgaactct gacgaggaat agacttactg ccttttcacc cacacttcac ggcacaagcc

32821 gtcagacgaa ccctccgtgc accttaggca cggggtcgcg tcagtgaact aaccccctaa

32881 gcaggaccgg gcccgaaccc ggtcaggcac gatccgcctc tgccctcctt gttttccccc

32941 tgtgtaaata aagaagatag aacgcgcgcc gagatacccc tcgggaggtt gctaacggcc

33001 ggctaacaag ccgggccgag cccggcgtta actaatacta ctactactac(MGL, 1113-5977, str+) taa**ttagggg**

33061 **attctta**agg ggcattgaca atctttgtat ccggagaggc caaggaaaag agtatacatg

33121 atcgaggctt tcaggtctct tgttaaatcg gaactctgcc ggcaaagctt caaatgattt

33181 tcataggggc tacactaacg agcactcacc tcccactatt taacacttgc acttacgagt

33241 atccattcta tacaacttca tttatcccag acgatacgat ttgatacgca ccgcactttt

33301 ggagcgaaaa tacctcccta cggacatttg cgacaagtat ggcaccccgt ttttgatagt

| 32 | AACU02000348 |
| --- | --- |

159961 gcttctattt ctctcagaca gaccccgtga cattccaaac acattccagg ggcgacgaaa

160021 aattctatgc tcaaatagtt atgagtggtg atcactgatc tgaccattca gcgagtcatg

160081 atgagctcac tgttacctat tatgctactg gtggatacat ggggaaaggc aagacgcggt

160141 gcccatggcg cccaagaaaa gaacacgata tttcattgct ttcaaaagtg cagatttctc

160201 gcagcactat ttatgtagt**t** **tatgtaattt** **a**gtagtagta gtagtagtag tattatttaa

160261 cgccgggctc ggcccggctc attagccggc cgttagcaac ctcccgaggg gtatctcggc

160321 gcgctttcta ttttctctat ttacacagcg gaaaacaagg gcatagaagc gaatcgagcc

160381 tgaccgggtt cgtgcccggt cctgcttaca ggtttgttca ctgacgcgac cccgtgcctt

160441 caggcgcacg gagggttcgt ct(Mg-SINE, 247-447, str-)ccttgcat ttcctacagt aaactatagt cacagggttg

160501 gaccagaccc tctaacggac aggccagcta gagccctcct agtcgctgga tacccagcct

160561 gccacgtaaa tacaaggttt gcaccttaag cctaccgcat ccacaaaaag gcctcttggt

160621 agaccgacgc gcgcatccgt cctctccggc taaaatagcg tctcttccgc ctaacatgcc

160681 gacgacatca tctgccccgg tgtacgtccc tagtgcgagt agaaagcttc gaaaagccgg

160741 cgacaccccg tacatgtcca tccacagctt agtcccccgt tggttcctgg gctagcccag

160801 cgccacaatg tggacatgaa tgcaatcgag ttcttcccgt cctccagacc cggcgcagat

160861 tcccagtgcg agtgagaagc atggggcgcc tcagtcacac tattccacca cggcccaccg

160921 tccacggggt gtaccaaggg tgcgaaccaa ggccgtgccc acgtttcttg cttcccgtta

160981 cccaaagccg tctccagcaa tgggccgacc acgtcttgct gtcgggaccc tttggagact

161041 gccgcgccgg cacagtgctt ccggtccacg tacaactaac caaccccgta acggcactac

161101 ctcctcgctg caggcaatcc cagaaggtgt gagggggtga gagggtgccc tgtcgattcg

161161 aagaaccgtc gggtggttct cagcctaatt tccctttctt ttaaaccgta ttgtctagcc

161221 ttccggggaa ggacgcctgc ctttccggct ggcaaagttt tttgtgccac gctggctcgc

161281 atcgcgagat tgggccttgt cttgtgcgcc tctatgcggg tggcgcgccg aaccgccgat

161341 cggactcttc cttcaccgcg atcagtggaa aactcgtcaa aggggagatc tgtcgattat

161401 acatgggtgt tgcgaggaaa ggcgttccgt gtagtgacgg tgcttagggc gacaggttga

161461 agagtttttc gtgtgtgcgc gcctgttagg gctacaggcg gaagtcccgc gcaaacgtta

161521 acagcatggt ccgtgcgccc atactgaaaa ttttgtgtac gggagagagt tg(WEIRD, str-)**ttatgtaa**

161581 **ttta**tgtaga cattttacca aagtgtaaac aaagccgtct ttcatttgta tgccttcctc

161641 gttggataca cgaccgaaat gaggagagcc tcgacggata aattgccaag ttatgggaag

161701 atgtagaatt gaaaagcaaa aagccaaaaa ttcatcatgg cgctcatgtg caacgccttg

161761 gccaccttat ccttcccgtg ccaaatttag tcttggttag acgccttatg atttgcagcg

161821 acagggaggc tagtcgaggc cagctagacc cacgcgtaaa tagcccgctt tgacaaggtg

| 33 | AACU02000185 |
| --- | --- |

6361 tgatttcagc cgtccttaag cttatatatc aatgaaacag gtcaatactc taaatgatag

6421 tctatgcaaa tggcgggcac taggcagtcc ttttatgtag cctggccaca ctcagtatgg

6481 caccatactt tggcatattt acacccactg tctgttgcca tactgcacgg aaaagacacc

6541 tgcctccttc tttttctcgc ccgggagtcc tttccaacca acggatcgat atattatcca

6601 atctttccat cgacgtggcc cgggctgacg ctctggaaaa gccggcgttt cttcttgtac

6661 gaggcaacgt agaaggccca cacgcgttga aactggtaat tgtcggttcg gtcgagaccc

6721 tccattccac aggtggggct agtgactttg tttagccttg cacctgagcg ataggcgcat

6781 aaacaagtcg aacagatgcg tgttgctggt gagtcggtcc cgaagagata tgctgcgcag

6841 gcgtccagct cacccactct c**acttgatgg** **tgaaa**gtagt agtagtagta gtagtagtat

6901 tagttaacgc cgggctcggc ccggcttgtt agccggccgt tagcaacctc ccgaggggta

6961 tctcggcgcg cgttctatct tctttattta cacaggggga aaacaaggag ggcagaggcg

7021 gatcgtgcct gaccgggttc gggcccggtc ctgcttaggg ggttagttca ctgacgcgac

7081 cccgtgccta aggtgcacgg agggttcgtc tgacggcttg tgccgtgaag tgtgggtgaa

7141 aaggcagtaa gtctattcct cgtcagagtt cgagtcgttt acgatcggtg tccctaggcg

7201 taaagtgcgt tcgtggcgcg cggggcgctg gcgggccgct ctggggcggg cgctgaagta

7261 gttggtggct atcgaaaacg cctcaaagct tttcggttgc ccgaggcttg tctggaagaa

7321 tttgcggcgt tgggcgcggt ctggcggccc gaccggccgg tcgttgtcag gccacggcca

7381 gtttctccac accgcccgcg aaaagcggca gtggcaccgg gtgttccagg ggaggtccgc

7441 ttccagcacc aggcacacga ggtgtttgca tcctggtggt tgaaacggtc atggtaggct

7501 ttgaaatcgc cgtggccggt cctcatggcc aagtaatggc ccagtttagg ggtcttggca

7561 aacgcagttc ctcgggctcc tttctcggtg tgtattggaa tttccattcc ctatatgcgg

7621 gggaccgttc acaaagttct ttacgccacc agtccttctc tatattcgaa agaatggctc

7681 tgaggaccgt gccggcaccg ctatacgtgg tttgctgagc cctcgggtct gggtccggcg

7741 gtccggcaga gccggccttg gccagctcgt cagcccggtc gtttcccggg attccctggt

7801 gcccagggca ccaacggacc cgaacctcgg tgccttgttt tcggagtaga tcgataaggt

7861 catggaactc caaaaaggcc cattgggacg aacgcggcgc gtcgcctcta agaccccaaa

7921 taacccgagg tgctgtccac acaaatccag atccgggcgc ctggctgggc cttggctgcc

7981 gcctgtagcc cttttagggc gccaatcgcc tcggcgtcga aaacgtggct ttccggcgta

8041 ataaatgcgg agcctgcggc aaattccagg cccgccctaa aagcggccca tccgtaccct

8101 atttggacgc agttgttttc gtgtttttcc gaaccatccg tatataccac gatatcgtca

8161 ggtgatacca tgtccagcca ttcgataaag cggcgggccg cttcctcttt cgggactcct

8221 ccggtggggt cagtgcgaga atccggggca ttgcgaggtg cgcgcaactc tagtctccgg

8281 atccgcggga gaagttgtgc agccgtttgc agggtcgtgg ccgaatggcc cgagttgcgg

8341 gcacgaggtg tttcacgcag gcggttgaca agggggtgcc ccttgtccgc gagccttagt

8401 cgggcgccgt atttcaggcg ggtgtaggcc agcgcgacgc gggccgaggg tagtcctgcg

8461 tccctgagaa cagtggacga gggggtggtt ttatacgccg ggaggattgc ccgtgccgct

8521 aaaaccagcg gtttgtttaa tgctttgagg agtcctcttt gcttgtgcgc tgggttgtac

8581 tatgcctcgg ctgcgtaggt ggccgaggaa cccacgcatg ccaccgctgc cttgcgaagt

8641 gcagccgcag gcggtccgta tcttactgcc ccaaagctct tgaggtgggc agcgaccgcc

8701 attgctttgg cagccctttc ggccacgtgg cggcgcccgt ctagccgtcg gctgaaccaa

8761 aaaccaagcc aacgcatgcc cgggtagcgc tcggccccgg aggcgctttg tccgcgtcga

8821 ccgcccggta gttggaccgg ggtaaccgtt ctaccattaa tccggatttc cgggttgcga

8881 ccgtgccttt tcttagtgat atggatcacc tctgtctttt ccgctgcaaa gtggatcttc

8941 tcttccgccg caatttcgtg catatcccgg agtttatctt ccagccaacg tacgttttcc

9001 tccaacgagg gtgacgattt ccatgtaagc acgtcgtctg cgtatgccaa ccgccacttc

9061 gtaccgtttt tgacgagata cgccaaatat agcatatata ggatccgggg aaaggggaga

9121 cccttgcggg gtgccgcacc caagccgcct aaagcccgtg gtcgtaccct ccagccggac

9181 tcgggcctgg cggtcgttta aaaagttaat tacgaacctg aggagcattt tactaaaccc

9241 aaggctccgc attttccgta ttaatcgccc atggaggagg gcgtcgaagg cgccttggga

9301 cgtcgcatgc gacaagggta acttcctccc cgcgagctaa agcctgctcg atatcgtggg

9361 cgagggcaca aaccagatcc gtcgccgatc gtttgggtag ggcaccgccg tgggtgcagc

9421 taaggagccc gttgtcgtgt atggcccaag ctatccggcg tgcaacgagc ctctcgaggc

9481 cttttccgat gcaggagagg agggctatag gccgccagga tcgaacgctg ctccggtctt

9541 tcttccccgt tttcggtaac atcgcgactt cggccttctt ccaaggcgct gggaaatgtc

9601 cgagttccag gcaacgttgg tagagcctgc gcaccggctc ggccaacgaa gcccatccgg

9661 cttttagtag ccggacggtc attccgtcga tgcccgggga cgtgctcgta accccaatgc

9721 aggagcgctc cgcctcttcc gcactaacct ggggtgtccc aagggatttt agcctcgtcc

9781 ggcgaccagg cctccaaggg gtctccctgc aggtcatcct ccgccgaaaa tcggccaagc

9841 acctcccgtt gcagtgcttc cgcttttgct aaaggctcgc tcacttgctc gccgttaata

9901 accagcggcg gggaccggag gcgtggtccg gctcccagcc agcccaccat gttccacaaa

9961 tccttatcgt cgcgcagttg gtcgatccgg tgcctccagt actcccgctt cgcggcccgc

10021 acccctttcg tataggcttt ttcctcggca gaggcgtctg ccctatttac agcgctccgt

10081 ttaacccgct ggaattcgga ccagagccgc tggcagtttt cggtccacca gggagccgag

10141 ctgtcccttt ttcccgccgg cgtacccacg acccacgtca cttgctccca taaagttgta

10201 aatttagcta cccacgcgtc caatgcgtag ccatcggccg cggatcccgg gtccggcatg

10261 tcttgcatgc caaaagcaag tagctccgcg aactttggta accggtcgtc cctaaccgaa

10321 acgttgatcg cctccgggcg gggggcgccg cgcgtccgca gcgtggtata aagggattcg

10381 tggtcggagc ctgtgtaaag actgctgtca accacagtta ttgtgctggg gaggttggaa

10441 aagaccatat ccagtaaacc cccgtcgcgg tgggtgggca cgccgatatt tcctgtaaag

10501 ctcatcccct gggcttgcgc ccatgccgtc agttggtccc ctccgcgtgc gtttgcgcgg

10561 cccggctggt atgcagcagc cgctacgtta aaatcgccgc ctagcaccgt gggtccattt

10621 ggcacggtat tgcataccat ttccagcgcg gcttcagtgc ccggagccct atatacgtta

10681 acaaacaata ttccgttaat ttcgagccag agtatatccc gcgtattttg gccctgcggt

10741 agccgtcgtt gtgcggccct tagggctgcc cccttcttaa cgtaggtgag cacccggggc

10801 cggagcccag tcatggtttt gtaagtgttg gcgtgccatt cgtccactgg cgcaaaaaca

10861 ttatagcccg ggtgcgtttg tgtagtcgta tttagcccgc accatggctc ttgaacgtta

10921 actcaggtcc acctttctct ggtggcacaa ctccaatagg ctcagatgca cacccatcct

10981 tttacctacg ttggcccaaa ctacgtcgag gcattcccgc tgcatattgc cgagcgagta

11041 tttcgttata ttaatcgacg ggttgcttcc aagtctatcc catcagcggg gcaaggttcg

11101 gcagccgctg cgtcctcctc catttcggct gcccaaacga tttcgtcgtg cacgcgttcc

11161 tgttcaacct gcaggaagtt acggatgtcc gcccctgccg cctcgcatgg ccgcgctctt

11221 ttgtttgaaa tgctcgattg ggagctcgag gtttgcgaat(MGL, 1608-5977, str-) gtagtagtag tagtagtagt

11281 agtagtatta tttaacgccg ggctcggccc ggctcattag ccggccgtta gcaacctccc

11341 gaggggtatc tcggcgcgct ttctattttc tctatttaca cagcggaaaa caagggcata

11401 gaagcgaatc gagcctgacc gggttcgtgc ccggtcctgc ttacaggttt gttcactgac

11461 gcgaccccgt gccttcaggc gcacggagga ttcgtctgac ggcagttgac ggctcttcat

11521 cgagaggggc ctgtgtccaa acagcggagc tgtcggtcgt ttgtagccat ggagacgaag

11581 ttcccaacaa gtgtgggctc gacggcaca(Mg-SINE, 135-472, str-)c cttgcatttc ctacagtaaa ctatagtcat

11641 cagggttgga ccagaccctc taacggacag gccagctaga gccctcctag tcgctggata

11701 cccagcctgc cacgtaaata caaggtttgc accttaagcc taccgcatcc acaaaaaggc

11761 ctcttggtag accgacgcgc gcatccgtcc tctccggcta aaatagcgtc tcttccgcct

11821 aacatgccga cgacatcatc tgccccggtg tacgtcccta gtgcgagtag aaagcttcga

11881 aaagccggcg acaccccgta catgtccatc cacagcttag tcccccgttg gttcctgggc

11941 tagcccagcg ccacaatgtg gacatgaatg caatcgagtt cttcccgtcc tccagacccg

12001 gcgcagattc ccagtgcgag tgagaagcat ggggcgcctc agtcacacta ttccaccacg

12061 gcccaccgtc cacggggtgt accaagggtg cgaaccaagg ccgtgcccac gtttcttgct

12121 tcccgttacc caaagccgtc tccagcaatg ggccgaccac gtcttgctgt cgggaccctt

12181 tggagactgc cgcgccggca cagtgcttcc ggtccacgta caactaacca acccccgtaa

12241 cggcactacc tcctcgctgc aggcaatccc agaaggtgtg aggggggtga gagggtgccc

12301 tgtcgattcg aagaaccgtc gggtggttct cagcctaatt tccctttctt ttaaaccgta

12361 ttgtctagcc ttccggggaa ggacgcctgc ctttccggct ggcaaagttt tttgtgccac

12421 gctggctcgc atcgcgagat tgggccttgt cttgtgcgcc tctatgcggg tggcgcgccg

12481 agccgccgat cggactcttc cttcaccgcg atcagtggaa aactcgtcaa aggggagatc

12541 tgtcgattat acatgggtgt tgcgaggaaa ggcgttccgt gtagtgacgg tgcttagggc

12601 gacaggttga agagtttttc gtgtgtgcgc gcctgttagg gctacaggcg gaagtcccgc

12661 gcaaacgtta acagcatggt ccgtgcgccc atactgaaaa ttttgtgtac gggagagagt

12721 tg(WEIRD, 1113bp)**acttgatg** **gtgaaa**cccc gtggtaccta ttaaatattg cttcatatcc gcagctcgca

12781 taattgcgcg tcttgtgtca tctcctggca tcgaatgaat caaagtagcc tagctatgag

12841 gagtcaaatt ggttgaaagg gttccgtagc agacactcat ggttgcgggt cggcatcatg

12901 tgaattggat atcataggca acttggcaga ttaacccagt cgtttcgaca tttcttcatg

12961 ggttaggtat atgtgttgga cacggcagcc aagcaggacc ccgtcccacc gtagcagtcg

13021 ccctggagtt cctggcccgc cctggcttcc tcgtcccttc ctaccatttg cttatgctag

13081 cactccccta tccgccggat ttggcgcatt ttcttctacc tgtcttagct gtgtacctac

13141 tgacatatct gtcgcggtct gcaggcggct tcctcggagg aacctgagcc tcttgatgga

13201 agtattccga ccgttggagt ccaatggaag ctgcctgtgc tgcctggccc tgctgaaaag
