# Supplementary material for: Tripartite chimeric pseudogene from the genome of rice blast fungus Magnaporthe grisea suggests double template jumps during long interspersed nuclear element (LINE) reverse transcription
Source: BMC Genomics. 2007 Oct 8;8:360. doi: 10.1186/1471-2164-8-360 (PMC2104539; doi:10.1186/1471-2164-8-360)
Supplement: Additional file 2 — Mammalian triple elements. The data provided represent the detailed sequence information on the triple chimeric retrotranscripts identified in the mammalian (mouse and rat) genomes. [file 1471-2164-8-360-S2.doc]

**Mammalian triple chimeric retrotranscripts**

# Mouse triple elements

>AC084162

75001 ccaacctggg gtacaggaga atctgtctca aaagtgagag actgggaatt caggccagaa

75061 gataagaaaa aacacgggta gaaagcagcg gcattttttt tttttaaagc atgggcccaa

75121 acaaggcagt cagatgacca aacaggtcaa gggctactgg gagacaggta caggctgctg

75181 tctgggttat gggccaaatg atagctcacg gtcacaagct aactaacacg cggtgcactt

75241 cggagcctgc ccgagggagg cctccttacc aggctttgat ggggcaggtt tggtcaaggc

75301 tttcagaact gggatggtag gttccagcca aagccctcac cctggaacac gaccgggatg

75361 aggcggtccc ccctccagcc ctcttctgcg gcacccagcc ccacccaccc ccctgctggg

75421 gccctcaggg atctcgaagt cttccttggc ccaatagggc agatgccctg ggctctggtg

75481 gggggaggag tctggggtct gaaccgggtt cccaccttct ctcagtttcc ccttccctct

75541 ttctccgcag tgcgggaaaa catttggact gtaataaaaa acactaggtg cccgggtgac

75601 aggctgcgac tgagtctctg taactgcgag catcagggag cagggaggac tggggtctct

75661 ccccataagg actgagtggg gctctcattc ctcccgaggg gcccagtgag gagaggtggg

75721 tggcctcaag gaaccaggtc ccatcacccg accacttgcc tctgaggttc aagtggaaag

75781 aaaaagggaa tcaagaaaat gtccttccac cctggagggt ttgtgggcta tttgttaaaa

75841 ttattgtgtg tgacacacac ctttaacctc agcactctgg agatagaggc aggcagatcg

75901 ctgtgagttc aaggctagcc tggacctcag agcagattcc agcagagcca gaactacaca

75961 gaggaactct gtctcacacc ccaccccaat tattgtgtgt acatatgcaa tatgtttgag

76021 gacgcatgtg ccactgcacg tgtgtggaga tcagaggaca actgtgtggg gtcagctctc

76081 ttctgccaca tttatatgag agccaactct ggccaccagg cttgtgaggc aagtaccttt

76141 accagccgca tcattctgtg gctagtgggg ggacagcggt gtgtgtgtgt gtgtgtgtgt

76201 gtgtgtgtgt gtgtgtgtat ccggtatgta gtatgtatgt acactgtgtg gccatgtttg

76261 ttttgtttac t **tgagacagg gtctccc**aa g gctagagaga tggctcagtg gttaagagca

76321 ctgactgccc ttccagaggt cctgagttca attcccagca actacatggt ggctcacaac

76381 catctgtaat gagatttgat gccctcttct ggtgtgtctg aagacagcaa cagtgaactc

76441 ataaataaaa taaataaata aatctttaaa aaaaaaaaga B2_Mm2 3 194 (1) gtgctcgctt tggcagcaca

76501 tatactaaaa ttggaacgat acagagaaga ttagcatggc ccctgcacaa ggatgacacg

76561 caaattcatg aagcgttcca tattttt U6 1 107 (0) gcc tcagtacagg ggaacgccag ggccaagaag

76621 tgggaggggg gtaagggagt ggggggggat atgggggact tttggggtag cactggaaat

76681 gtaaatgaag aaaataccta attaaaataa aaaaa L1Md_F2 6444 6580 (2) **tgaga cagggtctct** **c**tgtgtaacc

76741 ctgactagcc tggtagacca ggctagacta gaactctcga taataatcct cctgcctcag

76801 tctcctggtt actgggattt cagtcacagg ccaccatgcc ctcccgttcc tcgtcctcct

76861 gtctgccccc ctttttgtct cccctttgtc ttctgtgtgt gtgtggtgtg tgtatgtact

76921 ttatgtgtgt atgtactatg tgtgtgtggt tgtgtagtgt gtgtatggtg tgtgtggtgt

76981 gtgttgagtg tatactgtgt gtggtgtgtg tgtactgttt gtgtgtgtgg tgcatgtact

77041 ttgtgtgtag tatagtgtat ggtaggggga tatgtgtgaa ggggtagtgt gtgtgtgatg

77101 tgtgtatagg acaaaggcca acattaaatg tctttcctct atttaagctt ttccacccta

77161 atctgaggca gggtctccca gactctggag ctcaccagtt ggctagactg actggccctg

77221 gacttgtctc tgcttcacac acacacatac atacacacac acacacacac acacacacac

77281 acacacagag agagagagag agcatggcag catccagaca ggcatggtgc aggagatgct

77341 gagagtgaag gctgctagca gaatactggc ttccaggcag ctagggtgag ggccttattt

77401 cccacaccca cagtgacaca cctactccac aaggctacgc ctactccaac agggccacct

77461 cttccaatcg tgccacttcc tgggccgagg atttacaaac catccatcac actggtctat

77521 atagggagtt tcaggacagc cagggctaca cagagaggct ctgtcccaaa acaaaaagaa

77581 tcacacacac acacacacac acacacacac acacacgagt gctttcttat tattttcctt

77641 ttctctcttt ctttctttca ttcattcatt tttctttttt tttaaaaaag agtaaaccat

77701 gctcgcctta accatcagtt tgttttttgt tgtttttttt aagatttatt tatttattat

77761 atgtaagtac actgtagctg tcttcagaca ctccagaaga gggcatcaga tcttgttaca

77821 gatggttgtg agccaccatg tggttgctgg gatttgaact ccaaaccttc ggaagagcag

77881 tcgggtgctc ttacccactg agccatctca gcagccccat tcatttttct tattccatgc

77941 cctgtgtggg ggcgctgggt tgcatgttta tatgtggacc acatactcta tgcccgggga

78001 gatcagatct cctgaaactg aagttatagt ggttgtgagc caccatatgg gtgctgggaa

78061 ttaaacccca gtggtctgta aaagcaataa gtgctgcgat tcaccactga gccatctctc

78121 caccccctaa tttctgtttt taaattttac gtgtatgtgg atggtgtggg acataacgca

78181 atgcgcacat ggacgccaga gggcgaactt tgtagagtta gttctctcct tcttcctcta

78241 catgggtttc cagcagcaaa ctcaagttac caagcctatt ttggcaaatg cccccagcta

78301 ctgagccatc tcagcctacc actattatca ttgtcgttgt cgttgttgtt gttgttgttg

78361 ttgaaagcac agagaccttc atcctgtcag attcccaaac caaacagcca cagggaaaag

78421 gaaatatctc cccaagacgg tgcactgtta tttccaaaat gcaccctcgg ctattcattt

78481 atttgttgtt tccctaaatt cccccgccac actcccatag ggtttctttg tgtagccctg

78541 gctattctgg aactcacaag agttctggcc aagaactcac agagatctgc ctgcctttac

>AC090495

63001 aaatacagtc attttccagc ttcctgtact tgtgggttct gtttctacag agtcaggcaa

63061 gttcaaactt tttctaatta taaaaagccc caagagtgca aacctgaact gttgtacatg

63121 aaagaatgtc taatagtggg atggatatgg agtgatgtgc tggctatctt tccatagctt

63181 ccctcccatg gggtcataag ccggtttccc tctggcatcc gctgcttaag acagcgctgt

63241 ggtggtcttg tctgtcttaa aagtgtagtt ttgttgttgt tgttgttgtt tggagggaaa

63301 ttccattgtt tctaaaaaat gttagacaac acagtcacat tagatgctac cagtaatcta

63361 gagatggcca agtgagtggg acagtattta taggttatat gcacatgcct tgccattttt

63421 ctaaaaaaaa ttgtttattt atgtgtatga atgctctatt tgcatgcgtg cctacataac

63481 agaagagggt ctcagatccc agtatagatg gttgtaagac accatgtagt tgctgggaat

63541 tgaactcagg acctctgaaa gaacagctag cactcaacct ctgagccatc tctccagctc

63601 tctatacctt gtcattttga gaggtacctg aacatcctca gatcttgtct ccatggggct

63661 ttctaatgta gcatccatga cggactggac atacacttag tggtgtttgc tcaacacact

63721 ctcactgagc tcctatctgc caaccttgtg tggccatact gatgatgaga gcgagagaga

63781 gcgagagaga gagagagaga gagagagaga gagagagcat tgtccttcat caaacagcaa

63841 gagggctgtt ccataccatg aaaagagcag ttatcagtct cagctgcagg ctggacacac

63901 ccatttgtca ctacctttat tcctttcaca aacccttaag gtaaatccca tatttccatt

63961 ttacagagga agaaagtgag gctcatagag ggtgcacata gccaagccta ctaagtgcct

64021 agtcaggatg g**gctcccaga** **actttgt**ttt tttttttttc catttttatt aggtatttag

64081 ctcatttaca tttccaatgc tataccaaaa gtcccccata tccacccacc cccactcccc

64141 tgcccaccca ctcccccttt ttggccctg L1Md_A (0) 6582 6453 g tgttccccta aaaaatatgg aacgcttcac

64201 gagtttgcgt gtcatccttg cgcaggggcc atgctaatct tctctgtatc gttccaattt

64261 tagtatatgt gttgccgaag cgagcac U6 (0) 107 1 t aa gatttattta tttattatat gtgtgtacac

64321 tgtagctgtc ttcagatact ccagaagagg gcatcagatt tttgttacga tggttgtgag

64381 ccaccatgtg gttgctggga tttgaactca ggacctttgg aagagcagtc ggtgctctta

64441 accgctgagc catctcacca gccc B2_Mm1a (15) 178 2 **tctccc** **agaactttgt** **aa**ctgaaagg ccttcaaacc

64501 agcactaaac aagcgttgaa tgtagctgga agctttctcc taccgttgaa ctggaaagtt

64561 cctctctggc tgaataaaaa gtccactgaa atctttcagg tagcctgtga ggccaccagc

64621 cacgacttcc agctggtcta acttctgcag tgtctgaatg gaggatccac cactgttccc

64681 aggacccgaa aaccataata ggacaagacg ctcgggccca ttggacccta gagatctctg

64741 ttgtagagtg aggtcctaga cgcgatctcg taactcacct cctggctcta agatacagtg

64801 agtcatgctt tgatgtgatc actgcttgac aataccgttt gggtctgtgt gtctgagtgc

64861 gcgtttgggc aggtacaggc cagaggtcag ctctgggcac tgtcctcaat ttctctccac

64921 cttattgttt cagactcggc ttctcactca acctgacgct ctctgcgcct gttaggttag

64981 ctggatgaca gtcccaggga tccccctgcc tctacctccc cagtgctggg attatagatg

65041 cacactgctc atgacccttt tacatgttac aaaatcaggg gcttgtgctt gtctgccttg

65101 cagtttactg ctgagccatc cccagggttt gttttttgtt gagatctctg tcgacttact

65161 aaatgttccc ctgaatctgg gacttacagg agagataaaa ggataaagct cctggaaaca

65221 cacatctggc aacagattta aagatgatta aatctgtgat tacttctgat ccatataata

65281 gttcagagat gtgagtgtgg taaggcccaa atgcagagaa aaaaacaaaa aaaccaaaga

***Human triple elements***

>AC005037

182101 ctctcgagta gctgggatta caggcgcccg ccaacatgcc tggctaattt ttgtattttt

182161 ggtagagacg gggtttcgcc atgttggcca ggctggtctc gatctcttga cctcatgatt

182221 cgcctgcctt ggtctcccaa agtgctggga ttacaggcgt gagccaccgt gcccggccag

182281 gaatagattt ttcagaggga aattctggct ggctggtgct ggtatttgag tgacaatgag

182341 attgactgtg gtagtgtgga caaactgcaa actggaacca agtgccactg ccagggtgaa

182401 gaaccattgc tgtagtgaca ctgacaggaa aaagaagcaa aaaggagcaa gcccttcttc

182461 ctctttcatc attctagtct tcctctagaa tcacctgtag gaagtctaat tgggaaccag

182521 atggcataag aaatccagtc tgcagagttc cagccccagc atcgcagaag acaaaatggt

182581 gtgtttgaag ctgagagata atagcttaat aatcgatggc ttaataatac ttgcttttat

182641 tccctctatg atgatgtatt caggttttct ccttcttaag tctattttga ctgtttgttt

182701 tgctattttc ctccatattt ctaaatgacc attcaggatt gaacccagca atcagtaaaa

182761 ataaacaggt ttttttgtta cctgaaaaaa gtctagggat gtaggttgtc cagggatgga

182821 atgccgaact ttaatgtcag gaactcaggc tctattttgt tgcactgcaa ttctcagcaa

182881 atgattcttc atcatgtcta cttcccagct agcaggaaga gggaagggaa aagacatgac

182941 ctctcttttt ttttttttct gagacagagt cttgctctgt ctccagcctg gagcgcagtg

183001 gcatgatctc agctcactgc aacctccaac tccctggttc aagtgattct cctgtgtcag

183061 cctctcaagt agctgggatt acaggcacgt gccaccatgc tcagctaatt tttgtatttt

183121 tagtagagac gggttttcac catgttggcc aggatggtca ctatctcctg acctcgtgat

183181 ccaccagcct tggcctccca aagtgctgag attacaggcg tgagccacca cgcccagtct

183241 acgtgacctc tccttttaag ggcactagac gtggcccatc ccaatctttt tttttttttt

183301 tttttgagac agagtcttgc tctgtcaccc aggctggagt gcaatggcac gatcttggct

183361 cactgcaacc tctgcctccc aggttcaagc gattctcctg cctcagcctc ccaagtagct

183421 gggattacag gtgccagcca ccatgcccgg ctaatttttg tacttttagt ttcgccatgt

183481 tggccaggct ggtctcaaac ttctgacctt aggtgatcca cctgcctcag cctcccaaag

183541 tgctgggcat gggccaccat gcccagcctt ttttttaaga gacaaggtct ttctctgtca

183601 cggagttcgg tagtgtgatc atagctcact gcagccttga attcctgggc tcaagcaatc

183661 ctccctcctc agcctcccaa gtagctcact tgattacttt ttttgtagag atgggggtct

183721 ccgttgccca ggctggtctt gaactcctgg cttcaggcaa ttcttgtctt ggcctccgaa

183781 agtgctgggg ttgcacgtgt gagacactgt gcctggcttt attttaattt ttattattaa

183841 ttaatttatt ttttgagacg gagttttgct tttgttgccc aggctggagt gcaatggcgc

183901 gatctcggct caccgcaacc tccgcctccc gggttcaagc gattctcctg cctcagcctc

183961 ctgagtagct gagattacag gcgtgtgcca ccacgcccgg ctaattttgt atttttagta

184021 gagacggggt ttctccgtat tagtcaggct ggtctcgaac tcccgacctc aggtgatccg

184081 cctgcctcgg cctcccaaag tgctgggatt acaggtgtga gccatcgtgc ccggcca **tta**

184141 **ttttt**atatt ttttaaatta aa ttttttta ttatacttta agttctaggg tttgcacaac

184201 gtgcgggttt gttacatcat atgtatacat gtgccatgtt ggtgtgctgc acccattaac

184261 ttgccattta cattgggtat atctcctaat gctttccctc cccactcccc ccaacccacg

184321 acaggccccg gtgtgtgata ttccccttcc tgtgtccaag tgttctcat L1PA5 (6) 6148 5941 a aaaatatgga

184381 acgcttcacg aatttgcgtg tcatccttgc acaggggcca tgataatctt ctctgtatcg

184441 ttccaatttt tagtatatgt gctgccgaag tgagcac U6 (0) 107 1 ta t tttttttttt tttttgagac

184501 acagtctcgc tgtcacccag gctggagtgc actggcgcga tctcggctca ctgcaagctc

184561 cgcctcccgg gttcacgcca ttctcctgcc tcaccctccc gagtagttgg gactacaggc

184621 gcccgccacc acacctggct aatttttttt tttttttgta tttttagtgg agatggggtt

184681 tcaccatgtt agccaggatg gtctcgatct cctgacctag tgatctgccc gcctctgcct

184741 cccaaagtgt tgggattaca ggcgtgagcc actgcgcctg gct AluY (14) 297 1 **tttattt tt**aa tacggc

184801 acttttttac tctgtctggt atcccccagt ccattatcac cctctcctcc cctttttcca

184861 acttttgcag atggtgaaac ttctgttttc tgctgaattt gtgcaggggc actcacctgc

184921 cagcataggg ggaagggatg gcctaagaca ttcaataaac ttgttttcag ctccaccata

184981 ctcacaccca ctctcagaag aaattagcat ccctgactcc caagaccttg cggagtttgg

185041 cagttccaat caacttcctg ggttgttgtt agcaacctgc gacaccacaa gggcttagca

185101 gaacagagaa agctaaaacc aagctggttg ttggtcatat ctgcttgcct tcttccaaca

185161 gttggatatt tttcatttgc cattaactct cctctcttgt tcttagaagt tgataatatt

185221 tatactgtta ctacaaagga ttttgtgaga ttcactgaat acttgaccag tcacacaaag

185281 catgattatc attcactact gggctataag gatttcagct actatcttgc ttctcctttt

185341 ttatagttgc taaagcaggc atcataatga tggctgctgg cacttaaatt accttccacc

185401 agttcctatc aaacataaaa acctgtattt atggctgggc gcggtggctc acgcctgtaa

185461 tcccagccct ttgggaggcc gaggcgggtg gatcacctga ggtcgggagt tcgagactag

185521 cctgaccaac atggagaaac cccatctcta ctaaaaatac aaacaaaatt agccgagtgt

185581 ggtggcacat gcctgtaatc ccagctactt taagtagaac ttctcaactg ccccatgtag

185641 ttgagactgt cattttatca ctgggtaaaa tgataccatt tttttccccc agagatgggg

185701 tctcactatg ttgcccaggc tggagggcag tggctattct cacatatgat catagctcac

185761 tatatcctcg aactcctgga ctcaagcaat tctttatctc agcctcccca gtagctgaga

185821 ttacaggtgt gtgccactgt gcccagcaat gatatcattt aatcaaaatt agattgccat

185881 tctcatttta caggttcagg aaacaagatt cagaggctaa attatttgcc cagtcacaca

185941 gctaacagat ggagacagat ctttctccat tagactgtag gtatgcactg ttttgttcag

186001 atattaggca tttgcaaacc atattttatc agcaagtgca atttgtcttt cacggtatcc

186061 taataattta cagattatat tcttgagact ggactctcca atttgccagt ccctcccaca

186121 tcctttcgtt ttgcattcag cctactcttc ctttcctatc tcctcctatg ggcttgaatc

186181 ttggggtcca gtgctgttca ttaccatagt cttaaagctc agcagagggc ctgacagtgt

186241 atgtttcaca tttgctgact gccagcttct catcctgtct tggtcacatc aagcaatact

186301 aaccacaggg cacaaatagc tttcatctaa tcttgtctaa gaaaagttgc ttcctaatac

186361 ttggaattca cataacagtt ttcattctac aagcaaacat ccctcctagt tgtcttttta

186421 ataacagtat taagcgtgac acctacagtc ccagccactg aggtgcgaag attgcttgag

186481 cccaggagtt tgaggctgca ctcagctatg atcatgtcag tctgggtgac aggacaagac

>AL133286

aactcacaca gtgagcctct gatgccaagt ctcaacatca

80041 ctttgattat atccttgtca gaaagattta accagaatct agtcaaaagg aaacagacaa

80101 agccatattg tagagccttc cacaagataa ttggcttgga ctaaaaaatg tccatgtcac

80161 aaacaacaac aaaatgtgac agggaatatt ctgagcagtg atgaacaata gaaatgtaat

80221 gcaggcaaaa gtggaatttt aatattttta aagccacatg aaaaagtgac aagacaggtg

80281 aaactaattt ttaataatgt attttgaccc aacgtaatca atgtattctc atttcaacat

80341 gcaatcaaaa taaaatttga gtatagaata tatgaaattg aatattgaat ttaaaatttg

80401 aatatttaaa atttgaatac tgagtataaa atttgaatat ctgtgtatta gataatatta

80461 ctgtattgat gttaaagttg ttggtaatta ttatgaactg aacgtttgcg ttctctcaca

80521 atgcatatgt tgtgggagtg tagtgtagtg tattggtatt tggagatgag acctttggga

80581 gggaaatata tttagatgag gtcataaggg gcaggactct tgtgatggga ttagtgtccc

80641 cataaacaga ggaagagatg tcagagcttt ctctctgtct ccaccacgtg aagacatagt

80701 g**agaagacag** **ct** gactgggt gcggtggctc ttgcctgtaa tcccagcact ttgggaggct

80761 gaggcaggtg gatcacttga ggtcatgagt ttgagaccag cctagctaac atgctgaaac

80821 cccatctgta ctaaatacac aaaaattagc cgggtgtggt ggcgcacatc tgtaatccca

80881 gctactcagg aggctgaggc aggaggatcg cttgaaccca ggaggtggaa gctgcagtga

80941 gccaaaattg cgccacggca cttgagcctg ggttacggag caagactcca tctcaaaaac

81001 aaacaaacaa acaaacaaac aaacaaacaa a AluSx 1 312 (0) aaat gtgct tgctttggca gcacatatac

81061 taaaattgga aggatacaga gaagattagc acggtccttg tgcaaggatg acacgcaaat

81121 tcctgaagcg ttccatattt tt U6 1 107 (0) aaattgtt ctactataaa gacacatgca cacatatgtt

81181 tattgcagca ctgttcacaa tagcaaagac ttgaaaccaa cccaaatgcc catcaatgat

81241 agactaaata aagacaatgt gacacatata caccattgaa tactatgcag ccataaaaaa

81301 gaatgagttc atggcctttg cagggacatg gatgaagctg gaaaccatcc attctcagca

81361 aactaacata agaacagaaa accaaacacc acatgttctc actcataagt gagagctgaa

81421 caatgagaac acatggacat agggagggga acatcacaca ccggggcctg tcgggggtgc

81481 tagtggaggg atagcatcag gagaaatacc taatgtagat gacaggttga tgggtgcagc

81541 aaaccaccat ggcatgtgta tacctatgta acaaacccgc acattctgca catgtactcc

81601 agaacttaaa gtataataat L1PA7 5662 6144 (10) **agaa**gaagaa gaggaagaag aagaggaaga agaagagaga

81661 agaagaagaa gaagagagaa gaagaagaag aagaagaaga agaagaagaa gaagaagaag

81721 aagaagaaga agaaga c**gac** gacgacgacg acgacgacga cgacgacgac gacgacgaag

81781 acgacgacga cgacaacaac **agcc**atttgc aatctgtaag ccaggaaggg ggccctcacc

81841 aggaactgaa tcagctgaca ccttgatctt attacacctt caacctctgg aattttgaga

81901 aataagtgtg tgctgttgag gtcacccagt ctatggcatt ttggtatagc agcccaagcc

81961 tactaatatg gtaataatag tactttgttc tataggagaa tgctgttatt ctcaagagac

82021 acatgctgaa gtacttaggg tcgactcatg atatctgcaa cttactttca aatgcttaaa

82081 caaattatac atagatgata ggtacctaaa tagagataga gatacagcaa gcaaagaagc

82141 aactattaat aattggtgaa tctaggaaaa ggatattcgg gtgtttacta gtccaacttt

82201 tttcagctat gaaattttta ttttatgttt tatttattta tttatttttt gagacagagt

82261 ctcactctgt tgcccaggct ggagtgcatg atctcggctc actgcaacct ccgcctcctg

82321 ggttcaggcg attctcctgc ctcagcctcc cgagtagctg ggactacagg cacacaccac

82381 cacgcccagc taatttttgt atttttagta gagacggggt ttcaccatgt tggccaggaa

82441 ggtctcgatc tcttgacctc gtgatctgcc tgcctcggcc tcccaaagtg ctgggattac

82501 aggcatgagc caccgcgcct ggccaaaatt ttctaaataa gaagttggaa agagtaaata

82561 gagtaaaatg atctgagaga aaaagaatcc aaataataac atttatacta ttttatagtt

82621 tacagtgtat tttcacgtat attaatgtga tcctttcaaa tatccacgga ggtagacata

82681 tcgtgtgttt tatccttatt ttgtagtctt caaaaacatt tcctcagtgc ctaaccatgt

82741 gccaggcatg gtacatacat catctctttt actttctaca agaactccag aaggcagata

82801 tcggctctcc catccacgga ggctctggtg ggattccgaa gctagcaatc tgcagagctg

82861 gcatctgaat gcagctttgt ccgactctga ggcctgagct catccctctg ttggcatcct

82921 gtttgtgatt cctgttttac agatgcagga atggaaactc agagaagtta attaacctgt

82981 gcagtgtcac acagctactc ggggcaaagc caaaattcaa gccagaactt cagtctccta

83041 ttccaaagct cggacctcct tccaaagcgc ctgtaatgag cactctggaa ataggttcag

83101 gatctaagct agtggctcat gtgagaagaa gtctcagcac acaagggccc tcagcacagg

83161 aaattactca agttattgga gtggagccag ggaaaggccc cgaagctcag agcaggcctg

83221 actcgggaga cagtttgtga aaataaaagc aagcccacat tgactcattc atttgatgct

83281 ctgattaaga agcatgagtt gccacaccat gtgatggaga gctataaact ccagccctgg

83341 agacataatc tggcacagat tcccttggat gggcaaacgg tattcaggca ggactaccat

83401 ttccgggagg gggagaccgg gggaagcagg tcaggacttg aggctcataa ccatgctgca

83461 ctgatttgtt taaaagtaaa tattatttgg ttttcattta attttaaaca aaatttaaat
