# Supplementary material for: Tripartite chimeric pseudogene from the genome of rice blast fungus Magnaporthe grisea suggests double template jumps during long interspersed nuclear element (LINE) reverse transcription
Source: BMC Genomics. 2007 Oct 8;8:360. doi: 10.1186/1471-2164-8-360 (PMC2104539; doi:10.1186/1471-2164-8-360)

Putative RNA secondary structure elements, predicted upstream template switching sites

# Chimera 34

**
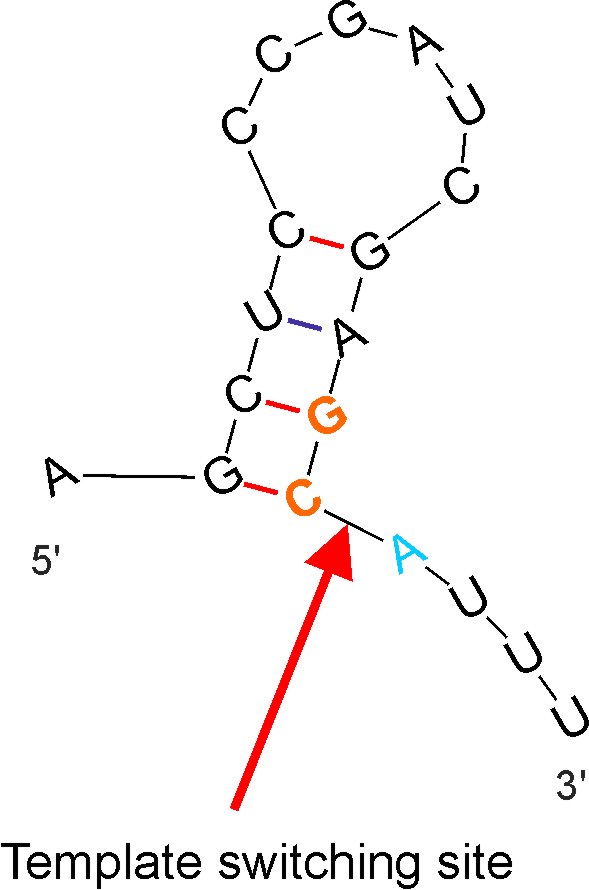
**

## Chimera 23

#
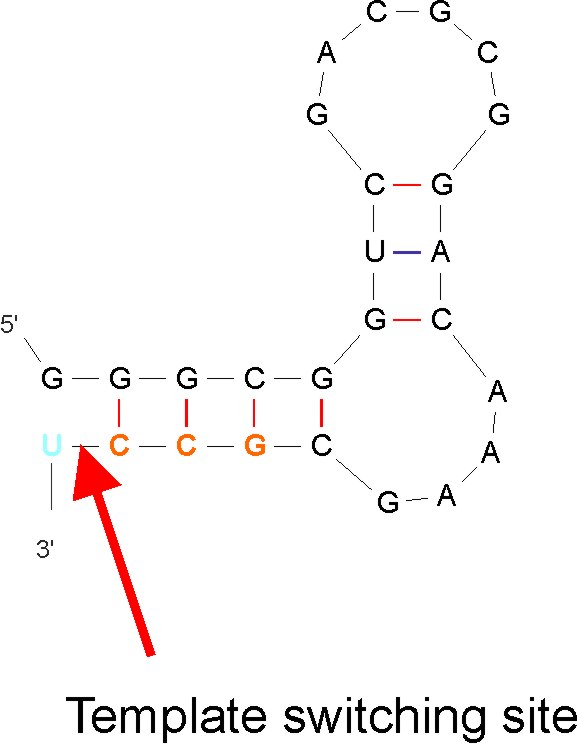


# Chimera 24

**
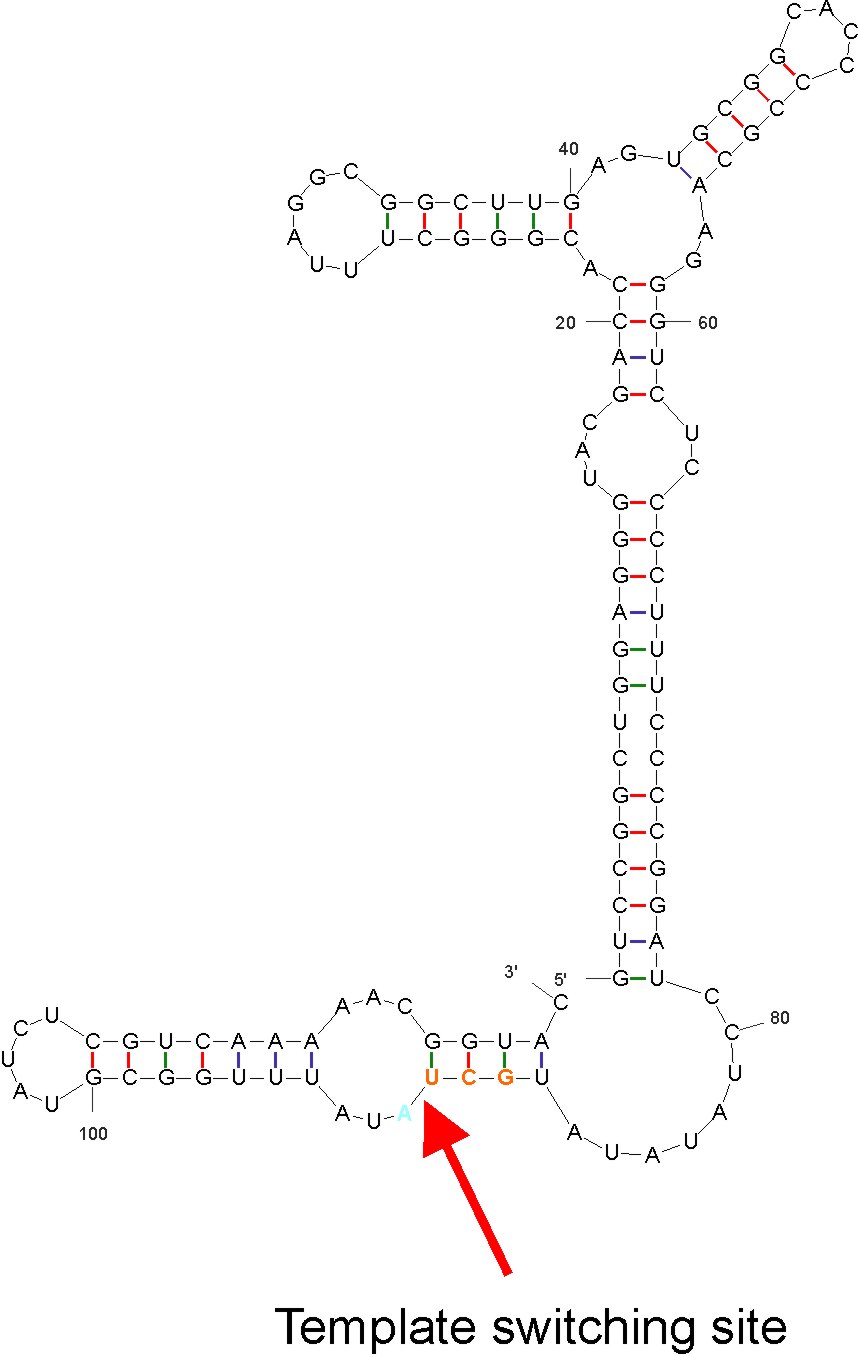
**

## Chimera 16


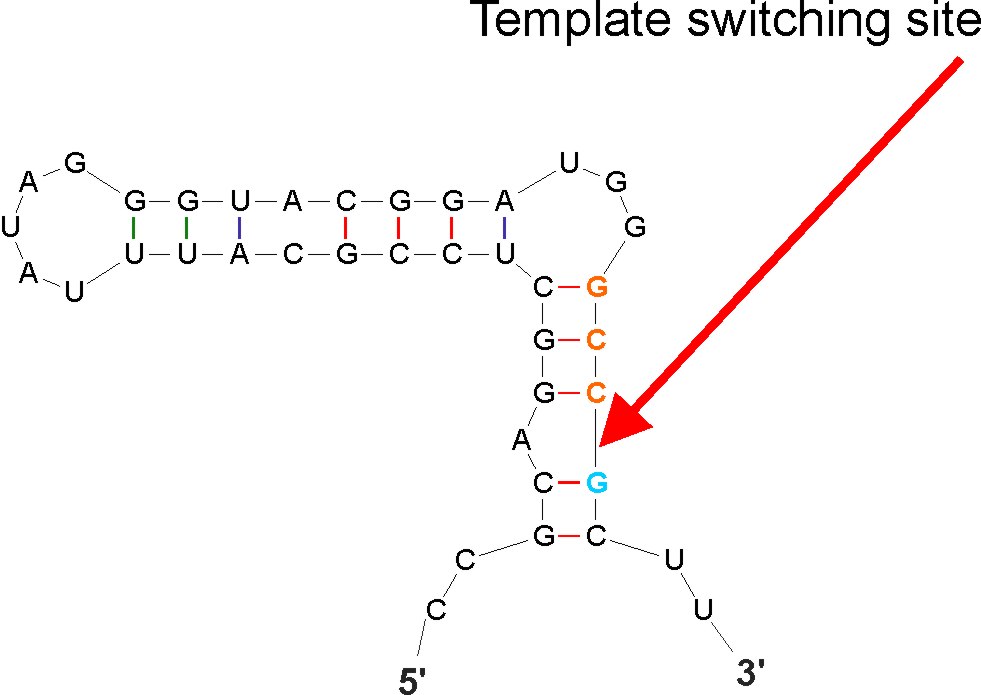


Chimeras 4 & 15


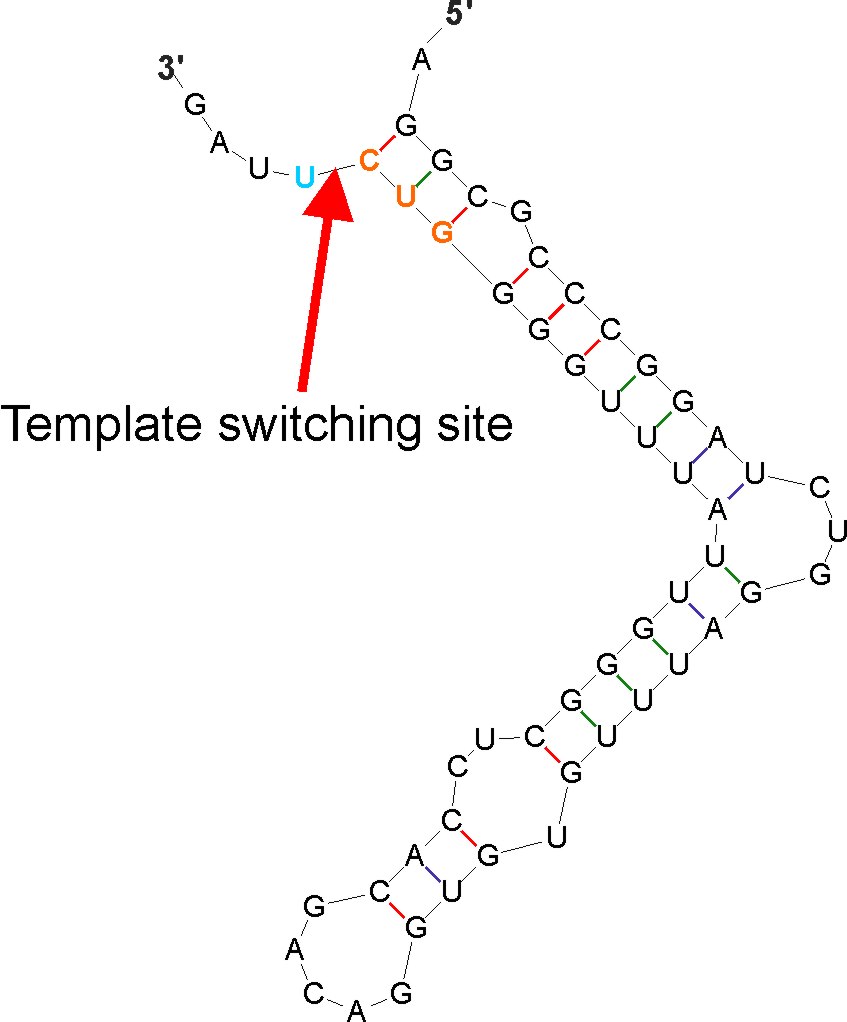


Chimera 14


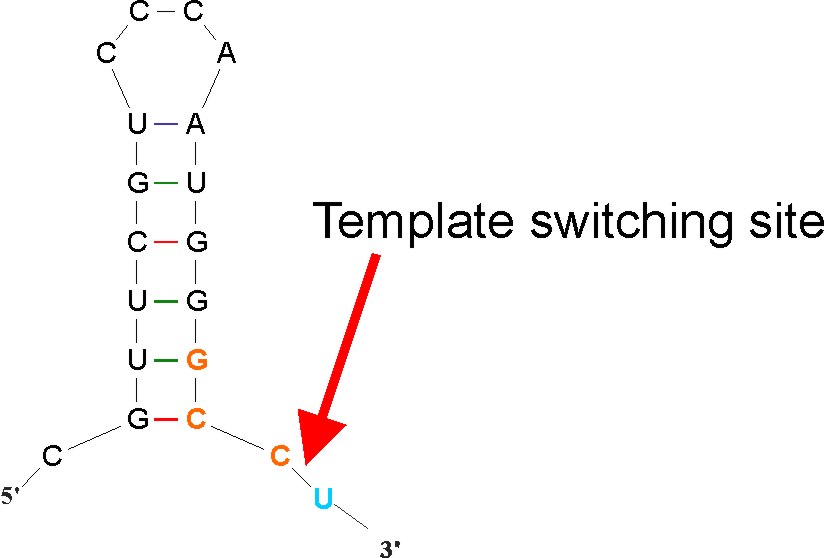


Chimeras 13 & 12


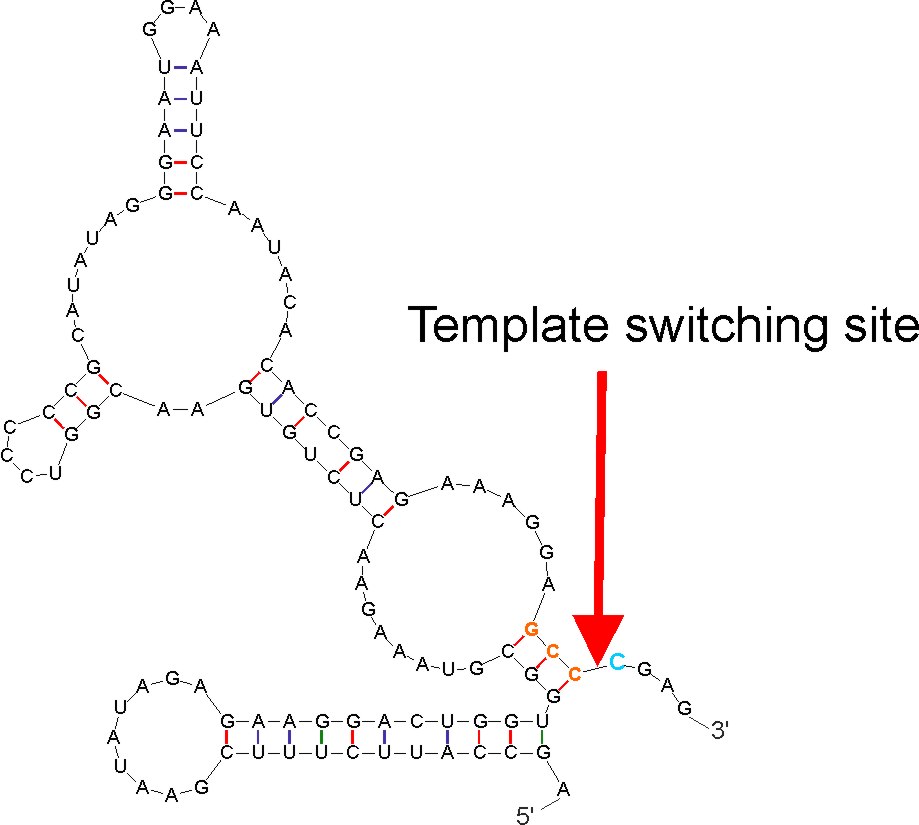


Chimeras 8 & 9


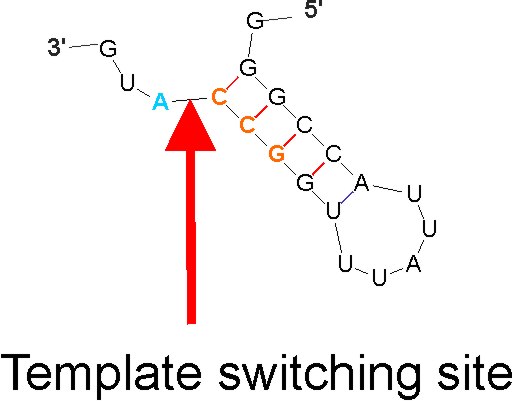


Chimera 7


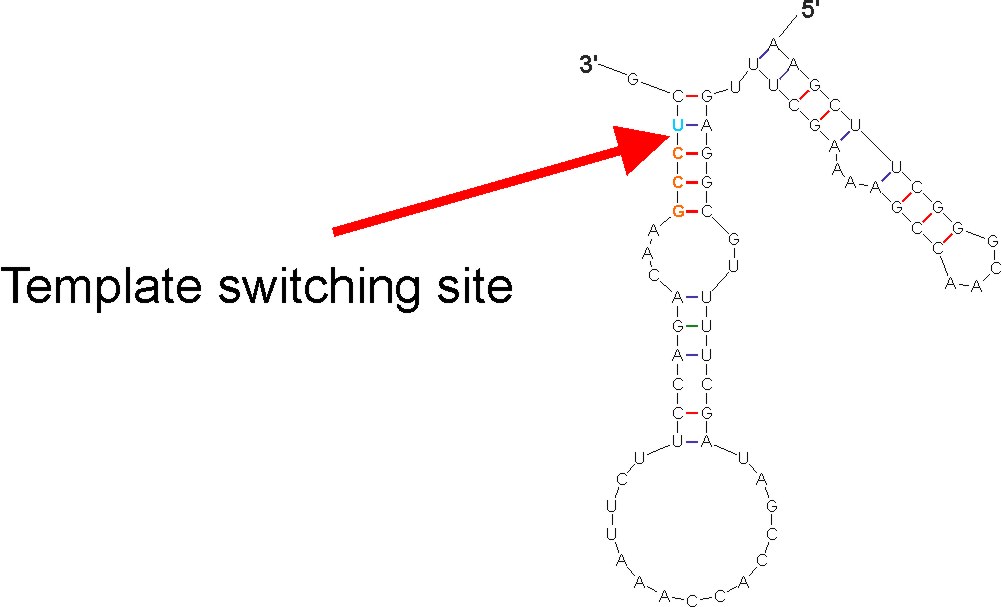


Chimeras 5 & 6


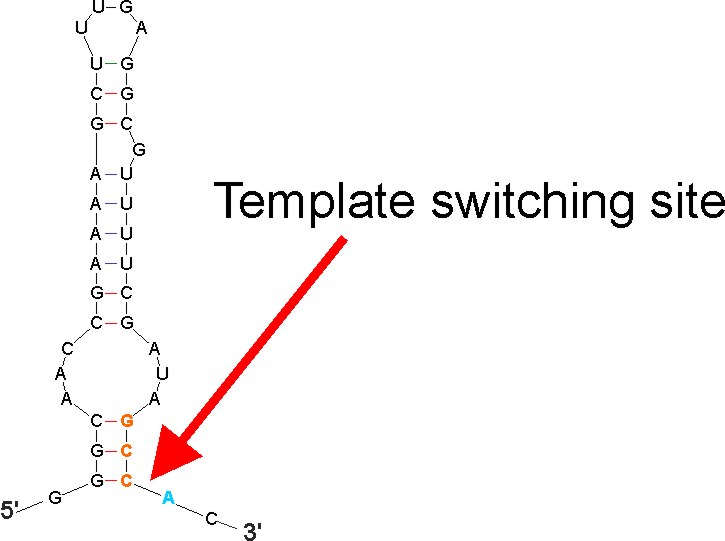


Chimera 2


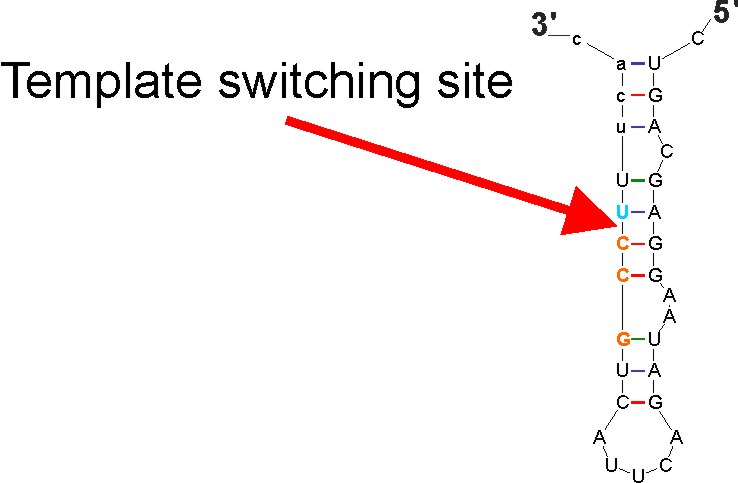


Chimera 1


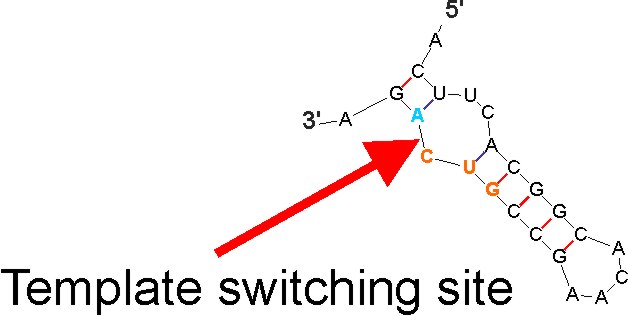


Chimera 34 (MgSINE)


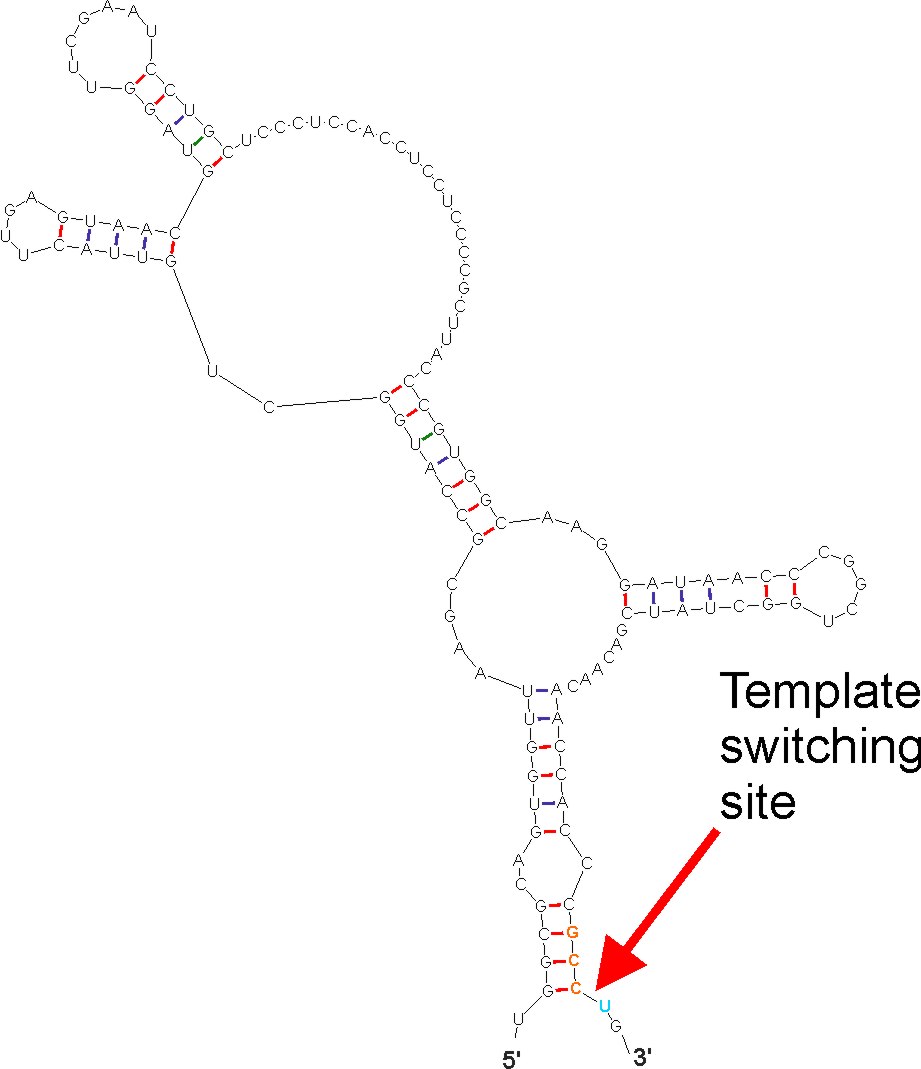


Chimera 32 (MgSINE)


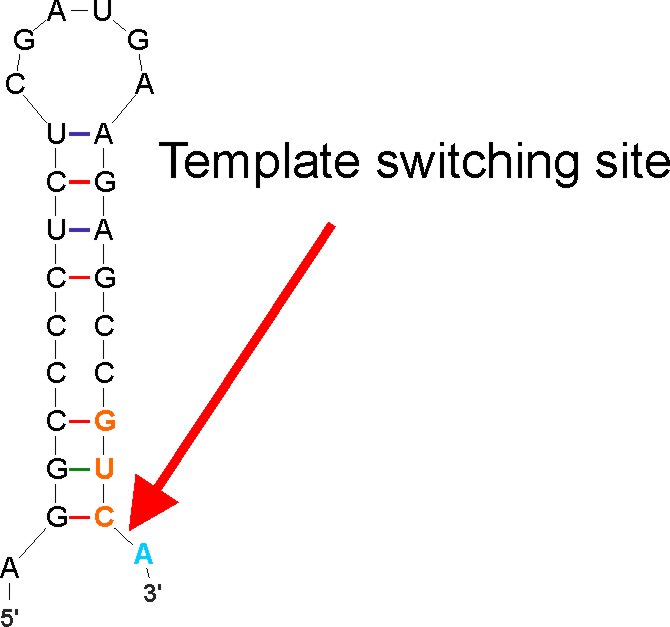

Supplement: Additional file 3 — Putative RNA secondary structure elements, predicted upstream template switching sites. RNA secondary structure features, predicted upstream template switching sites using Mfold version 3 software. [file 1471-2164-8-360-S3.doc]
