# Supplementary material for: Tripartite chimeric pseudogene from the genome of rice blast fungus Magnaporthe grisea suggests double template jumps during long interspersed nuclear element (LINE) reverse transcription
Source: BMC Genomics. 2007 Oct 8;8:360. doi: 10.1186/1471-2164-8-360 (PMC2104539; doi:10.1186/1471-2164-8-360)
Supplement: Additional file 4 — Oligonucleotides used in the present study. Sequence information for the primers used for RT-PCR experiments in the present study. [file 1471-2164-8-360-S4.doc]

Oligonucleotides used in the present study.

-Primers Wfor, Wrev, 1 and 3 are specific to WEIRD sequence.

Wfor TACGTGGCAGGCTGGGTATC

Wrev CTCTAACGGACAGGCCAGCTA

1 CTC TTC AAC CTG TCG CCC TA

3 GGA AGG CTA GAC AAT ACG GTT

-Primers MGLfor, MGLrev, 2 and 4 are specific to MGL retrotransposon.

MGLfor ACGCCGCAAATTCTTCCA

MGLrev CTATCGAAAACGCCTCAAAGCT

2 GCG CGC GTT CTA TCT TCT T

4 GGC AGT AAG TCT ATT CCT CGT C

-Primers q1-q6 were designed to amplify individual MINE elements using specific WEIRD/MGL junctions.

q1 GCTGAAGTAGTTGGTCCTTGCAT

q2 AGCTGGCCTGTCCGTTAGAG

q3 GCAGTTCCTCGCCTTGCAT

q4 TAGCTGGCCTGTCCGTTAGAG

q5 CCGCTCTGGCCTTGCA

q6 CCGTTAGAGGGTCTGGTCCAA
